# Obesity modulates the cellular and molecular microenvironment in the peritoneal cavity: implication for ovarian cancer risk

**DOI:** 10.3389/fimmu.2023.1323399

**Published:** 2024-01-09

**Authors:** Amanda A. Shea, Connie Lynn Heffron, Joseph P. Grieco, Paul C. Roberts, Eva M. Schmelz

**Affiliations:** ^1^ Department of Human Nutrition, Foods and Exercise, Virginia Tech, Blacksburg, VA, United States; ^2^ Department of Biomedical Sciences and Pathobiology, Virginia Tech, Blacksburg, VA, United States; ^3^ Graduate Program in Translational Biology, Medicine, and Health, Virginia Tech, Blacksburg, VA, United States

**Keywords:** obesity, ovarian cancer, inflammation, B cells, MDSCs, ascites, adhesion, tumor microenvironment

## Abstract

**Introduction:**

Abdominal obesity increases the risk of developing ovarian cancer but the molecular mechanisms of how obesity supports ovarian cancer development remain unknown. Here we investigated the impact of obesity on the immune cell and gene expression profiles of distinct abdominal tissues, focusing on the peritoneal serous fluid (PSF) and the omental fat band (OFB) as critical determinants for the dissemination of ovarian metastases and early metastatic events within the peritoneal cavity.

**Methods:**

Female C57BL/6 mice were fed a low-fat (LFD) or a high-fat diet (HFD) for 12 weeks until the body weights in the HFD group were significantly higher and the mice displayed an impaired glucose tolerance. Then the mice were injected with the murine ovarian cancer cells (MOSE-LTICv) while remaining on their diets. After 21 days, the mice were sacrificed, tumor burden was evaluated and tissues were harvested. The immune cell composition of abdominal tissues and changes in gene expression in the PSF and OFB were evaluated by flow cytometry and qPCR RT2-profiler PCR arrays and confirmed by qRT-PCR, respectively. Other peritoneal adipose tissues including parametrial and retroperitoneal white adipose tissues as well as blood were also investigated.

**Results:**

While limited effects were observed in the other peritoneal adipose tissues, feeding mice the HFD led to distinct changes in the immune cell composition in the PSF and the OFB: a depletion of B cells but an increase in myeloid-derived suppressor cells (MDSC) and mono/granulocytes, generating pro-inflammatory environments with increased expression of cyto- and chemokines, and genes supporting adhesion, survival, and growth, as well as suppression of apoptosis. This was associated with a higher peritoneal tumor burden compared to mice fed a LFD. Changes in cellular and genetic profiles were often exacerbated by the HFD. There was a large overlap in genes that were modulated by both the HFD and the cancer cells, suggesting that this ‘genetic fingerprint’ is important for ovarian metastases to the OFB.

**Discussion:**

In accordance with the ‘seed and soil’ theory, our studies show that obesity contributes to the generation of a pro-inflammatory peritoneal environment that supports the survival of disseminating ovarian cancer cells in the PSF and the OFB and enhances the early metastatic adhesion events in the OFB through an increase in extracellular matrix proteins and modulators such as fibronectin 1 and collagen I expression as well as in genes supporting growth and invasion such as Tenacin C. The identified genes could potentially be used as targets for prevention strategies to lower the ovarian cancer risk in women with obesity.

## 1 Introduction

Obesity has been linked to the etiology of coronary heart disease, hypertension, and diabetes and has also been associated with the increased risk of many cancers including endometrial, pancreatic, prostate, breast, colon and rectum, liver, and other cancers (1). However, inconsistent approaches in defining obesity, variations in study methodology including the use of different adipose tissues (e.g., visceral versus subcutaneous), ambiguity of age, menopausal status and the use of hormone replacement therapy, in addition to the heterogenous histology of ovarian cancer, have contributed to diverging risk assessments of obesity and ovarian cancer. An association of ovarian cancer risk and body mass index (BMI) of >30kg/m^2^ has been reported although the risk increase was generally low (2) and highly affected by the cancer subtype (3), and variables such as hormone therapy and smoking (2, 4, 5). The distribution of adiposity also may affect the cancer risk. Indeed, abdominal adiposity was found to be a risk predictor (6, 7) that raised ovarian cancer risk independent of BMI (8) and increased ovarian cancer mortality (9), suggesting an impact of abdominal adipose tissues on ovarian cancer development and progression. While interactions between the adipose tissues and ovarian cancer cells have been shown to support cancer cell survival (10, 11), the mechanisms of how obesity increases ovarian cancer risk have not been clearly delineated.

Metastatic ovarian cancer cells exfoliate from the primary tumor and disseminate throughout the peritoneal cavity via the flow of the peritoneal serous fluid (PSF) (12). Their primary metastatic site is typically the omentum (omental fat band, OFB), a visceral adipose tissue that serves as a filter for the PSF and functions as secondary immune organ for immuno surveillance within the peritoneal cavity. The OFB is interspersed with cellular aggregates of stromal and immune cells such as T cells, B cells, and macrophages, called “milky spots” (13). Disseminating tumor cells are both actively recruited to the OFB via IL-6 and IL-8 secretion (11) and passively filtered from the PSF, adhering to omental milky spots within hours of implantation (11, 14). Both omental adipocytes and milky spots contribute to cancer cell recruitment, survival and metastatic outgrowth (10, 11); therefore, the OFB is typically removed during surgical tumor debulking to slow disease progression.

We have previously shown that individual fat depots in the peritoneal cavity contain distinct immune cell populations (15) that may differentially contribute to ovarian cancer risk in either a protective or tumor-promoting manner. Indeed, the parity-mediated decrease in ovarian tumor burden was associated with a significant immunological shift in resident leukocyte populations in the OFB that supported the development of a refractory microenvironment (16). Therefore, understanding the changes in the cellular and secretory composition of abdominal adipose tissues and the metastatic transport media PSF can offer insights into the role of the abdominal microenvironment in ovarian cancer metastasis. Here, we investigated how diet-induced obesity modulates the composition of the OFB and select peritoneal tissues to generate permissive conditions for ovarian cancer metastases. Using a murine serous ovarian cancer cell model, we examined the correlation between increased body weight, peritoneal dissemination of ovarian cancer, and differential shifts in the immunological and gene expression profiles of critical microenvironments within the peritoneal cavity and additionally identified events that can promote the establishment of a pro-tumorigenic environment. Since ovarian cancer has one of the highest incidence-to-death ratios due to late detection when the tumor has already metastasized (17), understanding the dynamics of the pre-metastatic tumor microenvironment as well as characterizing the cellular and molecular changes involved in the generation of a permissive niche for ovarian metastases are critical steps for elucidating potential therapeutic targets and developing novel strategies to prevent or suppress ovarian cancer metastasis in high-risk groups.

## 2 Materials and method

### 2.1 Mice and diets

Female nine-week-old C57BL/6 mice (Harlan Laboratories) were singly housed in a controlled environment (12h light/dark cycle at 21°C) with free access to food and water. After acclimatization for one week, the mice were randomly assigned to four groups (n=10 each). Two groups each were placed either on a low-fat (AIN-93G rodent diet from Dyets with 7% corn oil; 17% of total kcal from fat) or a high-fat diet (AIN-93G rodent diet with 7% corn oil and 16.5% lard; 45% of kcal from fat). After 12 weeks on the research diets, the mice were subjected to body composition analyses using a Bruker mini spec LF90NMR analyzer to confirm diet-induced changes in their body composition and insulin response. This study was conducted in accordance with the NIH guidelines for Vertebrate Animal Use and was approved by the Virginia Tech Institutional Animal Care and Usage Committee.

### 2.2 Glucose tolerance test

After 12 weeks of dietary treatment, mice were fasted overnight and i.p. injected with 2 mg pharmaceutical grade glucose/g body weight in a 10% solution of PBS. Blood was then sampled six times over a two-hour period (at time point 0, 15, 30, 60, 90, and 120 minutes). Approximately 5 μl of blood was collected at each time point via tail vein nicks. A hand-held glucometer was used to measure blood glucose.

### 2.3 Cell lines

The mouse ovarian surface epithelial (MOSE) cell model utilized in this study was developed from C57BL/6 mice and has been extensively characterized (18–22). The expression of several fallopian tube markers (19) suggests that the MOSE cells represent the most detrimental serous ovarian cancer. Highly aggressive, tumor-initiating variants (MOSE-L_TIC_ *_v_*) were isolated following i.p. passage of MOSE-L cells in C57BL/6 mice and subsequently maintained in tissue culture. The MOSE-L_TIC_ *_v_* were transduced with firefly luciferase (FFL)-encoding lentiviral particles (GeneCopoeia) to facilitate live *in vivo* imaging of cancer cell outgrowth; the FFL gene served also as a tumor-specific reporter gene. MOSE-L_TIC_ *_v_*, injected i.p. into female C57BL/6 mice rapidly disseminate throughout the peritoneal cavity to the OFB with mice reaching defined endpoints within 21 days (16). MOSE-L_TIC_ *_v_* cells were maintained in high glucose Dulbecco’s Modified Eagle Medium (Invitrogen), supplemented with 4% fetal bovine serum (Atlanta Biological), 100 mg/ml penicillin and streptomycin and 4 μg/ml puromycin to maintain FFL expression.

### 2.4 Tumor induction

After 12 weeks of dietary regimen, when significant changes in body weight and glucose tolerance were observed, one group of mice from each dietary regimen (n=10 per group) was injected i.p. with 1x10^4^ MOSE-L_TIC_ *_v_* cells in 300 μl sterile calcium- and magnesium-deficient phosphate buffered saline (PBS^-/-^) while the control groups received injections of 300 μl PBS^-/-^. All mice were maintained on their respective diets for 21 days post-injection. Then mice were sacrificed by CO_2_ asphyxiation. To quantify the tumor burden, PCI scores were determined as described previously (14, 16). Briefly, tumor burden (number and size, vascularization) was determined in the peritoneal lining, diaphragm, ovaries, lesser and greater omentum, liver, stomach, pancreas, mesentery, small intestine, and colon. Relative PCI scores in the OFB were further validated by qRT-PCR analysis of FFL gene expression.

### 2.5 Adipose tissue, peritoneal serous fluid, and blood collection

Three intra-abdominal white adipose tissues (WAT) were harvested: the OFB, parametrial white adipose tissue (pmWAT) and retroperitoneal white adipose tissue (rpWAT). The OFB (visceral fat depot) was chosen as it is a primary site of ovarian cancer metastasis and because of its importance in the immunosurveillance of the peritoneal cavity. pmWAT and rpWAT served as control non-visceral abdominal fat depots with distinct immune and progenitor populations (15); due to its abundance in obese mice, pmWAT (or epididymal WAT in male mice) is the most used tissue in other studies. The PSF was included as an established immunologically active microenvironment and vehicle of peritoneal cancer cell dissemination; blood was used as an indicator of systemic effects.

All tissues were individually harvested, weighed, and rinsed with PBS^-/-^. To ensure there was no pancreatic contamination, the OFB samples were also tested for buoyancy. Each sample was then processed for flow cytometry or placed into RNAlater (Qiagen) and stored at -80°C. Cells in the PSF were collected via peritoneal lavage with 5 ml of PBS^-/-^. The effluent was centrifuged, subjected to erythrocyte lysis (155 mM NH_4_Cl, 10 mM KHCO_3_, 0.1 mM EDTA) and processed for flow cytometry or stored for RNA extraction. Blood was collected via heart puncture and centrifuged at 1100 rpm for 10 minutes. The pellet was subjected to erythrocyte lysis three times and then processed for analysis.

### 2.6 Tissue digest

The stromal cells (stromal vascular fraction, SVF) from each adipose depot (n=10) were isolated from digested tissue according to standard protocols (14–16). Briefly, the OFB was digested in GKN-buffer containing 1.8 mg/ml collagenase IV, 10% FBS, and 0.1 mg/ml DNase. PmWAT and rpWAT digest buffer contained a 1:1 ratio of Krebs-Ringer bicarbonate buffer and collagenase solution (1 mg collagenase I, 10 mg BSA, and 2 mM CaCl_2_ in 1 ml PBS). Following digest at 37°C for 45 min, cells were passed through a 40 μm cell strainer, and erythrocytes were lysed.

### 2.7 Flow cytometric analysis

SVF suspensions from the tissues of five mice per group were washed in flow buffer (2% BSA in PBS^-/-^), blocked with Fc block (BD Biosciences) for 10 minutes at 4°C, rinsed and incubated with fluorophore-labeled antibody combinations (available upon request) for 20 min at 4°C. Fluorophore-labeled antibodies specific for mouse CD45, CD11b, CD11c, F4/80, Ly6C, CD4, CD5, CD44 CD62L, B220, CD19, CD93, NK1.1, Ly6C, and Ly6G were obtained from eBioscience. CD3 and CD8 antibodies were obtained from BD Biosciences. Prior to analysis, cells were washed twice and re-suspended in PBS^-/-^ containing propidium iodide for dead cell exclusion. Viable cells identified via propidium iodide exclusion were gated based on forward/side scatter to exclude doublets and were then separated into CD45^+^ leukocytes and CD45^-^ stromal constituents. CD45^+^ cell was then further subdivided into specific leukocyte populations: CD19^+^ B cells, CD5^+^ or CD3^+^ T cells, CD11b^+^ mono- or granulocytes, NK.1.1^+^ CD3_-_ NK cells, NK.1.^+^ CD3^-^ NKT cells (see **Table 1** for sub-groups). Flow cytometry was performed on a FACSAria (BD Biosciences) and data was analyzed using Flowjo (TreeStar) software.

**Table 1:** Comparison of the CD45+ cells in the individual tissues of mice on a low-fat (LFD) or high-fat diet (HFD) (SEM in parenthesis).

			Blood		PSF		OFB		pmWAT		rpWAT	
		Markers	LFD	HFD	LFD	HFD	LFD	HFD	LFD	HFD	LFD	HFD
% CD45^+^		Percentage of viable cells	98.90 (0.13)	98.84 (0.26)	99.76 (0.14)	90.84 (8.16)	86.58 (2.31)	80.66 (2.67)	59.26 (6.04)	59.96 (3.68)	50.46 (2.64)	55.32 (3.89)
Percent of CD45+	B cells	CD19^+^	54.51 (3.42)	18.10^ (1.47)	56.94 (6.35)	32.24* (4.46)	51.40 (3.11)	44.26^a^ (3.10)	5.06 (0.41)	4.07 (0.57)	10.71 (1.49)	17.86 (4.58)
	T cells	CD5^+^	30.72 (3.37)	60.94^ (1.67)	13.94 (2.33)	15.83 (4.02)	29.78 (3.81)	28.26 (2.56)	3.55 (1.55)	10.36 (0.84)	6.57 (2.94)	10.51 (4.70)
	Other lymphoid cells	CD5^-^, (loSSC)	4.24 (0.33)	10.08^ (0.37)	1.97 (0.37)	4.68** (0.56)	7.82 (0.54)	10.53 (1.70)	28.38 (1.63)	29.47 (3.37)	26.61 (2.21)	26.01 (2.73)
	Mono/granulocytes	CD11b^+^, SSC variable	5.56 (0.87)	5.16 (0.42)	15.22 (1.63)	32.48* (4,74)	11.75 (2.32)	15.40 (2.79)	41.94 (2.18)	50.28* (2.52)	48.92 (8.10)	44.86 (5.22)

*p<0.05; **p<0.01; ^p<0.001; ^a^ p=0.088 compared to the LFD.

### 2.8 RNA Extraction and cDNA synthesis

Adipose tissues were homogenized in Qiazol (Qiagen) and PSF cells were lysed in RLT lysis buffer (Qiagen). RNA was purified using the RNeasy Lipid Tissue Kit (Qiagen), according to manufacturer’s instructions. RNA concentration was determined using a NanoDrop1000 spectrophotometer. RNA (n= 5 per tissue) was then subjected to the iScript cDNA synthesis system (Biorad) according to the manufacturer’s protocol.

### 2.9 RT2 profiler PCR array

RT2 Profiler PCR arrays were specific to genes involved in acute phase inflammatory and humoral immune responses, targeting cytokines, chemokines and their receptors, and regulators of cytokine metabolism and synthesis (>350 genes, see **Supplementary Table S1**). cDNA for each group (5 mice each) was pooled from equal amounts of RNA from each sample, totaling 1μg RNA for one plate. The SABiosciences RT^2^ Profiler PCR Array (PAMM-3803E-12) was conducted according to the manufacturer’s protocol with the ABI 7900HT (Applied Biosystems). Data was analyzed using the SABiosciences RT^2^ Profiler PCR Array data analysis program. Genes with differences of at least a 2-fold change were included in lists of differentially expressed genes.

### 2.10 Quantitative real-time PCR

qRT-PCR was performed with 12.5ng cDNA per sample using gene-specific SYBR Green primers (primer sequences are available upon request) designed with Beacon Design software. SensiMix SYBR and Fluorescein master mix (Bioline) was used in a 15 μL reaction volume. qRT-PCR was performed for 42 cycles at 95°C for 15 sec, 60°C for 15 sec, and 72°C for 15 sec, preceded by a 10 min incubation at 95°C on the ABI 7900HT (Applied Biosystems). Melt curves were performed to ensure fidelity of the PCR products. RPL19 was utilized as the housekeeping gene and the ΔΔCt method was used to determine fold differences (23).

### 2.11 Immunofluorescence

Adherent MOSE-L_TIC_ *_v_* cells were grown on coverslips; pre-formed spheroids were allowed to adhere for 4, 8, and 12 h onto glass-bottom cell culture dishes (Cellvis) and were then washed away prior to fixation, leaving only secreted extracellular matrix (ECM) on the plates.

Proteins were immunostained with primary antibodies against Collagen Type I (Invitrogen) and Fibronectin (Abcam), along with appropriate FITC-conjugated rabbit and TRITC-conjugated mouse secondary antibodies. Coverslips with adherent cells were mounted with Prolong gold antifade mounting medium with DAPI and imaged using a Nikon 80*i* fluorescent microscope. Images were processed using Adobe Photoshop CS6®.

### 2.12 Statistical analysis

Data is expressed as mean ± standard error of mean (SEM). Student’s t-tests were used to compare two groups. Two-way ANOVA with post-tests were used to compare multiple tissues or more than two groups. Differences were considered statistically significant at p<0.05.

## 3 Results

### 3.1 Effect of diet on body weight and composition

Mice fed the high-fat diet (HFD) for 15 weeks gained more weight (25.66g *vs* 17.91g in the low-fat diet -LFD) and had significantly higher body weights compared to mice fed the LFD (42.97g *vs* 34.89g; p=0.0036) (**Supplementary Figure S1A**). Accordingly, mice fed the HFD had greater body fat percentages (27.28 ± 1.49% *vs* 20.14 ± 2.09% on the LFD; p<0.05) (**Supplementary Figure S1B**). Blood glucose levels were higher in the HFD group at all time points following time 0 (p<0.05) (**Supplementary Figure S1C**), indicating an impaired glucose tolerance. However, as fasting glucose remained normal, the mice did not display all symptoms of metabolic syndrome, confirming earlier reports that in contrast to male mice, female mice on a HFD are less likely to develop the full spectrum of the metabolic syndrome (24).

**Figure 1:**
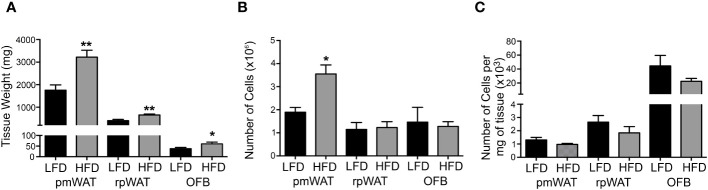
Effect of the diet on tissue weight and cellularity. 12 weeks of feeding a low-fat (LFD) or high-fat diet (HFD) affected **(A)** the wet tissue weight, **(B)** the total number of cells per tissue, and **(C)** the Number of cells per mg of tissue. * p<0.05; **p<0.01 compared to the LFD.

As expected, the weights of all individual WATs were significantly higher in mice fed the HFD (**Figure 1A**) but only the pmWAT showed a significant increase in SVF cell number in the HFD group (**Figure 1B**). The number of cells per mg of tissue did not significantly change in any tissue in response to the diet (**Figure 1C**), suggesting that the HFD did not induce a net influx of cells above the observed tissue expansion.

### 3.2 Impact of HFD on resident peritoneal leukocytes

We have previously shown that the individual abdominal fatpads are characterized by distinct leukocyte populations (15). To determine how the HFD affects these tissues, we identified HFD-induced changes in leukocyte populations within select abdominal WATs as well as in the blood and PSF. As shown in **Table 1**, 98% of cells found in the blood and PSF were leukocytes staining positive for the pan-leukocyte marker CD45. The percentage of CD45^+^ cells was lower in the WATs than the PSF (p<0.001 *vs* pmWAT and rpWAT), concurrent with their function as reservoirs for the stem- and progenitor cells in the stromal vascular fraction (SVF). The OFB contained a greater proportion of leukocytes than the other abdominal WATs (p<0.01 and p<0.001 *vs* pmWAT and rpWAT, respectively) albeit less than the PSF (p<0.001). This is consistent with the OFB serving as a unique lymphoid organ (25). The HFD did not significantly alter the percentage of CD45^+^ cells in any tissue but changed the proportion of specific leukocyte subsets. A significant HFD-induced decrease in the CD19^+^ B cells was observed in blood, PSF and the OFB while their already very low proportion in the pmWAT and rpWAT did not change. There was a trend towards increased proportions of T cells in all tissues except the OFB after feeding the HFD; this was significant only in blood, confirming that the HFD contributes to the systemic low-grade chronic inflammation commonly associated with obesity (26). Additionally, the HFD group had increased CD5- lymphoid cells in the blood, PSF and the OFB but not in the pmWAT and rpWAT. An increased proportion of monocytes/granulocytes was observed in all tissues but blood (significant in PSF and pmWAT). Thus, in the more immunologically active areas of the peritoneal cavity (PSF and OFB), the HFD led to a decrease in B cells, with increases noted for other lymphoid cells and monocytes/granulocytes. In contrast, the pmWAT and rpWAT were more resistant to HFD-induced changes in the immune cell composition despite the increase in total SVF cell numbers.

### 3.3 HFD-induced changes in gene expression in select tissues

**Figure 2:**
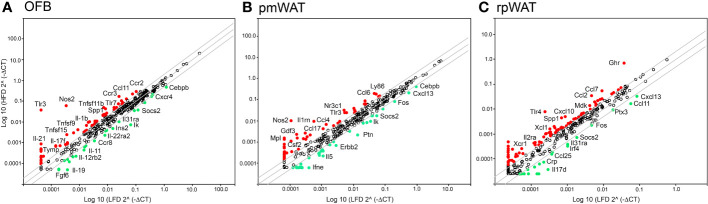
Changes in gene expression levels by the high-fat diet (HFD) as determined by RT2 Profiler PCR arrays in the **(A)** omental fat band (OFB), **(B)** parametrial white adipose tissue (pmWAT), and **(C)** retroperitoneal white adipose tissue (rpWAT). Data is expressed as the log10 of the expression level of each gene in the test group (HFD) versus the corresponding value in the LFD. Red circles represent genes that increased by twofold or greater in the HFD group, green circles represent genes that decreased by twofold or greater.

The immune cell composition of tumors is very heterogenous and dependent not only on the tumor type but also on their molecular subtypes, limiting their prognostic value (27). Thus, to characterize the HFD-mediated changes in the peritoneal signaling milieu more comprehensively, RT2 Profiler PCR arrays were performed with RNA extracted from the OFB, pmWAT and rpWAT (ΔCT values for all tissues are shown in **Supplementary Table S1**). The expression patterns represent the whole tissue and, thus, are reflective of both adipocytes and cells comprising the SVF. Several genes were upregulated in all three adipose tissues upon HFD feeding (*Areg*, *Ccl2, Ccl7, Ccr2, Csf2, Il7r, Spp1, Tlr1, Tnfsf9, Tnfrsf11b*, *Xcr1*) whereas only *Fos*, *Il17d*, and *Socs2* expression were decreased in all tissues (**Figure 2**). Genes regulating immune response and inflammation including many cyto- and chemokines and their receptors were expressed at higher levels in the OFB than in the pmWAT and rpWAT, likely a result of the higher cell count and CD45+ cell content of the OFB. Differences in gene expression between the pmWAT and rpWAT were minimal (**Supplementary Table S1**).

In the OFB, the HFD led to increased gene expression of many cyto- and chemokines and their receptors, which may contribute to a sustained pro-inflammatory microenvironment. Expression of genes regulating adhesion and cell growth (*Apol7a*, *Blnk, Bmp7, Fgf5,7, Gdf5, Itih4, Nampt, Ptafr, Reg3a, Spp1, Tymp*), and TNF superfamily members were also increased by the HFD while genes that are involved in the regulation of monocyte chemotaxis (*Ccl27a, Ccr7*, *Ccr8*), suppression of cytokine synthesis and signaling (*Il11, Socs2*) and regulation of apoptosis (*Ccr8, Il19*) were reduced (**Figure 2A**). Gene expression changes in the pmWAT (**Figure 2B**) and rpWAT (**Figure 2C**) may also contribute to maintenance of a pro-inflammatory microenvironment (*Ccl4*, *Ccl7*, *Ccl17*, *Il2ra*, *Ly86*, *Tlr4*), cell survival (*Ghr*, *Mdk*, N*r3c1*), and protection of stem cells from apoptosis (*Gdf3*); however, the expression levels and magnitude of changes were lower than in the OFB and there was little overlap of gene expression changes between the select adipose tissues (see **Supplementary Table S1**). Thus, the HFD resulted in changes to the cellular composition and molecular expression signature of the OFB which may support a pro-inflammatory, pro-adhesion and pro-growth microenvironment that is more conducive for cancer cell adhesion, survival, and progression, while other tissues had less notable changes.

### 3.4 The HFD potentiates peritoneal tumor burden of ovarian cancer

**Figure 3:**
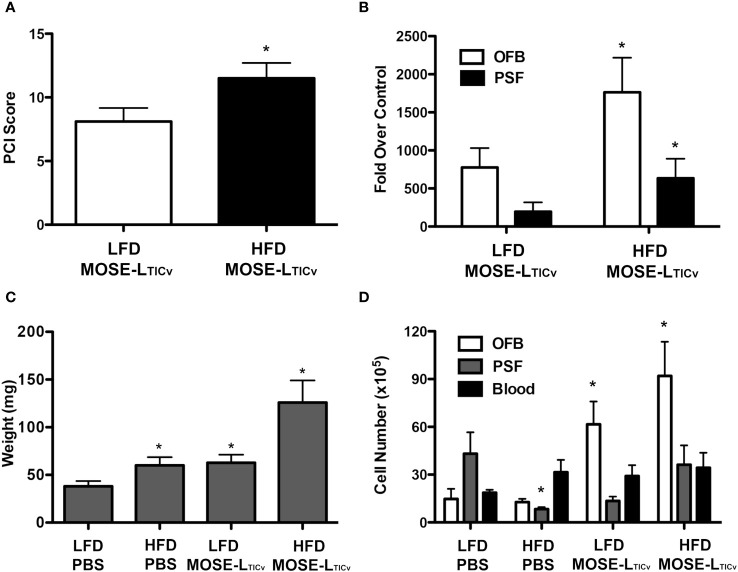
Tumor burden of mice 21 days post injection of MOSE-L_TIC_ *_v_* cells. **(A)** PCI score of low-fat (LFD) high-fat diet (HFD) groups after MOSE-L_TIC_ *_v_* injection. **(B)** FFL gene expression in the omental fat band (OFB) and peritoneal serous fluid (PSF) of mice following MOSE-L_TIC_ *_v_* injection as compared to the PBS group on each diet; *p<0.05 compared the LFD. **(C)** Average OFB weight per mouse; ^*^ p<0.05 compared to LFD. **(D)** Average number of cells isolated from each tissue. *significant at p<0.05 over the corresponding LFD group, or the corresponding PBS controls.

To determine if the observed HFD-mediated changes in the abdominal microenvironments promote ovarian cancer metastasis, we injected female C57BL/6 mice i.p. with the syngeneic MOSE-L_TIC_ *_v_* cells or PBS (control group) upon confirmation of the HFD-induced changes in body weight, body fat percentages, and aberrant glucose tolerance. Tumor cell implantation had no effect on body weight or percent body fat in either diet group (data not shown) likely due to the rapid development of lethal disease in this model. As shown in **Figure 3A**, there was a significant increase in the tumor burden in mice fed the HFD (p<0.05). Concordantly, the expression levels of FFL, used here as a tumor cell reporter gene expressed only in MOSE-L_TIC_ *_v_* cells, was significantly higher in the OFB of mice fed the HFD (**Figure 3B**). Importantly, this increase in FFL expression was also observed in the PSF, indicating that the HFD-induced changes also increased the number of the non-adherent, disseminating metastatic cancer cells in the peritoneal cavity. Macroscopically, the OFBs in the HFD-MOSE-L_TIC_ *_v_* group were composed almost entirely of solid tumor, with minimal adipose tissue remaining. Despite this loss of fat, OFBs in HFD-MOSE-L_TIC_ *_v_* were significantly heavier than in LFD-MOSE-L_TIC_ *_v_* (**Figure 3C**) due to cancer cell recruitment and expansion; while the OFBs from LFD-MOSE-L_TIC_ *_v_* were also heavily infiltrated with tumors, these were of smaller size. This correlates well with the larger total cell number detected in the OFB in the HFD-MOSE-L_TIC_ *_v_* group (**Figure 3D**) that includes tumor, immune and stromal cells and corresponds to the higher tumor burden. The total cell number in the PSF was reduced by the HFD but increased in the HFD-MOSE-L_TIC_ *_v_* group. No increases in cell numbers in blood were noted after the MOSE-L_TIC_ *_v_* injections.

### 3.5 Cancer-associated changes in the peritoneal cavity

#### 3.5.1 Changes in immune cell composition

**Figure 4:**
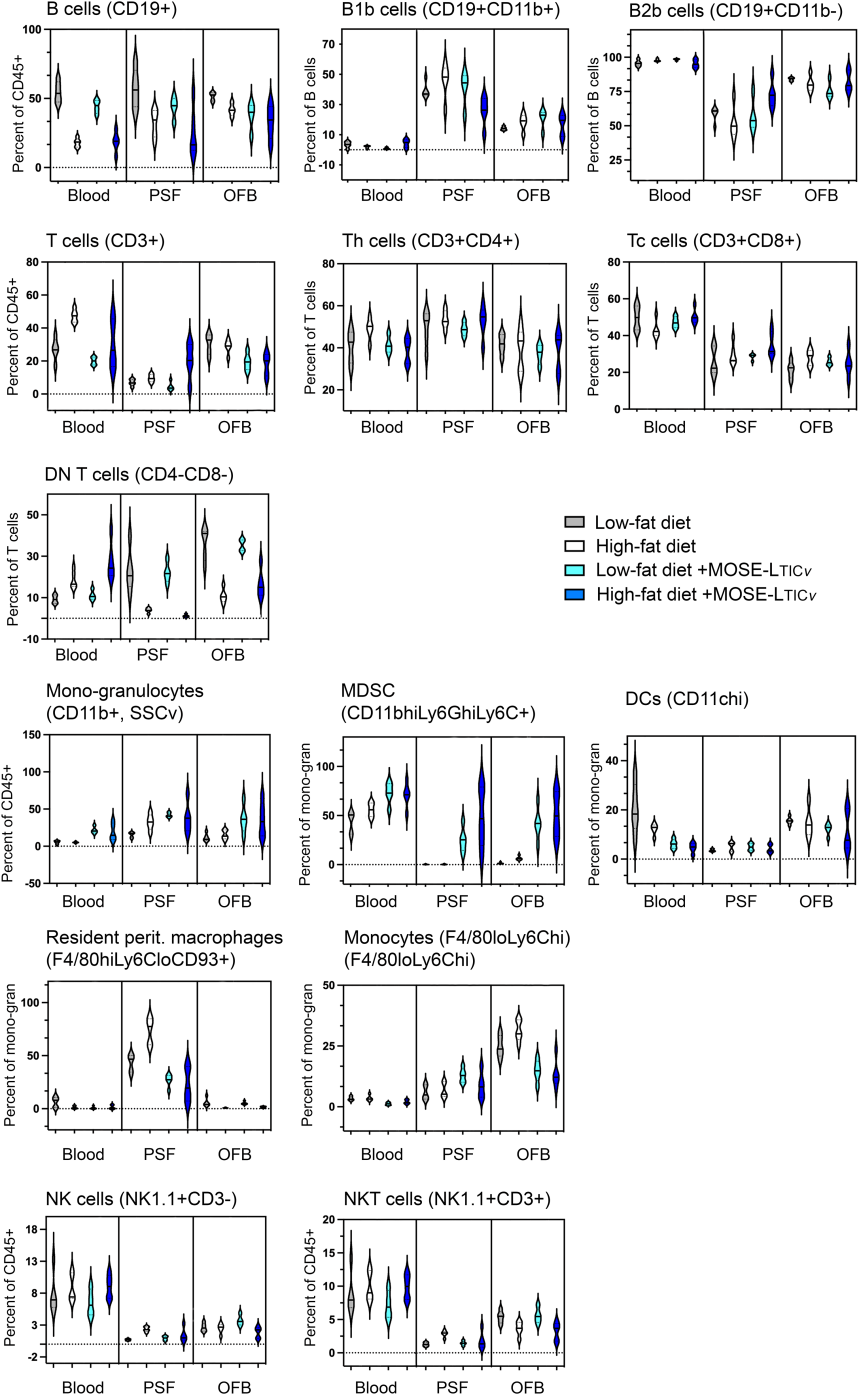
Changes in immune cell populations by the diet and the cancer cells. Flow cytometric analyses were performed in blood, peritoneal serous fluid (PSF) and the omental fat band (OFB).

We aimed to identify common cellular and molecular events induced by both the HFD and the cancer cells which would suggest the generation of a permissive niche. First flow cytometric analysis was used to characterize changes in the main immune cell populations in the selected adipose tissues, PSF, and blood as a consequence of the diet and ovarian cancer presence in the peritoneal cavity. While both diet- and cancer-induced shifts were noted in the PSF, blood, and OFB (**Figure 4**; **Supplementary Table S2A**), the pmWAT and the rpWAT were largely resistant to changes with both conditions (**Supplementary Table S2B**). The overall population of CD45^+^ cells remained stable in the presence of the cancer cells (**Supplementary Table S2A**) although the proportions of different cell types within the leukocyte population shifted. There was a reduction in the CD19^+^ B cell populations in the blood and the PSF by the cancer cells that was less pronounced than the reduction induced by the HFD; in contrast, the depletion of B cells in the OFB by the cancer cells was more prominent than the HFD-induced changes. A subsequent analysis showed mainly B2 cell with little effect of the cancer cells or the HFD on the proportions of either sub-population. There was little additional effect of the HFD combined with cancer cells (**Figure 4**).

The T cell percentage and the T cell sub-populations were not affected by the cancer cells alone; tissue specific increases (in the PSF) or decreases (in the OFB) were observed in the combined HFD/MOSE-L_TIC_ *_v_* group. Most T cells in the PSF and OFB were Th cells while more Tc cells were found in the blood, and CD4^-^CD8^-^ DN T cells were the predominant T cell type in the pmWAT and rpWAT (**Figure 4**; **Supplementary Table S2A**). While there were little changes in CD4^+^ and CD8^+^ T cell subpopulations, the DN T cells were increased in the blood by the combined treatments but significantly reduced in the PSF and the OFB which appears in the latter tissues to be little affected by the cancer cells.

Tissue-specific responses were also noted in the mono-granulocyte category. The cancer cells were associated with significant increases in the blood while the cancer cells increased mono/granulocytes in the PSF and the OFB with no additional effect of the combination treatment. The analysis of sub-populations showed a significant cancer cell-induced increase in myeloid-derived suppressor cells (MDSC) which was exacerbated by the HFD in the OFB and PSF; there were only small increases seen with the HFD alone. While there were no changes in total dendritic cells (DC), F4/80^hi^,Ly6C^lo^,CD93^+^ resident peritoneal macrophages were significantly increased in the PSF by the HFD but reduced in blood and OFB; cancer cell presence reduced the proportion of these cells in the PSF and the blood with no effect on the OFB. Tissue-specific responses were observed for NK cells. NKT cell proportions were significantly decreased only in the OFB by both the HFD and the cancer cells without a further combinatorial effect.

Together, these results demonstrate a differential response of the select tissues to both the HFD and the cancer cell injections: while the pmWAT and rpWAT were more resistant to changes and generally responded to the HFD only, the PSF and OFB exhibited significant and similar changes in specific sub-populations that together generate pro-inflammatory conditions. These changes often were exacerbated by the HFD and may contribute to a more permissive environment for disseminating cancer cells in the peritoneal cavity.

#### 3.5.2 Change in gene expression levels

**Figure 5:**
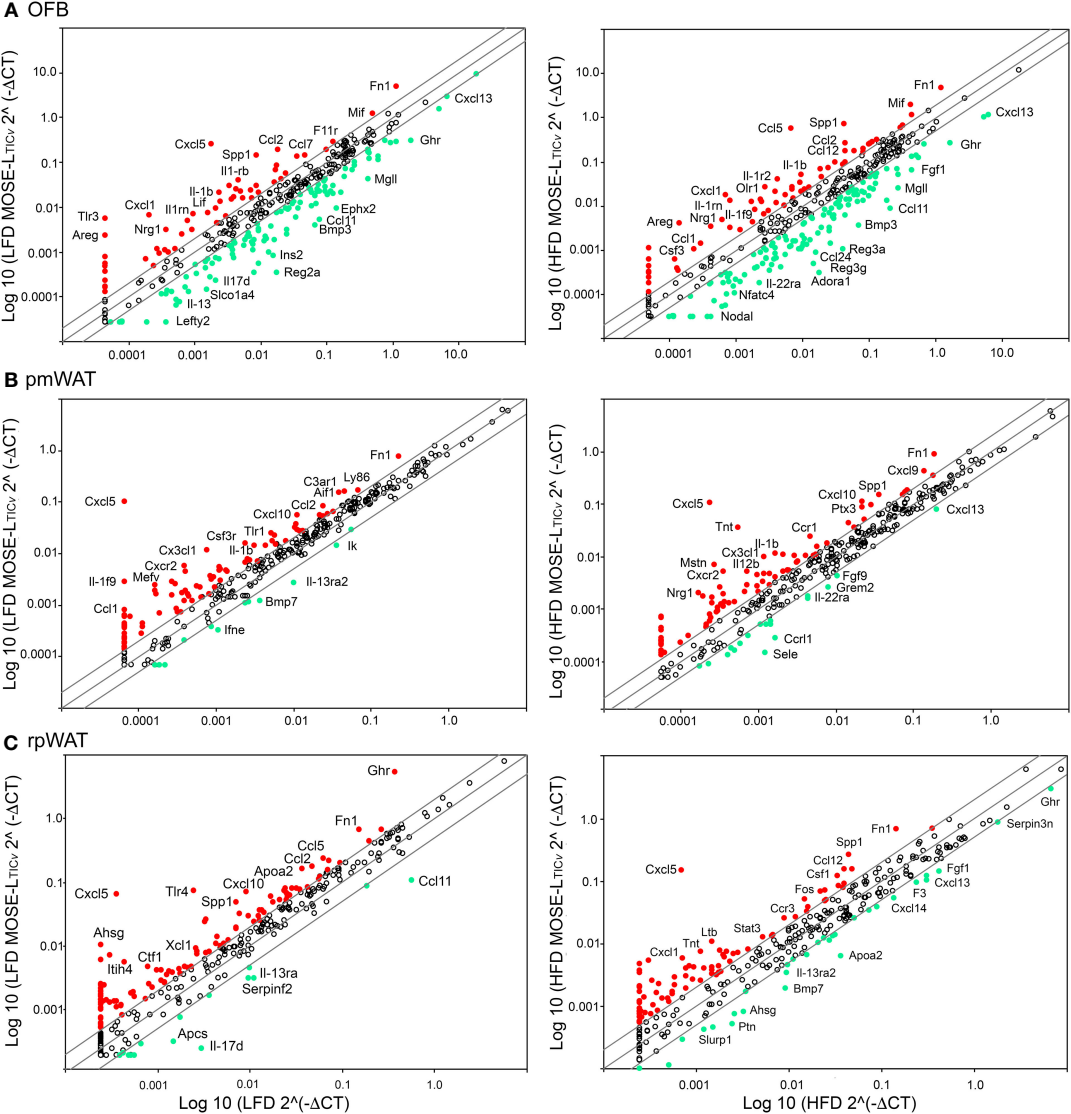
Changes in gene expression by cancer in peritoneal adipose tissues. RT2 Profiler PCR arrays were used to determine gene changes in mice fed the low-fat diet (LFD) (left panels) and the high-fat diet (HFD) (right panels). **(A)** Omental fat band (OFB), **(B)** parametrial white adipose tissue (pmWAT), and **(C)** retroperitoneal white adipose tissue (rpWAT). Red circles indicate genes that increased twofold or greater, green circles indicate genes that decreased twofold or greater.

To characterize changes in the signaling microenvironment within the peritoneal cavity as a consequence of the diet *and* cancer, we again used RT2 Profiler PCR arrays and compared the impact of the diet on gene expression to the effects of the cancer cells. Cancer cell presence increased the expression of many genes in all tissues investigated; this was more pronounced in the OFB than the pmWAT and rpWAT (**Figures 5A–C**) (see **Supplementary Table S1** for a complete list). Key cytokines and chemokines and their receptors in addition to growth regulators (*Areg, Csf1, Gdf6, Nrg1, Pdgfa, Spp1*), adhesion (*F11r, Fn1*) and stemness modulators (*Hdac7, Lefty1, Procr*), and genes associated with progressive disease (*Apoa2, Apol7a, Inhba, Mif, Nos2, Olr1, Prl, Tnfsf9, S100a8*) were upregulated in the OFB with the cancer cells. Genes often found to be epigenetically silenced or with low expression levels in ovarian or other cancers (*Adora1, Bmp3, Ccl6, Ephx2, Lefty2, Pf4, Socs2*) were downregulated by MOSE-L_TIC_ *_v_* in the OFB.

The combination of HFD and MOSE-L_TIC_ *_v_* further increased the expression levels of several cytokine and chemokines that contribute to a pro-inflammatory environment (see **Supplementary Tables S1, S3**). In addition, the expression of regulators of growth (*Nos2*, *Spp1*) and metabolism (*Ins1*, *Tymp*), as well as indicators of cancer progression (*Cer1*, *Fos*, *Saa4*) were exacerbated while genes such as *Adora1*, *Ccl6* that are often low expressed in cancer and genes that inhibit cancer growth and development (*Gdf1*, *Grem2*, *F2*, *Kng1*, *Ltb4r1*, *Nodal*) were further down-regulated compared to the LFD MOSE-L_TIC_ *_v_* group (**Supplementary Tables S1, S3**). These results suggest that the HFD alone can alter the gene expression profile in the OFB but can also exacerbate cancer-induced changes.

We also observed MOSE-L_TIC_ *_v_*-induced changes in the gene expression levels in the pmWAT and rpWAT that were enhanced when the mice were fed the HFD (**Figures 5B, C**). While these genes can also contribute to a pro-inflammatory and growth stimulatory environment, the changes in their expression levels were lower (**Supplementary Table S1**), indicating a less robust response of these tissues to both the HFD and the cancer cells. A complete list of genes that were affected by the HFD, by the cancer cells, or the combination of both can be found in **Supplementary Table S2**.

To confirm the changes in gene expression levels shown above that were obtained with the RT2 Profiler PCR arrays, we performed qRT-PCR on key inflammatory genes in the OFB, pmWAT, and rpWAT and extended our analysis to include genes that are critical for the survival of exfoliated cancer cells and for early metastatic processes such as adhesion and outgrowth. We noted both diet- and MOSE cell-dependent changes in gene expression levels in addition to tissue-specific responses. There were very few changes in response to the HFD in the pmWAT and rpWAT; only *Ccl2* was significantly elevated by the HFD. While the cancer presence alone did not elicit gene expression changes in these adipose tissues, there was a significant increase in *Ccl4* (in pmWAT only), *Ccl2* and *Il6* in both WATs in the HFD/MOSE_TIC_ *_v_* group (**Supplementary Table S4**). In contrast, genes in the OFB were highly responsive to both conditions (**Table 2**). The HFD alone did not alter the expression of the adipokines but significantly increased several cyto/chemokines or their receptors such as *Ccl2*, *Cx3cl1*, *Ccr5*, *IL1b* and *Tnfa*. Genes promoting adhesion (*FN1*, *Col1*) and invasion (*Tnc*) via extracellular matrix remodeling (*Mmp11, Pai1*) were also upregulated by the HFD.

**Table 2:** qPCR analyses of the OBF (ΔCT±SEM).

	Gene	LFD	HFD	LFD MOSE-L_TIC_ *_v_*	HFD MOSE-L_TIC_ *_v_*
Adipokines	Adiponectin	-3.35±0.23	-3.61±0.09	0.92±0.49^c^	1.98±0.82^g^
	Adipsin	18.75±0.08	18.46±0.08	20.30±0.15^b^	20.05±0.38^f^
	Leptin	1.20±0.22	0.86±0.35	5.70±0.53^c^	6.63±0.77^g^
	Resistin	-0.51±0.24	-0.60±0.17	3.98±0.64^c^	5.53±0.79^g^
	Visfatin	1.68±0.05	2.19±0.13	2.38±0.14^b^	2.53±0.16
Cyto- and chemokines	Ccl1	15.11±0.23	14.72±0.72	14.57±0.74	15.70±0.80
	Ccl2	5.83±0.30	3.98±0.17^c^	2.49±0.14^c^	2.01±0.17^g^
	Ccl4	9.27±0.37	8.29±0.27	7.56±0.43^a^	7.67±0.22
	Ccl5	2.71±0.11	2.53±0.32	3.03±0.34	3.39±0.93
	Ccr5	5.29±0.22	3.78±0.07^b^	4.35±0.29	4.80±0.38
	Cx3cl1	7.23±0.31	6.17±0.19^a^	7.10±0.07	7.19±0.27^d^
	Cxcl13	0.88±0.17	1.10±0.24	14.16±0.24^a^	12.99±0.56^g^
	Cxcr2	11.84±0.58	10.94±0.79	8.88±0.25^b^	8.82±0.32
	IFNγ	10.43±0.22	9.41±0.24	10.60±0.34	10.41±0.34
	IL-1β	11.48±0.51	9.75±0.22^b^	8.83±0.25^c^	8.44±0.21^e^
	IL-6	17.32±0.35	16.44±0.21	16.31±0.30	16.31±0.23
	IL-10	15.33±0.33	14.48±0.27	13.72±0.57^a^	14.25±0.18
	eNOS	9.00±0.23	8.42±0.14	10.31±0.54	10.65±0.22^d,g^
	TNFα	6.96±0.14	5.15±0.21^b^	6.63±0.35	7.15±0.37^g^
Adhesion, growth, and angiogenes is factors	Agt	5.39±0.28	5.61±0.22	9.01±0.62^c^	10.14±0.67^g^
	CD31	3.10±0.21	2.48±0.09	4.53±0.35^a^	5.07±0.35^g^
	CD34	2.85±0.19	2.88±0.26	4.32±0.13^c^	4.65±0.25^g^
	Col1	11.01±0.50	9.57±0.29	9.66±0.44	8.38±0.15
	FN1	8.65±0.40	7.87±0.38	3.57±0.49^c^	2.86±0.13^g^
	HIF1a	5.12±0.13	4.76±0.13	4.48±0.15^a^	4.70±0.09
	IGF1	4.81±0.17	4.69±0.18	6.15±0.18^c^	6.38±0.05^g^
	IDO	11.78±0.18	13.33±0.42	15.09±0.73^a^	14.76±0.96
	MMP11	9.23±0.36	8.11±0.24^a^	9.23±0.20	8.67±0.21
	Pai-1	3.47±0.42	5.98±0.53^b^	6.72±0.34^c^	6.37±0.40
	αSMA	3.56±0.47	2.78±0.44	5.09±0.17^a^	5.11±0.23^f^
	TGFβ	5.59±0.19	4.91±0.11	4.94±0.22	5.19±0.19
	TnC	14.87±0.29	12.90±0.45^b^	9.71±0.38^c^	8.65±0.23^g^
	UCP1	14.02±0.23	13.98±0.54	15.92±0.32^b^	16.12±0.21^f^
	VCAM	4.24±0.12	4.82±0.13	5.15±0.31^a^	5.35±0.19
	VE-cadh	5.19±0.25	4.50±0.19	6.46±0.27^a^	6.64±0.27^g^
	VEGFa	5.24±0.33	5.13±0.14	5.25±0.46	4.71±0.36
	VEGFR2	6.33±0.14	5.13±0.23	6.78±0.40	7.01±0.37^f^
	VLDLR	3.41±0.10	2.98±0.06	6.71±0.39^c^	7.59±0.54^g^

a vs LFD p<0.05.

b vs LFD p<0.01.

c vs LFD p<0.001.

d vs LF FFL p<0.05.

e vs HFD p<0.05.

f vs HFD p<0.01.

g vs HFD p<0.001.

**Figure 6:**
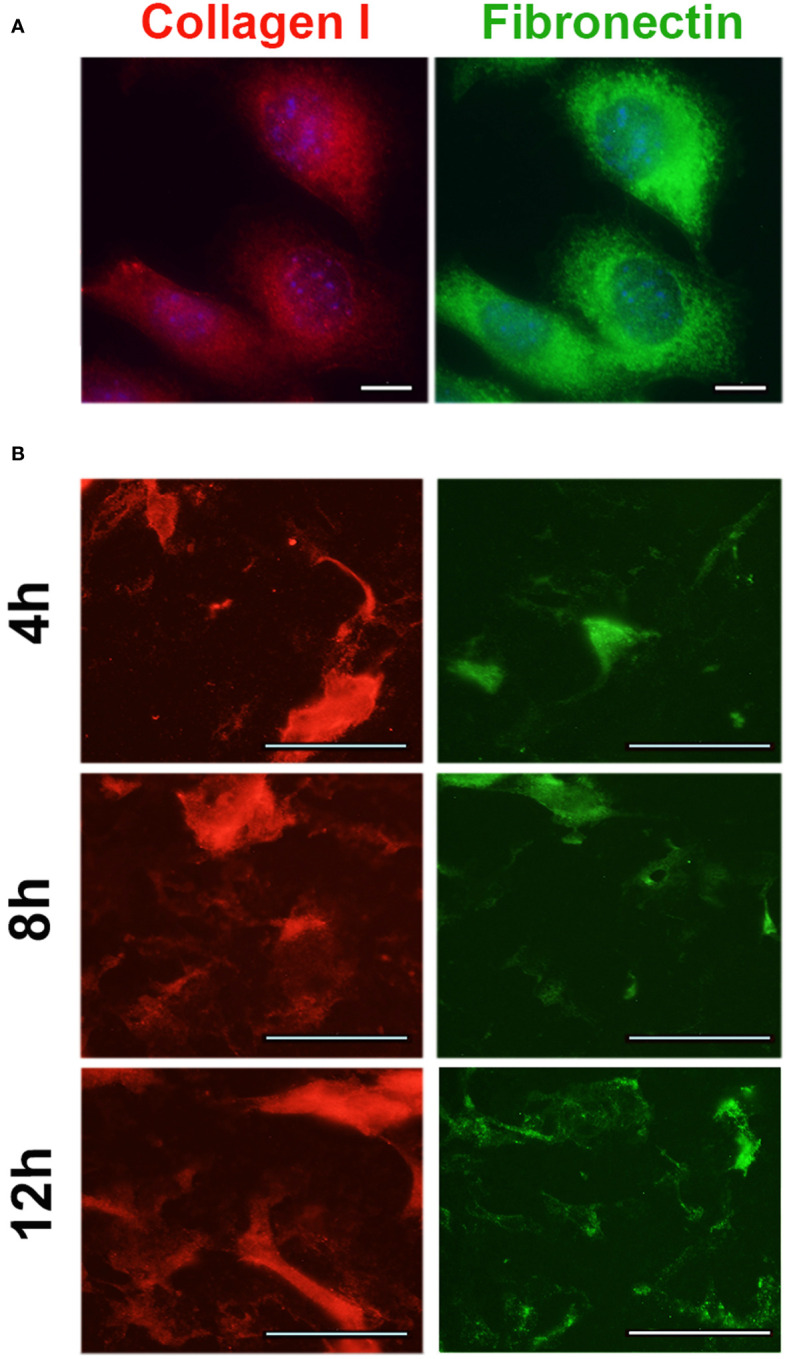
Expression of extracellular matrix proteins in MOSE-L_TIC_ *_v_* cells. **(A)** MOSE-L_TIC_ *_v_* spheroids were seeded on tissue culture plates, removed and the plates were stained for collagen 1 and fibronectin 1 secretion. **(B)** Expression of collagen 1 and fibronectin 1 in adherent MOSE-L_TIC_ *_v_* cells.

The expression of the key adipokines adiponectin, adipisin, leptin, resistin, and visfatin were significantly decreased by the cancer cells irrespective of the diet in the OFB (**Table 2**), but not in the other adipose tissues (**Supplementary Table S4**). The cancer cells alone increased several pro-inflammatory chemo- and cytokines (*Ccl2*, *Ccl4*, *Cxcr2*, *Il1b*, *Il10*) but decreased *Cxcl13*. Adhesion and invasion factors (*Col1*, *Fn1*, *Tnc*) were also upregulated while growth and angiogenesis regulators were downregulated. Interestingly, the MOSE-L_TIC_ *_v_* cells themselves express moderate to high levels of several pro-inflammatory cytokines and chemokines and their receptors and regulators (i.e., *Ccl2*, *Ccl7*, *Csf1*, *Cxcl5*, *Cebpb*, *Mif*) as well as extracellular matrix proteins and their regulators (i.e., *Fn1*, *Mmp25*, *S100A11*, *Spp1*) (**Supplementary Table S5**). MOSE-L_TIC_ *_v_* cells growing as monolayers express the extracellular matrix proteins Col1 and FN1 (**Figure 6A**) and readily secrete both after only 4 h of adhesion of MOSE-L_TIC_ *_v_* spheroids (**Figure 6B**). Thus, the cancer cells themselves contribute to a permissive microenvironment.

The combination of HFD plus cancer resulted in increased expression of some cytokines in the OFB (e.g., *Ccl2, Ccl4*, *Cxcr2, Il1b)* and decreased expression of others (e.g., *Ccr5*, *Cx3cl1*, *eNos*, *Tnfa*) as compared to the HFD group alone (**Table 2**). Genes that support adhesion and invasion (*Col1*, *FN1*, *Mmp11*, *TnC*) were also significantly higher in the HFD/MOSE-L_TIC_ *_v_* group while angiogenesis regulators had lower expression. These changes were not observed in the pmWAT or rpWAT (**Supplementary Table S4**).

**Figure 7:**
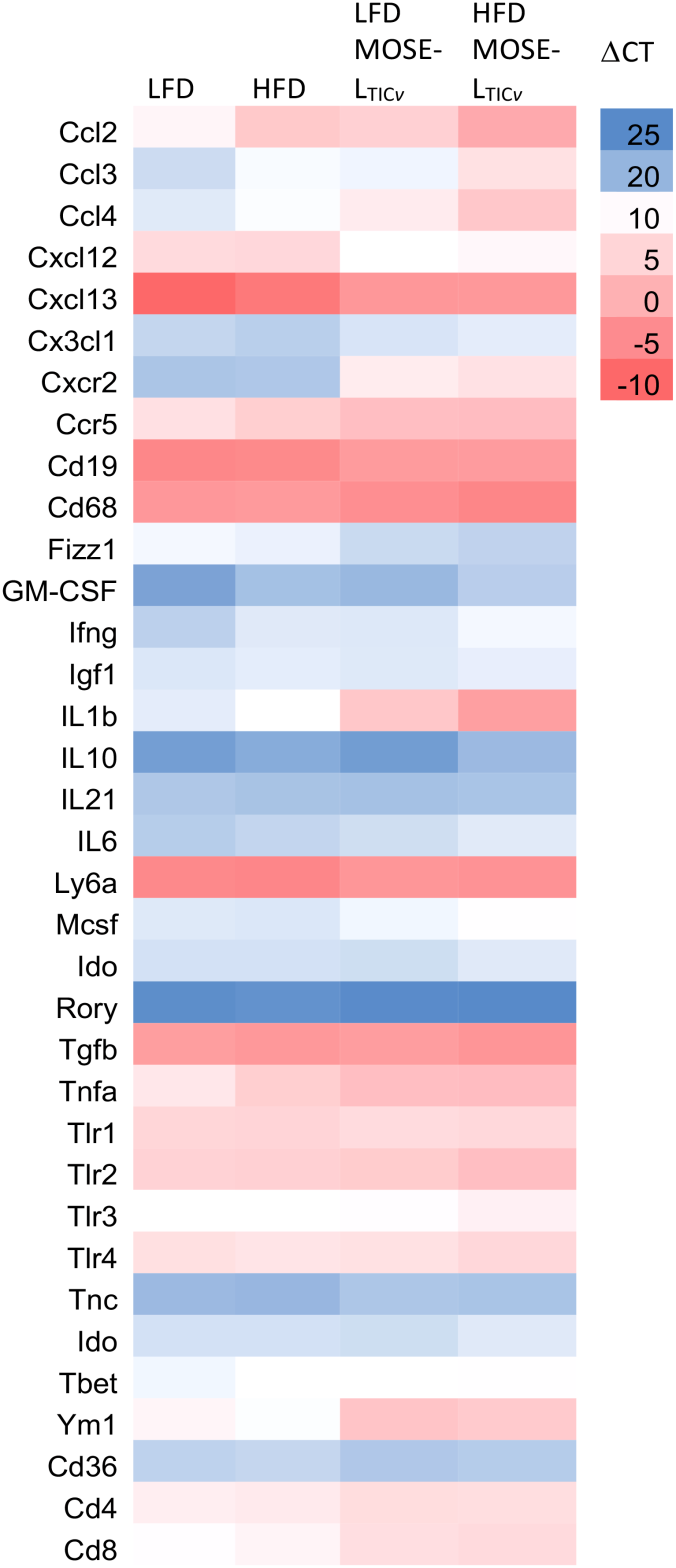
Heatmap of select gene expression changes (ΔCT values) in the cellular fraction in the peritoneal serous fluid (PSF). qRT-PCR was employed to determine gene expression levels in the PSF of mice fed the low-fat (LFD) or the high-fat diet (HFD) and injected with the MOSE-L_TIC_ *_v_* cancer cells.

The PSF plays an important role in transporting and seeding cancer cells along its flow pattern throughout the peritoneal cavity. Since we observed an increase in the number of non-adherent cancer cells in the PSF (see **Figure 4B**), we also investigated diet and cancer-mediated changes in gene expression in the cellular fraction of the PSF that may contribute to the survival of the disseminating cells. The cellular fraction of the PSF contained mostly CD45+ leukocytes (**Table 1**; **Supplementary Table S2A**) and all genes selected for analysis had lower expression levels than in the OFB and peritoneal WATs (see **Supplementary Table S1, S4**). The HFD alone induced a significant increase in the expression of several pro-inflammatory genes such as *Ccl2*, *Ccl3*, *Ccl4*, *Gmcsf*, *Ifng*, *Il1b*, and *Tnfa* (**Figure 7**; **Supplementary Table S6**); these genes are involved in monocyte/granulocyte recruitment and activation and their expression levels correlate with the increased recruitment into the PSF in the HFD group (**Supplementary Table S2A**). In contrast, pattern recognition receptors (*Tlr1-4*) or adhesion and migration regulators (*Tnc*) were not affected by the HFD. The presence of cancer cells alone resulted in higher expression of the same genes in the PSF that were upregulated by the HFD. In addition, genes involved in leukocyte recruitment (C*xcr2*) or promotion of metastasis (*Ccr5*) had higher expression after cancer cell implantation while genes that regulate B cell migration and chemotaxis (*Cxcl12*, *Cxcl13*) were downregulated; this correlates with the reduction of the B cell marker *Cd19* and confirms the reduction of B cells in the PSF in the HFD and the cancer group (**Supplementary Table S2A**). The combination of HFD and MOSE-L_TIC_ *_v_* led to an exacerbation of the expression of the pro-inflammatory cyto/chemokines (*Ccl2*, *Ccl3*, *Ccl4*, *Cxcl13*, *Ifng*, *Il1b, Il10)* over the LFD/MOSE-L_TIC_ *_v_* group; other genes were not further affected by this combination beyond the impact of the HFD or the cancer cells alone.

**Figure 8:**
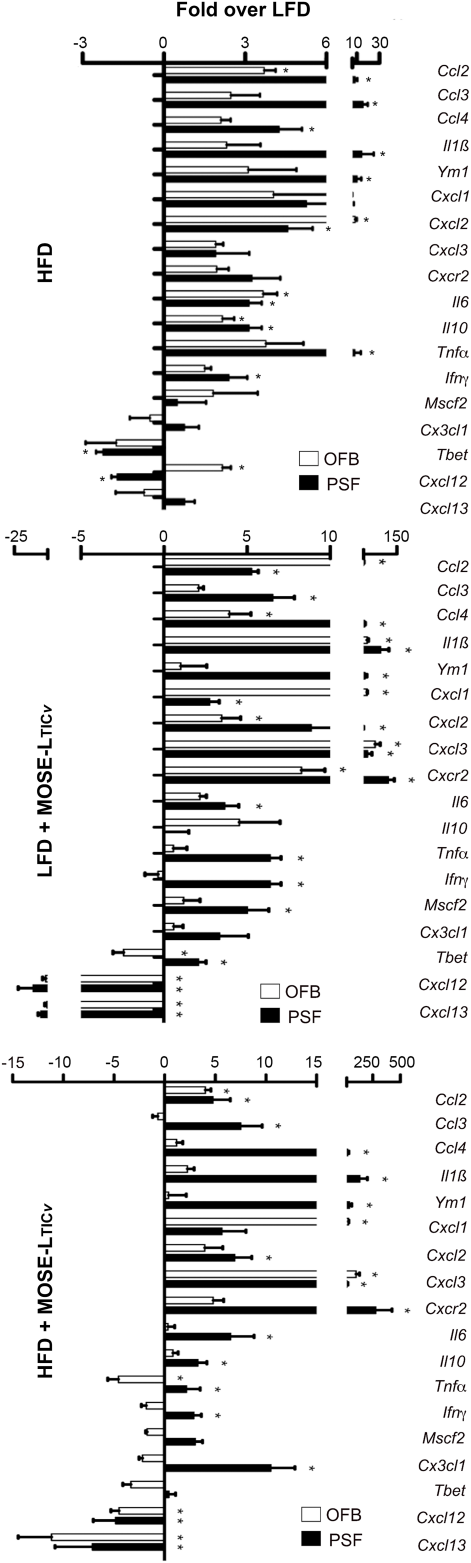
Comparison of changes in gene expression in the peritoneal serous fluid (PSF) and the omental fatband (OFB). Fold differences over the low-fat diet (LFD) in the expression of select genes in the cellular fraction of the PSF and the OFB (determined by qRT-PCR) from mice fed the high-fat diet (HFD) (top panel), the LFD group injected with MOSE-L_TIC_ *_v,_* (middle panel) and the high-fat diet (HFD) injected with MOSE-L_TIC_ *_v_*. *p<0.05 *vs* LFD.

A comparison of the gene expression profiles of the OFB as the first metastatic site and the PSF as a transport medium for the disseminating ovarian cancer cells shows a comparable response to the diet and cancer cells in both tissues; however, the response in the PSF was more pronounced for most genes and conditions. As shown in **Figure 8**, both the HFD and the cancer cells increased the expression of most genes; however, especially *Il1b*, *Ym1*, *Cxcl2*, *Cxcl3*, and *Cxcr2* were among the most responsive genes to both the HFD and the cancer cells in the PSF which were further exacerbated by the combination of the HFD and cancer cells. These gene expression patterns are consistent with the immune cell compositional changes observed in both the PSF and OFBs of cancer-bearing mice: elevated leukocyte populations and the decline in B cells by both the HFD and the cancer treatments. This is mirrored by a decrease in *Cxcl13*, a potent B cell chemoattractant which was reduced by the HFD but more so by the cancer cell presence. Also downregulated in both tissues was *Cxcl12*, an anti-inflammatory chemokine. We noted several genes that were differentially regulated in the tissues by the diet and cancer cells. In the OFB, *Tbet* (involved in NK cell maturation and function) is increased and *Cx3cl1*, *Ifng* and *Mscf2* are decreased by the HFD with little response to the cancer cells while increased in the PSF in both cancer groups.

## 4 Discussion

Obesity is associated with an increased risk of developing ovarian cancer and a lower likelihood to survive the disease. Higher mortality appears to be especially prominent when the onset of obesity is prior to diagnosis or before 18 years of age (28). A better understanding of the underlying mechanisms of this obesity-mediated risk increase is critical for the development and implementation of more effective prevention strategies to lower the cancer risk in these women. So far, the disruption of hormone regulation, changes in adipokine production, local and systemic inflammation, and increased energy supply have all been suggested to play a role in ovarian cancer development and progression (29). Inflamed mammary adipose tissues have also been associated with an increased risk of breast cancer (30), highlighting the importance of changes in adjacent adipose tissues for cancer development. We have shown previously that abdominal adipose tissues have a unique immune cell composition (15) and that changes in the CD45^+^ leukocyte populations observed in parous mice was correlated with a lower ovarian cancer tumor burden (16). In the present study we determined the overlap of HFD- and cancer-induced changes in tissues relevant for ovarian cancer development and progression to generate a risk “fingerprint” that links obesity to ovarian cancer risk. We used the MOSE model that recapitulates genetic changes in functional categories that are observed in the human disease progression rather than testing the interaction of obesity with cancer cells with specific gene mutations. We tested our hypothesis that a HFD promotes the generation of a permissive, pre-metastatic environment that supports cancer cell survival during peritoneal dissemination, and secondary outgrowth. We focused our investigation on cellular and molecular changes in the OFB as an early metastatic site, the PSF as transport medium for the disseminating ovarian metastases, blood as indicator of systemic effects and the pmWAT and rpWAT as non-visceral abdominal fat depot controls.

Feeding mice a HFD increased the size of all adipose tissues investigated but did not cause a net influx of immune cells. The response to the HFD was tissue dependent: we observed a net depletion of B cells and an increase in mono/granulocytes and CD5^-^ lymphoid cells in the PSF and the OFB with no effect on T cells while B cells were decreased and T cells were increased in the blood. The changes in immune populations in the OFB were accompanied by an increased expression of pro-inflammatory cyto- and chemokines and their receptors, survival-, growth-, and adhesion-promoting factors, and genes that are generally associated with a poor outcome similar to the changes induced by the cancer cells in mice fed a LFD. These results suggest that the HFD-induced cellular and molecular changes contribute to the generation of permissive conditions for ovarian metastases. Accordingly, we found a significantly increased peritoneal tumor burden in the mice fed the HFD.

The non-visceral abdominal adipose tissues pmWAT and rpWAT also responded to the HFD and the cancer cells with an increase in the number of resident immune cells but the changes in their composition was modest. While we observed overall increases in inflammatory gene expression levels due to the HFD that often mimicked those induced by MOSE-L_TIC_ *_v_*, albeit of a lower magnitude, these were less pronounced than the changes observed in the OFB and PSF. Genes that support adhesion were not elevated, and MOSE-L_TIC_ *_v_* lesions were rarely observed in these tissues. Thus, while not a preferred metastatic site, the non-visceral adipose tissues may add to ovarian cancer risk by contributing to the pro-inflammatory peritoneal environment as well as by functioning as an energy reservoir for adjacent tumors (11). Further, the abdominal adipose tissues contain progenitor and stem cells in the stromal vascular fraction that can be recruited to ovarian metastases (31). We have recently shown that incorporation of the stromal cells supported the survival, adhesion and invasion of non-adherence ovarian cancer cell aggregates via reduced respiration and proliferation, as well as elevated expression of genes that increase senescence, telomere maintenance, and DNA repair (22, 32). Whether the HFD changes the stem- and progenitor populations in the abdominal adipose tissues or affects their recruitment to disseminating ovarian metastases needs further investigation.

Both the cancer cells and the HFD led to the net depletion of B cells in the OFB and PSF. This was associated with the reduced expression of the key regulators of B cell chemotaxis, *Cxcl12* and *Cxcl13* by both the HFD and the cancer cells. The role of B cells in cancer progression appears to be multi-faceted since both poor (33) or better outcomes (34, 35) have been associated with B cell infiltration. B cell depletion can promote an immuno suppressive environment (36) while B cell infiltration contributes to an anti-cancer defense via stimulation of T cell responses (37, 38). B cell infiltration of tumors correlates with more favorable outcomes and lower relapse rates when associated with an influx of T cells (39). We have previously reported that parity established a tumor suppressing peritoneal environment that was characterized by increased proportions of B cells and a decrease in macrophages in the OFB and PSF compared to nulliparous mice (16). Further, Il-12 expression in the tumors was also associated with increased B cells and reduced macrophages in the PSF and OFB (14), suggesting that B cells localized in these tissues may contribute to the lower tumor burden observed in both studies. In addition to B cell depletion, we observed a significant shift in the accumulation of bone marrow precursor-derived B2 B cells over the peritoneal B1 B cells in the PSF with the combination of the HFD and the cancer cells, suggesting a reduction of the innate immune response in the PSF. Hence, our studies suggest that B cells are important players in the generation of permissive or refractory conditions in tissues critical for ovarian cancer dissemination and metastatic outgrowth.

Increased T cells were observed in both blood (contributed to the systemic inflammatory state) and PSF, but they were found significantly reduced in the OFB as a consequence of both the HFD and cancer cell presence. Interestingly, we did not observe significant changes in the CD4^+^ or CD8^+^ sub-populations, but a significant reduction in the percentage of DN T cells in both the PSF and the OFB was observed. DN T cells have been found to be higher in tumor tissue (40) and expand rapidly upon hypoxia (41). While the role of adipose tissue DN T cells in ovarian metastasis has not been delineated, DN T cells can exert a profound anti-tumor effect (42) due to their capacity to lyse tumor cells (43). Thus, the reduction of DN T cells in tissues critical for ovarian metastasis may also contribute to a permissive peritoneal environment for disseminating ovarian cancer cells.

Macrophages are the most abundant infiltrating leukocyte population in human ovarian tumors and ascites (44), enhancing tumor progression via immunosuppression and promotion of tumor growth, invasion, metastasis, and angiogenesis (45). The levels of resident macrophages in the PSF were significantly enhanced as a consequence of HFD but not by the cancer cells alone. It is possible that this is the result of recruitment to disseminating tumor cell aggregates, tumors or other sites since only single cell solutions were analyzed. Adipose-associated macrophages parallel tumor-associated macrophages in gene expression profiles which includes the enrichment of angiogenic factors, chemokines, cytokines, proteases, and growth factors that can be even higher in adipose tissue-derived macrophages than tumor-associated macrophages (46). Here, F4/80^lo^Ly6C^hi^ monocytes were increased in the PSF by the cancer cells. Ly6C^hi^ inflammatory monocytes are preferentially recruited to metastatic sites by Ccl2 (which is also highly expressed in the MOSE-L_TIC_ *_v_*) and allow for tumor cell extravasation and metastasis while residential monocytes (Ly6C^lo^) were found mostly in primary breast tumors (47). Thus, the loss of resident macrophages in the PSF could be due to the recruitment to cancer aggregates and combined with the increase of F4/80^lo^Ly6C^hi^ monocytes could aid metastasis to peritoneal organs. Further, macrophage populations were increased in the WATs with the HFD, which can induce M2 polarization (48), this could serve as an additional contribution of the adipose tissue to the induction of a metastasis-permissive microenvironment.

MDSCs are increased during inflammation and their recruitment to tumor sites has been associated with tumor promotion (49) and poor outcome (50) in part due to the suppression of innate and adaptive immunity. In our study we confirmed the increase in MDSCs in the blood of obese mice but also found elevated MDSC populations in the OFB. Importantly, the HFD significantly increased cancer-mediated MDSC recruitment to the PSF and OFB. The CD11B^hi^Ly6G^hi^, Ly6C^low^ MDSCs were categorized as the polymorphonuclear (PMN)-MDSC sub-population of a distinct phenotype that partially overlaps with the monocytic MDSCs (CD11B^+^Ly6G^-^Ly6C^hi^) (51). Activated PMN-MDSCs are poorly phagocytotic and their potent immunosuppressive activity is associated with high secretion of anti-inflammatory cytokines and reactive oxygen and nitrogen species (51), which can contribute to an immunosuppressive environment.

In early metastasis, disseminating cancer cells are transported throughout the peritoneal cavity by the PSF. During disease progression, the malignant ascites is accumulating which exposes the cells to low oxygen and nutrient levels as well as physical stresses which further enhances cancer development and metastatic potential (52). The malignant ascites contains varying levels of pro-inflammatory cyto- and chemokines, growth factors, bioactive lipids, and other factors that support chemotaxis, cancer proliferation, progression, lymphangiogenesis and angiogenesis (53), and thereby enables or supports many aspects of metastasis such as invasion through the mesothelial layer (54) and the development of a stem-like phenotype (55). Thus, the composition of the ascites is critical for the survival and progression of disseminating cancer cells. In the present study, no ascites was observed in mice on the HFD but obesity increased the number of viable cancer cells in the PSF. This was associated with changes in the immune cell population including the depletion of B cells as well as increases in resident macrophages and NK cells. While our analysis of gene expression changes in the cellular components of the PSF was limited, the fold increase in pro-inflammatory genes induced by the HFD alone was above levels found in the OFB and, importantly, the HFD further exacerbated cancer-induced increases in several of these genes. This was especially evident in genes such as *Il1b* [shown to be increased in malignant ascites in ovarian cancer patients (56)], *Ym1* (a marker for immunosuppressing M2 macrophages), and *Cxcr2* (expressed in inflammatory monocytes). Also increased by HFD but less so by the cancer cells was *Ccl2* in contrast to its receptor Ccr2 that was modestly increased by the HFD but highly upregulated in the cancer groups. *Ccl2* encodes a potent chemoattractant for monocytes and other immune cells and is a contributor to chronic inflammation. It is secreted by various cells in the tumor microenvironment such as fibroblasts, T cells, monocytes (correlating with the influx in monocytes into the PSF), endothelial cells, adipocytes, and tumor cells. The high levels of free fatty acids after ingestion of the HFD may contribute to the high levels of *Ccl2* via activation of TLR4 (57). *Ccl2* is also highly expressed in the MOSE-L_TIC_ *_v_* cells but is even higher in stromal cells derived from obese adipose tissues, and increased in hypoxic conditions (32). Thus, the HFD-mediated increase in *Ccl2* and its receptor promotes inflammation but also can enhance the autocrine CCL2-CCR2 axis that activates monocytes, macrophages, and NK cells, and promotes macrophage polarization. This axis also has been shown to stimulate tumor cell proliferation, migration, invasion, progression, and angiogenesis, and can promote chemoresistance in several cancers (58).

Additionally, both *Cxcl2* and *Cxcl3* expression were increased. CXCL2 can be expressed in neutrophils and tumor cells (also expressed in the MOSE-L_TIC_ *_v_*) and has been correlated with metastasis and lower survival (59). CXCL3 is a chemoattractant for neutrophils and has been shown to promote angiogenesis. Its expression in adipose cells can activate the focal adhesion signaling pathway in cancer cells leading to an enhanced mesenchymal phenotype, increased migration and invasion in breast cancer (60). In addition to inflammation, some of the elevated genes and their proteins may also contribute directly to the survival of metastases: *Cxc3cl1* via Akt activation (61), Tnfα by induction of Tgfβ secretion in mesothelial cells (62), Ym1 (encoding the murine chitinase-like protein3) via cell protection (63), and Il-10 via immunosuppression and promotion of proliferation and migration (64). These may be some of the mechanisms through which a HFD can contribute to a pro-inflammatory and pro-survival environment in the PSF that can support the survival of disseminating ovarian cancer cells.

**Figure 9:**
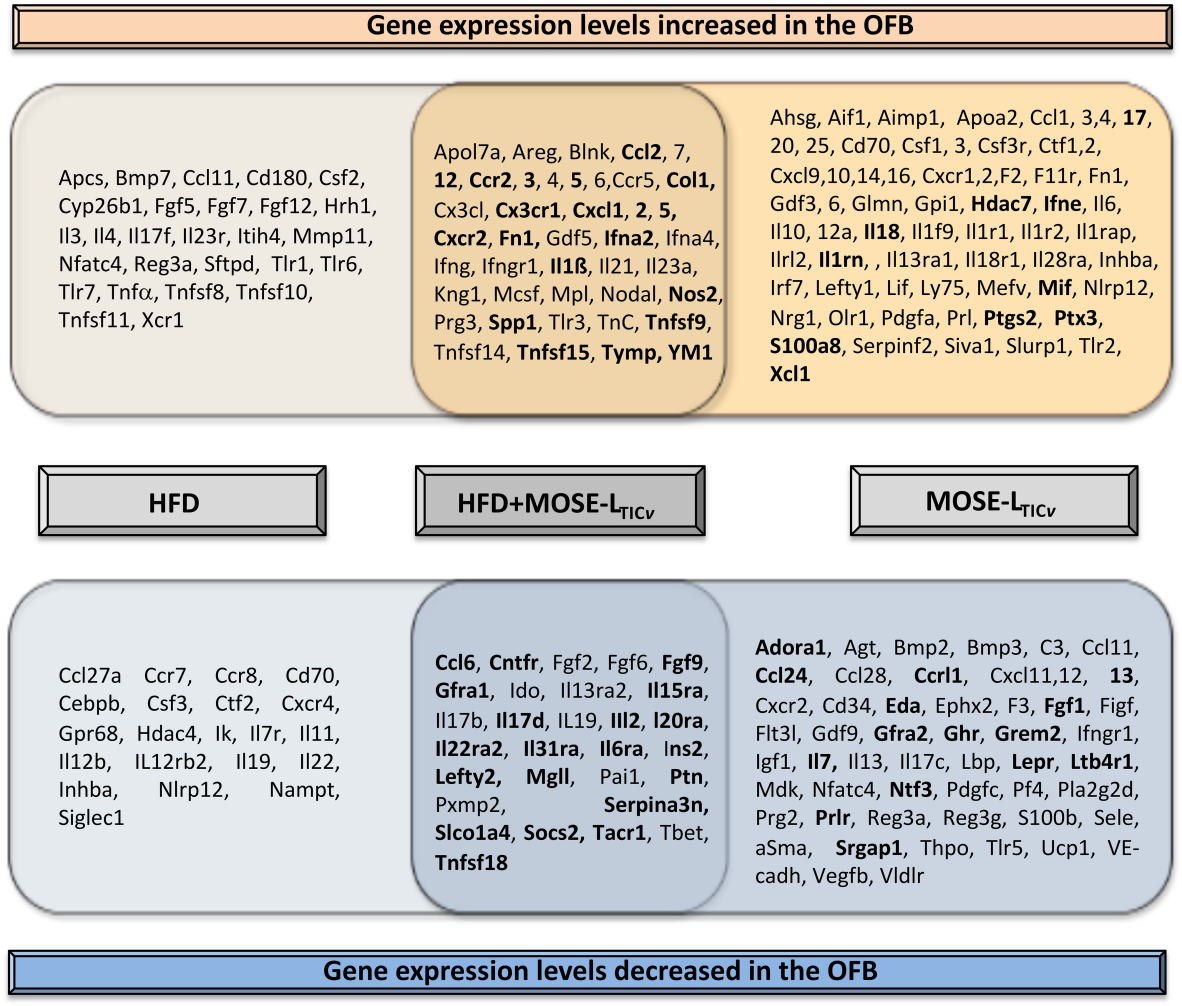
Overlap in gene expression changes between the high-fat diet (HFD) and the HFD plus cancer cell presence (MOSE-L_TIC_ *_v_*). Genes in bold indicate exacerbation of cancer-induced changes by the HFD.

One of the first adhesion sites is the OFB where cancer cells can be found in less than an hour after i.p. injection (16). The HFD alone generated similar inflammatory conditions in the OFB as observed in the PSF although the response was somewhat less pronounced. We observed a significant overlap of up- or downregulated genes by both the HFD and cancer presence (**Figure 9**). Upregulated were many cyto- and chemokines as well as regulators of growth and adhesion. Downregulated were genes that suppress cytokine synthesis, regulate apoptosis, and those often found suppressed in cancer. The HFD further exacerbated the cancer-mediated increase of several inflammatory genes and those associated with the regulation of growth, metabolism, and cancer progression while further reducing the expression of genes that inhibit cancer growth and development (**Figure 9**, in bold). Thus, these genes may represent a “permissive fingerprint” in the OFB generated by the HFD even before the arrival of cancer cells.

The HFD alone increased genes encoding matrix proteins such as COL1 and TNC in the OFB and exacerbated cancer-mediated increases in *Col1, Tnc*, and *Fn1*. These proteins have shown to increase adhesion, invasion, proliferation, and migration of ovarian metastases (65–68); FN1 has been also shown to be important marker for the transformation of the ovarian epithelial cells to a mesenchymal phenotype and the formation of aggregates (69, 70) while COL1 increased resistance to paclitaxel (71) and cisplatin (72) treatment. Both COL1 and FN1 have been detected in solid ovarian metastases but not in ascites cells, and it was thought that they originate from stromal cells rather than cancer cells (73). However, as shown in **Figure 6**, the MOSE-L_TIC_ *_v_* themselves generate and secrete both FN1 and COL1 even after brief adhesion. Other studies have found that tumor-associated macrophages secrete TNC and FN1and both proteins were among the most enriched in the macrophage secretome (74). TNC, FN1 and COL1 expression in in ascites or ovarian tumor tissue are associated with an unfavorable prognosis and a lower survival (74) (proteinatlas.org, accessed on 10/01/2023). The HFD-mediated increase in *Fn1* was more pronounced in the OFB than in other abdominal adipose tissues while *Tnc* and *Col1* expression levels were not affected in rpWT and pmWAT. While there is still discussion about the origin of several of the investigated genes [stromal versus immune versus tumor cells with considerable overlap (75)], our data suggest that the elevation of extracellular matrix genes specifically in the OFB can promote the adhesion of disseminating cancer cells to their first metastatic site and supports their metastatic outgrowth.

Another protein of interest is SPP1 (Osteopontin), a secreted matrix glycoproFtein that is expressed in areas of inflammation and tissue remodeling. SPP1 activates signaling cascades via binding to integrins or CD44; these pathways upregulate the expression of pro-survival genes, enhance proliferation, migration, progression, metastasis, angiogenesis (76) and adhesion via increasing CD44 and Integrin B1 expression (77). Receptor activation by SPP1 in immune cells affects cytokine production, immune cell differentiation and function, communication between the adaptive and innate immune system, and acts as an immune checkpoint to suppress the anti-cancer immune cell activity (78). *Spp1* was elevated by both the HFD and the cancer cells in the OFB; the HFD also elevated the cancer-mediated increase in *Spp1* expression in the OFB and, to a lesser extent, in the pmWAT and rpWAT. SPP1is already detectable in early ovarian cancer stages in serum (79). It is highly expressed in advanced ovarian cancer and has been associated with a poor prognosis and a low survival (19, 80). It is thought that SPP1 is mostly secreted by tumor cells (81) and, accordingly, we found high levels of *Spp1* in the MOSE-L_TIC_ *_v_* cells. However, the increased expression levels induced by the HFD in the OFB implicates *Spp1* as an important factor in the generation of a pro- inflammatory premetastatic niche; as a ligand of integrins and CD44 its presence in the extracellular matrix can also support cancer cell adhesion and subsequent metastatic outgrowth similar to COL1and FN1.

In summary, our data support the ‘*seed and soil*’ or *pre-metastatic niche* hypothesis that states that a conducive microenvironment must be formed in order for tumor cells to engraft and proliferate at secondary sites (82). However, we postulate that not only the primary tumor but also the HFD can contribute to the generation of permissive conditions that prepare the PSF and OFB for the arrival of the metastases, promoting their survival, adhesion, and invasion. Our results provide support for two mechanisms through which obesity can increase the ovarian cancer risk: first, by directly impacting the cellular and gene expression profile in critical tissues in the peritoneal cavity that together generate a pre-metastatic, permissive niche for disseminating ovarian cancer cells and second, by enhancing and sustaining cancer-mediated changes. In line with other reports (83) we observed a differential response of individual tissues with the pmWAT and rpWAT undergoing more limited changes but still contributing to a pro-inflammatory environment. These cellular and molecular profiles of a pro-metastatic microenvironment that mediate the relationship between obesity and cancer could be developed as effective targets for strategies to prevent or treat metastatic ovarian cancer.

## Data availability statement

The original contributions presented in the study are included in the article/**Supplementary Materials**, further inquiries can be directed to the corresponding author.

## Ethics statement

The animal study was approved by Institutional Animal Care and Use Committee at Virginia Tech. The study was conducted in accordance with the local legislation and institutional requirements.

## Author contributions

AS: Data curation, Formal analysis, Investigation, Methodology, Writing – original draft, Writing – review & editing. CL: Data curation, Methodology, Writing – review & editing. JG: Data curation, Methodology, Writing – review & editing. PR: Conceptualization, Formal analysis, Methodology, Supervision, Writing – review & editing. ES: Conceptualization, Formal analysis, Funding acquisition, Methodology, Project administration, Resources, Supervision, Writing – review & editing.
