# Supplementary figures and images for: Obesity modulates the cellular and molecular microenvironment in the peritoneal cavity: implication for ovarian cancer risk
Source: Front Immunol. 2024 Jan 9;14:1323399. doi: 10.3389/fimmu.2023.1323399 (PMC10803595; doi:10.3389/fimmu.2023.1323399)

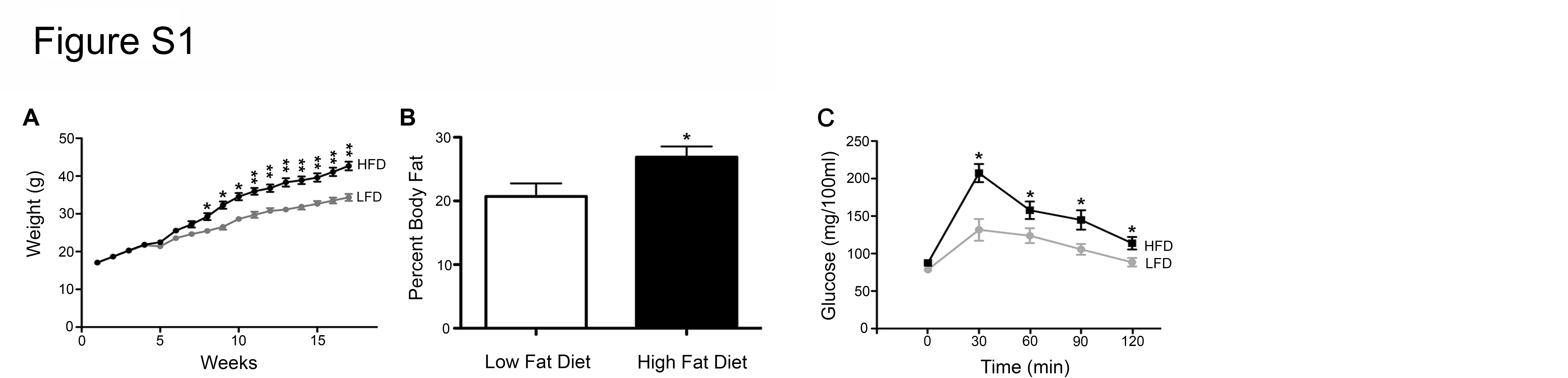

Supplement: Supplementary file 1 [file Image_1.tif]
