# Supplementary material for: Obesity modulates the cellular and molecular microenvironment in the peritoneal cavity: implication for ovarian cancer risk
Source: Front Immunol. 2024 Jan 9;14:1323399. doi: 10.3389/fimmu.2023.1323399 (PMC10803595; doi:10.3389/fimmu.2023.1323399)
Supplement: Supplementary file 2 [file Table_1.docx]

Supplemental Table S1: ΔCT values for the individual tissues from mice on a low-fat (LFD) or high-fat diet (HFD) and injected with PBS (controls) or MOSE-L_TIC_*_v_* cells (qPCR array)

| Gene symbol | Gene description | OFB LFD | OFB HFD | OFB LFD+ MOSE-L_TIC_*_v_* | OFB HFD+ MOSE-L_TIC_*_v_* | pmWAT LFD | pmWAT HFD | pmWAT LFD+ MOSE-L_TIC_*_v_* | pmWAT HFD+ MOSE-L_TIC_*_v_* | rpWAT LFD | rpWAT HFD | rpWAT LFD+ MOSE-L_TIC_*_v_* | rpWAT HFD+ MOSE-L_TIC_*_v_* |  |
| --- | --- | --- | --- | --- | --- | --- | --- | --- | --- | --- | --- | --- | --- | --- |
| Adora1 | Adenosine A1 receptor | 2.541 | 2.138 | 5.593 | 7.417 | 4.090 | 3.621 | 3.404 | 3.491 | 5.471 | 3.725 | 4.322 | 3.613 |  |
| Ahsg | Alpha-2-HS-glycoprotein | 5.048 | 5.380 | 3.535 | 7.095 | 5.835 | 6.419 | 6.150 | 6.931 | 9.226 | 5.543 | 3.524 | 6.394 |  |
| Aif1 | Allograft infl. factor 1 | -1.304 | -1.407 | -2.586 | -2.397 | 2.253 | 1.105 | 0.330 | -0.146 | 1.203 | -0.127 | -1.020 | -1.788 |  |
| Apcs | Serum amyloid P-component | 10.812 | 7.747 | 10.280 | 10.742 | 11.453 | 11.465 | 11.474 | 11.507 | 6.593 | 9.239 | 8.572 | 9.717 |  |
| Apoa2 | Apolipoprotein A-II | 4.315 | 4.578 | 1.543 | 3.729 | 4.780 | 5.591 | 4.082 | 4.785 | 1.964 | 2.020 | -0.443 | 3.428 |  |
| Apol7a | Apolipoprotein L 7a | 4.198 | 3.500 | 0.965 | 1.116 | 5.524 | 5.010 | 5.229 | 3.959 | 6.371 | 6.107 | 4.805 | 3.174 |  |
| Apol8 | Apolipoprotein L 8 | 7.907 | 7.565 | 7.784 | 6.999 | 11.453 | 11.465 | 10.024 | 11.507 | 9.226 | 9.239 | 9.317 | 9.717 |  |
| Areg | Amphiregulin | 10.81 | 9.124 | 4.416 | 3.672 | 10.707 | 8.933 | 8.963 | 6.859 | 8.907 | 7.464 | 6.513 | 3.962 |  |
| Bcl6 | B-cell leukemia/ lymphoma 6 | -0.533 | -0.126 | -0.252 | -0.013 | 1.613 | 1.732 | 1.694 | 2.101 | 1.751 | 1.150 | 0.942 | 0.352 |  |
| Blnk | B-cell linker | 1.108 | 0.541 | 0.556 | 0.080 | 4.046 | 2.730 | 2.737 | 2.984 | 3.423 | 3.253 | 2.869 | 1.060 |  |
| Bmp1 | Bone morphogenetic protein 1 | -0.923 | -1.545 | -1.377 | -1.919 | 0.672 | 0.705 | 0.443 | 0.841 | 1.583 | 1.515 | 1.439 | -0.341 |  |
| Bmp2 | Bone morphogenetic protein 2 | 3.425 | 3.013 | 4.916 | 5.458 | 6.835 | 6.659 | 6.733 | 6.458 | 8.450 | 9.231 | 8.162 | 6.354 |  |
| Bmp3 | Bone morphogenetic protein 3 | 0.250 | 0.184 | 3.578 | 3.820 | 0.031 | 0.329 | 0.030 | 0.739 | 2.661 | 1.843 | 1.647 | 1.045 |  |
| Bmp7 | Bone morphogenetic protein 7 | 3.064 | 1.634 | 3.943 | 3.986 | 5.674 | 5.983 | 7.320 | 5.949 | 5.332 | 4.028 | 4.975 | 5.139 |  |
| C3 | Complement component 3 | -5.980 | -6.059 | -4.983 | -4.358 | -1.821 | -2.098 | -2.627 | -2.007 | -1.192 | -1.444 | -2.095 | -2.088 |  |
| C3ar1 | Complement comp. 3a receptor 1 | -0.749 | -1.460 | -1.514 | -1.497 | 2.045 | -0.194 | 0.249 | -0.416 | 1.307 | -0.339 | -0.026 | -1.085 |  |
| Cast | Calpastatin | -2.436 | -2.028 | -1.917 | -1.721 | -0.302 | -0.375 | -0.401 | -0.545 | -0.059 | -0.479 | -0.750 | -0.766 |  |
| Ccl1 | Chemokine (C-C motif) ligand 1 | 8.181 | 8.081 | 5.392 | 5.210 | 11.453 | 9.668 | 7.894 | 6.356 | 9.082 | 9.239 | 5.608 | 6.360 |  |
| Ccl11 | Chemokine (C-C motif) ligand 11 | 0.009 | -1.412 | 3.213 | 2.570 | -0.554 | -0.842 | -0.409 | -0.580 | -1.982 | -0.081 | 0.166 | -1.629 |  |
| Ccl12 | Chemokine (C-C motif) ligand 12 | 2.162 | 0.866 | -0.842 | -1.804 | 3.239 | 2.463 | 2.436 | 0.519 | 2.764 | 1.921 | 1.193 | -1.185 |  |
| Ccl17 | Chemokine (C-C motif) ligand 17 | 4.259 | 5.066 | 3.094 | 1.848 | 7.415 | 5.291 | 5.091 | 3.748 | 3.915 | 4.179 | 3.210 | 2.916 |  |
| Ccl19 | Chemokine (C-C motif) ligand 19 | -0.973 | -0.739 | -0.673 | -0.114 | 3.231 | 2.831 | 2.950 | 2.947 | 1.792 | 1.929 | 1.680 | 1.323 |  |
| Ccl2 | Chemokine (C-C motif) ligand 2 | 2.117 | 0.870 | -1.993 | -2.394 | 2.974 | 0.925 | 1.187 | -0.361 | 1.586 | -1.140 | -0.553 | -1.764 |  |
| Ccl20 | Chemokine (C-C motif) ligand 20 | 10.812 | 10.685 | 7.742 | 7.732 | 11.453 | 11.465 | 11.474 | 10.028 | 9.226 | 9.239 | 9.317 | 9.717 |  |
| Ccl22 | Chemokine (C-C motif) ligand 22 | 2.484 | 2.806 | 2.219 | 2.730 | 6.897 | 5.635 | 5.933 | 4.079 | 5.950 | 4.316 | 4.841 | 4.164 |  |
| Ccl24 | Chemokine (C-C motif) ligand 24 | 2.680 | 2.340 | 4.592 | 5.912 | 5.863 | 5.194 | 5.416 | 6.482 | 6.405 | 5.352 | 6.430 | 5.091 |  |
| Ccl25 | Chemokine (C-C motif) ligand 25 | 5.599 | 5.013 | 2.711 | 2.059 | 5.518 | 5.793 | 4.784 | 4.733 | 5.239 | 6.643 | 5.766 | 4.031 |  |
| Ccl27a | Chemokine (C-C motif) ligand 27A | -0.015 | 1.018 | 0.119 | -0.128 | 1.770 | 2.549 | 1.491 | 2.163 | 1.375 | 1.311 | 0.578 | 1.002 |  |
| Ccl28 | Chemokine (C-C motif) ligand 28 | 5.736 | 4.972 | 6.769 | 6.712 | 6.377 | 7.163 | 7.017 | 6.597 | 6.974 | 6.739 | 7.188 | 6.034 |  |
| Ccl3 | Chemokine (C-C motif) ligand 3 | 3.886 | 3.850 | 1.595 | 1.575 | 4.870 | 4.861 | 4.482 | 3.666 | 4.171 | 3.084 | 3.264 | 1.933 |  |
| Ccl4 | Chemokine (C-C motif) ligand 4 | 3.964 | 3.4553 | 1.112 | 1.760 | 7.075 | 4.659 | 5.384 | 3.509 | 5.778 | 3.880 | 3.691 | 1.980 |  |
| Ccl5 | Chemokine (C-C motif) ligand 5 | -2.419 | -2.010 | -2.977 | -2.768 | 1.396 | 0.818 | 0.787 | -0.286 | 1.006 | -0.555 | -0.905 | -1.858 |  |
| Ccl6 | Chemokine (C-C motif) ligand 6 | -3.874 | -2.791 | -2.559 | -1.283 | -1.339 | -1.007 | -1.005 | -1.031 | -1.895 | -1.863 | -2.062 | -2.308 |  |
| Ccl7 | Chemokine (C-C motif) ligand 7 | 1.163 | -0.048 | -1.512 | -1.978 | 1.861 | -0.172 | 0.918 | -1.334 | 0.649 | -1.789 | -0.440 | -2.340 |  |
| Ccl8 | Chemokine (C-C motif) ligand 8 | -2.670 | -2.336 | -3.262 | -2.782 | -1.382 | -1.739 | -1.620 | -2.827 | -0.911 | -1.262 | -2.521 | -3.347 |  |
| Ccl9 | Chemokine (C-C motif) ligand 9 | -1.758 | -1.046 | -0.820 | -0.949 | 0.048 | 0.293 | 0.600 | 0.084 | -0.812 | -1.027 | -0.864 | -0.854 |  |
| Ccr1 | Chemokine (C-C motif) receptor 1 | -0.354 | -0.719 | -0.697 | -0.882 | 3.674 | 3.428 | 2.991 | 1.667 | 3.142 | 2.590 | 2.027 | 0.524 |  |
| Ccr10 | Chemokine (C-C motif) receptor 10 | 4.158 | 3.699 | 3.727 | 3.833 | 7.354 | 8.817 | 6.016 | 6.975 | 9.029 | 9.239 | 9.317 | 6.376 |  |
| Ccr2 | Chemokine (C-C motif) receptor 2 | -0.428 | -1.822 | -1.211 | -2.072 | 1.606 | 0.256 | 0.717 | -0.142 | 3.220 | 1.966 | 2.474 | -0.169 |  |
| Ccr3 | Chemokine (C-C motif) receptor 3 | 1.615 | -1.030 | 0.540 | -1.092 | 3.081 | 1.407 | 2.116 | 0.858 | 4.216 | 4.048 | 4.099 | 1.419 |  |
| Ccr4 | Chemokine (C-C motif) receptor 4 | 4.319 | 3.816 | 4.254 | 4.962 | 9.213 | 7.937 | 8.676 | 7.192 | 9.226 | 8.223 | 8.159 | 7.500 |  |
| Ccr5 | Chemokine (C-C motif) receptor 5 | 0.714 | -0.668 | -0.817 | -1.356 | 2.954 | 1.400 | 1.874 | 0.638 | 4.634 | 3.329 | 3.299 | 0.364 |  |
| Ccr6 | Chemokine (C-C motif) receptor 6 | 1.216 | 0.232 | 0.386 | 0.208 | 9.442 | 6.851 | 6.043 | 5.628 | 9.226 | 9.239 | 8.814 | 5.098 |  |
| Ccr7 | Chemokine (C-C motif) receptor 7 | 2.723 | 3.893 | 1.920 | 2.413 | 8.157 | 8.358 | 7.064 | 6.668 | 7.926 | 8.009 | 7.426 | 4.936 |  |
| Ccr8 | Chemokine (C-C motif) receptor 8 | 4.501 | 6.089 | 3.625 | 4.161 | 8.558 | 8.826 | 7.885 | 6.189 | 7.752 | 9.239 | 8.691 | 6.209 |  |
| Ccr9 | Chemokine (C-C motif) receptor 9 | 3.293 | 2.537 | 2.904 | 3.2173 | 7.765 | 7.262 | 5.953 | 6.627 | 9.226 | 8.595 | 8.185 | 6.333 |  |
| Ccrl1 | Chemokine (C-C motif) receptor-like 1 | 4.304 | 4.810 | 6.007 | 6.350 | 6.298 | 6.571 | 7.429 | 8.967 | 9.226 | 8.038 | 8.724 | 6.596 |  |
| Ccrl2 | Chemokine (C-C motif) receptor-like 2 | 1.602 | 1.249 | 1.955 | 2.171 | 4.075 | 2.772 | 3.663 | 2.942 | 4.159 | 3.530 | 3.434 | 2.209 |  |
| Cd14 | CD14 antigen | -2.410 | -1.808 | -1.847 | -1.586 | -0.044 | 0.099 | 0.024 | 0.046 | -0.521 | -0.474 | -1.256 | -0.876 |  |
| Cd180 | CD180 antigen | 0.616 | -0.377 | 0.265 | 0.203 | 5.131 | 2.781 | 3.439 | 2.835 | 4.950 | 3.110 | 4.187 | 1.334 |  |
| Cd27 | CD27 antigen | 2.733 | 2.563 | 2.811 | 3.024 | 7.336 | 7.153 | 6.949 | 6.164 | 9.226 | 8.082 | 7.399 | 5.450 |  |
| Cd28 | CD28 antigen | 1.442 | 1.686 | 1.491 | 1.865 | 5.609 | 4.588 | 5.094 | 4.500 | 6.642 | 5.890 | 4.944 | 3.287 |  |
| Cd4 | CD4 antigen | 1.431 | 1.534 | 1.178 | 1.458 | 6.214 | 5.155 | 4.629 | 4.376 | 7.201 | 7.322 | 4.870 | 3.992 |  |
| Cd40 | CD40 antigen | 0.566 | 1.257 | 0.713 | 1.265 | 4.724 | 4.707 | 4.672 | 4.010 | 3.873 | 3.040 | 3.565 | 2.073 |  |
| Cd40lg | CD40 ligand | 3.152 | 2.476 | 3.570 | 3.327 | 7.587 | 6.828 | 6.432 | 6.070 | 8.410 | 8.441 | 9.057 | 5.640 |  |
| Cd70 | CD70 antigen | 8.085 | 9.198 | 5.752 | 7.260 | 9.674 | 11.466 | 9.736 | 9.414 | 8.798 | 9.240 | 7.964 | 5.876 |  |
| Cd74 | CD74 antigen | -7.897 | -7.857 | -7.554 | -7.792 | -4.739 | -5.228 | -4.947 | -5.375 | -4.086 | -4.637 | -4.923 | -6.457 |  |
| Cd86 | CD86 antigen | -0.616 | -0.473 | -0.327 | 0.030 | 2.235 | 1.970 | 1.763 | 1.670 | 1.504 | 1.011 | 0.731 | 0.968 |  |
| Cd97 | CD97 antigen | -3.275 | -3.101 | -2.639 | -2.258 | -0.254 | -0.661 | 0.042 | -0.207 | 0.193 | -0.346 | -0.244 | -1.302 |  |
| Cebpb | CCAAT/enhancer binding protein beta | -3.955 | -2.486 | -4.515 | -4.548 | -2.354 | -1.272 | -2.046 | -2.178 | -3.362 | -3.056 | -3.769 | -3.559 |  |
| Cer1 | Cerberus 1 homolog | 10.812 | 10.685 | 10.822 | 9.064 | 11.453 | 11.465 | 11.474 | 11.507 | 9.226 | 9.239 | 9.317 | 9.717 |  |
| Cklf | Chemokine-like factor | 0.326 | 0.503 | -0.189 | 0.245 | 3.207 | 3.081 | 2.695 | 2.227 | 2.415 | 1.774 | 1.722 | 1.203 |  |
| Clcf1 | Cardiotrophin-like cytokine factor 1 | 1.231 | 0.607 | 0.326 | 0.738 | 7.098 | 6.067 | 5.306 | 4.664 | 9.226 | 6.340 | 6.814 | 4.970 |  |
| Cmtm1 | CKLF-like MARVEL transm. domain cont. 1 | 10.812 | 10.328 | 10.822 | 9.929 | 10.463 | 11.056 | 11.137 | 10.649 | 9.226 | 9.239 | 9.317 | 9.638 |  |
| Cmtm2a | CKLF-like MARVEL transm domain cont. 2A | 10.812 | 10.685 | 10.822 | 10.742 | 11.453 | 11.465 | 8.990 | 9.349 | 8.191 | 9.239 | 9.317 | 8.188 |  |
| Cntfr | Ciliary neurotrophic factor receptor | 3.365 | 4.276 | 5.049 | 6.318 | 7.749 | 9.07 | 6.908 | 7.236 | 6.474 | 6.202 | 5.824 | 5.497 |  |
| Crp | C-reactive protein, pentraxin-related | 7.198 | 6.784 | 6.526 | 8.988 | 7.896 | 8.111 | 8.473 | 9.294 | 5.948 | 7.694 | 4.646 | 7.833 |  |
| Csf1 | Colony stimulating factor 1 (macrophage) | -1.304 | -1.178 | -2.382 | -2.439 | 1.790 | 2.151 | 1.320 | 1.082 | 3.021 | 2.601 | 1.310 | -0.095 |  |
| Csf2 | Colony stimulating factor 2 | 6.970 | 4.819 | 6.598 | 5.685 | 10.732 | 7.229 | 9.404 | 8.145 | 9.226 | 6.919 | 7.656 | 6.640 |  |
| Csf2ra | Colony stimulating factor 2 receptor, alpha, low-affinity | -0.218 | 0.390 | 0.321 | 1.325 | 3.863 | 3.928 | 3.034 | 3.673 | 4.789 | 3.397 | 3.433 | 3.321 |  |
| Csf3 | Colony stimulating factor 3 (granulocyte) | 8.367 | 9.374 | 6.627 | 6.347 | 11.453 | 9.767 | 8.720 | 10.189 | 9.226 | 9.239 | 9.317 | 8.990 |  |
| Csf3r | Colony stim. factor 3 receptor | 1.915 | 1.798 | 0.168 | 0.607 | 6.318 | 4.810 | 3.617 | 3.895 | 5.758 | 4.847 | 3.871 | 3.967 |  |
| Ctf1 | Cardiotrophin 1 | 2.947 | 3.600 | 1.698 | 2.694 | 4.605 | 5.682 | 4.086 | 4.888 | 7.528 | 7.331 | 4.669 | 4.653 |  |
| Ctf2 | Cardiotrophin 2 | 7.280 | 8.424 | 5.380 | 5.567 | 9.634 | 11.465 | 9.984 | 9.915 | 9.226 | 9.239 | 9.317 | 9.717 |  |
| Cx3cl1 | Chemokine (C-X3-C motif) ligand 1 | 1.336 | 1.263 | 1.014 | 0.468 | 4.362 | 4.338 | 4.062 | 3.841 | 4.351 | 3.091 | 2.656 | 2.411 |  |
| Cx3cr1 | Chemokine (C-X3-C) receptor 1 | 3.167 | 2.015 | 0.659 | 0.215 | 7.930 | 7.078 | 4.025 | 3.858 | 8.898 | 8.924 | 4.038 | 3.663 |  |
| Cxcl1 | Chemokine (C-X-C motif) ligand 1 | 8.550 | 6.838 | 2.908 | 1.525 | 7.414 | 7.062 | 5.317 | 4.898 | 9.226 | 7.710 | 6.077 | 3.562 |  |
| Cxcl10 | Chemokine (C-X-C motif) ligand 10 | 0.754 | 0.385 | -1.557 | -1.787 | 4.064 | 2.848 | 1.781 | 0.319 | 3.997 | 1.606 | 0.772 | -1.190 |  |
| Cxcl11 | Chemokine (C-X-C motif) ligand 11 | 4.382 | 3.848 | 5.806 | 6.075 | 6.286 | 5.845 | 4.845 | 5.361 | 6.939 | 6.434 | 5.482 | 4.275 |  |
| Cxcl12 | Chemokine (C-X-C motif) ligand 12 | -2.471 | -2.484 | -1.688 | -1.327 | 0.582 | 0.378 | -0.085 | 0.382 | 1.722 | 0.631 | 0.706 | 0.294 |  |
| Cxcl13 | Chemokine (C-X-C motif) ligand 13 | -6.394 | -6.329 | -5.941 | -4.515 | -1.563 | -0.333 | -1.557 | 0.826 | -2.540 | -1.070 | -3.150 | -0.599 |  |
| Cxcl14 | Chemokine (C-X-C motif) ligand 14 | 3.437 | 3.527 | 1.073 | 0.961 | 2.257 | 3.000 | 2.821 | 1.966 | 1.183 | 0.112 | 0.630 | 0.345 |  |
| Cxcl15 | Chemokine (C-X-C motif) ligand 15 | 10.812 | 10.685 | 10.822 | 9.120 | 11.453 | 11.465 | 11.474 | 9.768 | 9.226 | 9.239 | 9.317 | 9.394 |  |
| Cxcl16 | Chemokine (C-X-C motif) ligand 16 | -1.173 | -1.038 | -2.255 | -2.567 | 0.834 | 0.398 | 0.399 | -0.199 | 2.304 | 1.136 | 0.579 | -0.786 |  |
| Cxcl2 | Chemokine (C-X-C motif) ligand 2 | 4.439 | 3.127 | 1.688 | 0.631 | 4.264 | 4.370 | 4.260 | 4.004 | 3.921 | 4.474 | 4.044 | 2.299 |  |
| Cxcl5 | Chemokine (C-X-C motif) ligand 5 | 5.438 | 3.580 | -2.367 | -3.457 | 11.453 | 9.406 | 0.915 | 0.382 | 8.646 | 7.744 | 0.883 | -1.135 |  |
| Cxcl9 | Chemokine (C-X-C motif) ligand 9 | -0.879 | -0.511 | -2.214 | -2.472 | -0.034 | 0.201 | -0.619 | -1.599 | 0.603 | -0.790 | -0.773 | -2.613 |  |
| Cxcr3 | Chemokine (C-X-C motif) receptor 3 | 0.426 | 0.208 | 0.217 | -0.297 | 3.833 | 3.156 | 3.607 | 2.924 | 3.497 | 2.370 | 2.226 | 1.055 |  |
| Cxcr4 | Chemokine (C-X-C motif) receptor 4 | -2.253 | -1.033 | -2.451 | -2.154 | 1.134 | 2.151 | 0.966 | 1.178 | 1.205 | 2.110 | 0.617 | -0.315 |  |
| Cxcr5 | Chemokine (C-X-C motif) receptor 5 | 0.061 | 0.226 | 0.592 | 1.498 | 10.121 | 6.771 | 6.838 | 7.931 | 8.602 | 9.189 | 7.533 | 6.700 |  |
| Cxcr6 | Chemokine (C-X-C motif) receptor 6 | 0.513 | 0.304 | 0.945 | 1.164 | 4.284 | 3.725 | 3.605 | 2.945 | 4.579 | 3.908 | 3.679 | 2.094 |  |
| Cybb | Cytochrome b-245, beta polypeptide | -1.548 | -1.318 | -1.589 | -1.265 | 2.751 | 1.474 | 1.687 | 1.223 | 2.521 | 1.387 | 0.829 | 0.213 |  |
| Cyp26b1 | Cytochrome P450, family 26, subfamily b, polypeptide 1 | 5.738 | 4.790 | 6.413 | 6.314 | 10.289 | 9.743 | 11.278 | 10.026 | 9.226 | 9.239 | 8.469 | 9.495 |  |
| D17Wsu104e | DNA segment, Chr 17, Wayne State University 104 | -1.731 | -1.617 | -2.641 | -2.574 | -0.029 | -0.614 | -0.623 | -0.762 | 0.383 | -0.554 | -0.786 | -1.726 |  |
| Dock2 | Dedicator of cyto-kinesis 2 | -0.816 | -0.676 | -0.451 | 0.198 | 3.801 | 3.035 | 2.765 | 2.820 | 3.381 | 2.623 | 2.492 | 2.206 |  |
| Ebi3 | Epstein-Barr virus induced gene 3 | 0.709 | 0.958 | 0.613 | 1.184 | 3.709 | 3.031 | 3.027 | 3.261 | 3.899 | 2.830 | 2.019 | 1.889 |  |
| Eda | Ectodysplasin-A | 2.256 | 3.146 | 4.676 | 5.625 | 3.951 | 4.067 | 4.277 | 4.857 | 2.950 | 2.632 | 2.490 | 2.482 |  |
| Ephx2 | Epoxide hydrolase 2, cytoplasmic | -0.858 | -1.203 | 2.402 | 1.961 | -1.680 | -2.028 | -2.167 | -2.330 | -1.347 | -1.316 | -1.527 | -2.891 |  |
| Epo | Erythropoietin | 10.812 | 10.686 | 10.822 | 10.742 | 11.453 | 11.465 | 11.474 | 11.507 | 9.226 | 9.239 | 9.317 | 9.717 |  |
| Epor | Erythropoietin receptor | 4.379 | 3.866 | 3.793 | 4.157 | 5.876 | 5.316 | 5.195 | 5.156 | 8.007 | 7.717 | 7.041 | 5.682 |  |
| Erbb2 | V-erb-b2 erythroblastic leukemia viral oncogene homolog 2 | 2.987 | 2.804 | 2.777 | 2.893 | 6.038 | 7.898 | 6.347 | 6.058 | 6.427 | 6.210 | 6.311 | 4.931 |  |
| Erbb2ip | Erbb2 interacting protein | -1.321 | -0.929 | -2.068 | -1.748 | 1.630 | 1.280 | 0.951 | 0.821 | 1.334 | 0.672 | 0.450 | 0.121 |  |
| F11r | F11 receptor | -0.689 | -0.408 | -2.564 | -2.284 | 2.070 | 1.988 | 1.164 | 1.096 | 3.086 | 2.540 | 0.979 | 0.477 |  |
| F2 | Coagulation factor II | 6.909 | 6.948 | 5.567 | 8.159 | 11.453 | 11.465 | 11.474 | 8.944 | 7.486 | 8.375 | 5.546 | 7.727 |  |
| F3 | Coagulation factor III | 0.214 | 0.030 | 1.476 | 1.308 | 1.011 | 0.479 | 1.021 | 0.862 | 0.831 | -0.670 | -0.127 | -0.506 |  |
| F8 | Coagulation factor VIII | 2.932 | 3.290 | 3.086 | 3.310 | 3.656 | 3.839 | 3.267 | 3.839 | 2.868 | 2.856 | 2.524 | 2.722 |  |
| Fasl | Fas ligand (TNF superfamily, member 6) | 4.930 | 4.849 | 4.935 | 4.420 | 9.067 | 6.547 | 7.994 | 6.461 | 7.933 | 7.570 | 8.306 | 5.114 |  |
| Fgf1 | Fibroblast growth factor 1 | -2.973 | -2.449 | -1.224 | -0.358 | -0.779 | -1.162 | -0.775 | -0.489 | -1.544 | -1.481 | -1.297 | -1.106 |  |
| Fgf10 | Fibroblast growth factor 10 | 0.950 | 0.196 | 0.712 | -0.160 | 1.374 | 0.801 | 1.252 | 1.038 | 3.513 | 2.44 | 2.832 | 1.155 |  |
| Fgf12 | Fibroblast growth factor 12 | 10.812 | 7.649 | 10.822 | 8.237 | 8.8801 | 9.562 | 9.839 | 8.230 | 9.226 | 6.367 | 9.317 | 5.985 |  |
| Fgf2 | Fibroblast growth factor 2 | -0.267 | 0.398 | 0.276 | 0.176 | 2.3670 | 2.367 | 2.066 | 1.865 | 3.211 | 2.796 | 2.163 | 2.033 |  |
| Fgf3 | Fibroblast growth factor 3 | 10.812 | 10.685 | 10.822 | 10.742 | 11.453 | 10.055 | 11.474 | 10.349 | 9.226 | 9.239 | 9.317 | 9.717 |  |
| Fgf4 | Fibroblast growth factor 4 | 10.812 | 10.685 | 10.823 | 10.742 | 11.452 | 11.465 | 11.474 | 11.507 | 9.226 | 9.239 | 9.317 | 9.717 |  |
| Fgf5 | Fibroblast growth factor 5 | 10.812 | 8.585 | 10.823 | 10.742 | 9.726 | 10.845 | 10.043 | 11.310 | 9.226 | 9.239 | 9.317 | 9.717 |  |
| Fgf6 | Fibroblast growth factor 6 | 8.713 | 10.685 | 10.823 | 10.742 | 11.453 | 11.465 | 11.474 | 11.507 | 9.226 | 9.239 | 9.317 | 9.717 |  |
| Fgf7 | Fibroblast growth factor 7 | 3.500 | 2.015 | 4.455 | 3.963 | 3.956 | 3.554 | 4.177 | 3.832 | 5.631 | 4.668 | 6.219 | 4.076 |  |
| Fgf8 | Fibroblast growth factor 8 | 10.812 | 10.685 | 10.822 | 10.742 | 11.453 | 11.465 | 10.994 | 11.507 | 9.226 | 9.239 | 9.317 | 9.717 |  |
| Fgf9 | Fibroblast growth factor 9 | 3.352 | 4.386 | 5.465 | 6.368 | 3.882 | 3.950 | 4.387 | 5.064 | 4.758 | 4.330 | 5.169 | 3.675 |  |
| Figf | C-fos induced growth factor | -1.646 | -1.217 | -0.470 | -0.467 | -0.771 | -0.219 | -0.017 | 0.186 | -0.441 | 0.130 | -0.248 | -0.667 |  |
| Flt3l | FMS-like tyrosine kinase 3 ligand | -0.286 | -0.556 | 0.824 | 1.141 | 3.692 | 3.193 | 2.869 | 3.797 | 4.161 | 3.849 | 3.006 | 2.725 |  |
| Fn1 | Fibronectin 1 | -3.878 | -3.935 | -6.666 | -6.535 | -0.295 | -0.219 | -2.030 | -2.699 | -0.103 | 0.035 | -2.501 | -3.345 |  |
| Fos | FBJ osteosarc. oncogene | 0.105 | 0.974 | 0.179 | -0.739 | -0.117 | 1.033 | 0.772 | -0.220 | 1.571 | 2.756 | 1.504 | -0.009 |  |
| Fpr1 | Formyl peptide receptor 1 | 1.067 | 1.158 | 0.144 | 2.463 | 5.057 | 5.117 | 3.075 | 5.1687 | 5.471 | 6.008 | 2.313 | 3.731 |  |
| Gdf1 | Growth different. factor 1 | 5.285 | 4.663 | 5.421 | 7.253 | 9.411 | 9.409 | 6.912 | 7.874 | 9.226 | 9.239 | 9.317 | 9.717 |  |
| Gdf2 | Growth different. factor 2 | 10.812 | 10.685 | 10.822 | 9.582 | 11.453 | 9.746 | 11.474 | 9.677 | 9.226 | 9.239 | 9.317 | 9.717 |  |
| Gdf3 | Growth different. factor 3 | 5.172 | 5.471 | 3.491 | 3.599 | 10.148 | 6.203 | 6.677 | 5.792 | 7.891 | 6.496 | 7.222 | 4.409 |  |
| Gdf5 | Growth different. factor 5 | 7.668 | 6.705 | 6.390 | 7.744 | 5.641 | 6.296 | 5.818 | 6.931 | 8.507 | 6.808 | 6.728 | 5.095 |  |
| Gdf6 | Growth different. factor 6 | 6.621 | 6.593 | 3.254 | 3.988 | 11.453 | 11.465 | 10.611 | 7.608 | 8.460 | 9.239 | 7.183 | 7.278 |  |
| Gdf7 | Growth different. factor 7 | 6.217 | 5.862 | 6.537 | 7.562 | 7.189 | 6.848 | 6.730 | 7.640 | 9.226 | 9.239 | 8.247 | 7.357 |  |
| Gdf9 | Growth different. factor 9 | 3.806 | 3.837 | 5.143 | 4.645 | 5.154 | 5.441 | 4.986 | 5.341 | 5.379 | 4.153 | 4.718 | 3.927 |  |
| Gfra1 | Glial cell line derived neurotrophic factor family receptor alpha 1 | 4.386 | 5.277 | 5.428 | 7.006 | 6.948 | 6.650 | 6.189 | 6.804 | 6.405 | 9.239 | 6.582 | 5.820 |  |
| Gfra2 | Glial cell line derived neurotrophic factor family receptor alpha 2 | 1.085 | 0.855 | 2.361 | 3.078 | 3.279 | 2.910 | 2.942 | 3.132 | 2.558 | 2.2220 | 2.463 | 2.273 |  |
| Ghr | Growth hormone receptor | -4.569 | -4.362 | -2.605 | -2.354 | -4.318 | -4.580 | -4.170 | -3.744 | -1.366 | -5.527 | -5.494 | -5.475 |  |
| Glmn | Glomulin, FKBP associated protein | 1.199 | 0.938 | 0.361 | 0.369 | 2.751 | 2.994 | 2.416 | 2.352 | 2.675 | 2.091 | 1.701 | 1.470 |  |
| Gpi1 | Glucose phosphate isomerase 1 | -3.457 | -3.296 | -4.598 | -4.773 | -2.051 | -2.293 | -2.055 | -2.150 | -1.609 | -2.271 | -2.750 | -3.446 |  |
| Gpr68 | G protein-coupled receptor 68 | 3.212 | 4.533 | 2.661 | 3.286 | 6.999 | 7.566 | 7.084 | 6.779 | 9.226 | 7.802 | 7.606 | 6.992 |  |
| Grem1 | Gremlin 1 | 4.540 | 3.874 | 4.250 | 3.639 | 11.453 | 8.102 | 8.873 | 8.700 | 9.226 | 9.239 | 9.317 | 8.491 |  |
| Grem2 | Gremlin 2 homolog, cysteine knot superfamily (Xenopus laevis) | 1.662 | 1.563 | 3.922 | 5.186 | 4.586 | 4.328 | 4.479 | 5.784 | 6.783 | 8.356 | 6.297 | 6.522 |  |
| Grn | Granulin | -3.537 | -3.353 | -3.541 | -3.156 | -1.336 | -1.764 | -1.742 | -1.578 | -1.505 | -2.37 | -2.450 | -2.834 |  |
| Hdac4 | Histone deacetylase 4 | -0.176 | 1.200 | -0.191 | 0.356 | 2.199 | 3.291 | 2.435 | 2.680 | 3.324 | 2.661 | 2.400 | 1.530 |  |
| Hdac5 | Histone deacetylase 5 | 0.419 | 0.645 | 1.194 | 1.904 | 3.417 | 3.478 | 3.368 | 4.037 | 4.755 | 4.616 | 3.911 | 3.259 |  |
| Hdac7 | Histone deacetylase 7 | -1.351 | -1.512 | -2.518 | -3.017 | 0.631 | 0.634 | 0.376 | 0.696 | 1.335 | 0.791 | -0.008 | -0.948 |  |
| Hdac9 | Histone deacetylase 9 | 1.353 | 0.527 | 2.039 | 1.000 | 5.619 | 4.104 | 4.687 | 3.901 | 8.005 | 5.897 | 6.205 | 4.061 |  |
| Hrh1 | Histamine receptor H1 | 4.576 | 3.602 | 5.143 | 5.470 | 7.937 | 7.296 | 8.084 | 6.925 | 9.226 | 8.986 | 7.842 | 6.971 |  |
| Ifna11 | Interferon alpha 11 | 10.812 | 10.685 | 10.237 | 9.460 | 11.167 | 11.465 | 11.474 | 11.507 | 9.226 | 9.239 | 9.317 | 9.717 |  |
| Ifna14 | Interferon, alpha 14 | 10.812 | 10.685 | 10.821 | 7.424 | 11.453 | 11.465 | 11.474 | 11.127 | 9.226 | 9.239 | 9.317 | 9.717 |  |
| Ifna2 | Interferon alpha 2 | 10.812 | 9.315 | 9.180 | 8.218 | 11.453 | 10.069 | 11.474 | 9.498 | 9.226 | 9.239 | 9.317 | 9.717 |  |
| Ifna4 | Interferon alpha 4 | 10.812 | 9.826 | 8.587 | 9.173 | 10.241 | 11.192 | 10.414 | 11.050 | 9.226 | 9.239 | 8.747 | 8.339 |  |
| Ifna9 | Interferon alpha 9 | 10.812 | 10.685 | 10.821 | 8.832 | 11.453 | 11.465 | 11.474 | 11.507 | 9.226 | 9.239 | 9.317 | 9.717 |  |
| Ifnab | Interferon alpha B | 10.812 | 10.685 | 10.821 | 7.278 | 11.453 | 11.465 | 11.474 | 11.507 | 9.226 | 9.239 | 9.317 | 9.717 |  |
| Ifnar1 | Interferon (alpha and beta) receptor 1 | -1.238 | -1.033 | -1.717 | -1.680 | 1.581 | 0.842 | 0.965 | 0.810 | 1.997 | 1.134 | 0.810 | -0.350 |  |
| Ifnar2 | Interferon (alpha and beta) receptor 2 | -2.531 | -1.734 | -2.396 | -2.070 | -1.023 | -1.246 | -1.359 | -1.307 | -1.686 | -2.412 | -2.581 | -2.780 |  |
| Ifnb1 | Interferon beta 1, fibroblast | 10.788 | 10.685 | 10.822 | 9.895 | 11.453 | 11.466 | 11.474 | 11.507 | 9.226 | 9.239 | 9.317 | 9.717 |  |
| Ifne | Interferon epsilon | 10.812 | 10.685 | 8.237 | 6.914 | 8.887 | 11.466 | 8.962 | 11.507 | 9.226 | 9.239 | 8.703 | 8.865 |  |
| Ifng | Interferon gamma | 5.598 | 4.571 | 4.477 | 4.315 | 8.692 | 7.414 | 7.017 | 6.836 | 8.633 | 8.625 | 6.739 | 5.366 |  |
| Ifngr1 | Interferon gamma receptor 1 | -3.568 | -2.750 | -2.496 | -2.387 | -1.432 | -1.569 | -0.712 | -0.707 | -0.897 | -0.786 | -1.781 | -2.531 |  |
| Ifngr2 | Interferon gamma receptor 2 | -1.325 | -1.306 | -1.899 | -1.969 | 1.048 | 0.939 | 0.822 | 0.181 | 2.467 | 1.181 | 0.995 | -0.501 |  |
| Ifnk | Interferon kappa | 3.555 | 3.454 | 4.241 | 5.050 | 6.912 | 7.137 | 7.066 | 6.495 | 6.029 | 6.604 | 6.534 | 5.352 |  |
| Ik | IK cytokine | 0.243 | 3.520 | -0.101 | 2.071 | 2.397 | 4.100 | 3.769 | 4.275 | 3.359 | 2.412 | 2.423 | 1.782 |  |
| Il10 | Interleukin 10 | 3.041 | 2.252 | 1.175 | 1.562 | 5.864 | 4.441 | 5.176 | 4.440 | 4.803 | 3.500 | 3.773 | 2.442 |  |
| Il10ra | Interleukin 10 receptor, alpha | 0.155 | 0.237 | -0.273 | 0.147 | 3.642 | 4.028 | 3.490 | 2.878 | 4.331 | 3.347 | 2.617 | 1.568 |  |
| Il10rb | Interleukin 10 receptor, beta | -2.596 | -2.313 | -2.427 | -2.422 | 0.080 | -0.380 | -0.263 | -0.470 | -1.645 | -0.915 | -1.249 | -1.664 |  |
| Il11 | Interleukin 11 | 5.742 | 7.545 | 6.276 | 6.637 | 9.598 | 9.203 | 9.780 | 9.723 | 8.144 | 9.239 | 9.317 | 9.717 |  |
| Il11ra1 | Interleukin 11 receptor, alpha chain 1 | -1.387 | -1.161 | -1.052 | -1.395 | 1.039 | 1.035 | 0.741 | 0.924 | 1.543 | 1.204 | 0.596 | 0.683 |  |
| Il12a | Interleukin 12A | 4.571 | 4.145 | 3.599 | 4.334 | 11.453 | 9.386 | 8.327 | 7.497 | 9.226 | 8.966 | 6.908 | 7.449 |  |
| Il12b | Interleukin 12B | 3.656 | 4.677 | 2.803 | 2.755 | 6.707 | 7.813 | 7.106 | 4.777 | 7.370 | 9.239 | 5.714 | 4.167 |  |
| Il12rb1 | Interleukin 12 receptor, beta 1 | 7.945 | 7.993 | 7.688 | 6.970 | 11.453 | 11.465 | 11.474 | 11.507 | 9.226 | 9.239 | 9.317 | 9.717 |  |
| Il12rb2 | Interleukin 12 receptor, beta 2 | 6.703 | 8.569 | 7.434 | 9.222 | 11.453 | 11.465 | 10.706 | 10.051 | 9.226 | 9.239 | 9.317 | 9.717 |  |
| Il13 | Interleukin 13 | 7.214 | 7.941 | 9.581 | 7.889 | 11.453 | 9.439 | 10.092 | 10.620 | 8.614 | 8.448 | 8.151 | 9.717 |  |
| Il13ra1 | Interleukin 13 receptor, alpha 1 | 0.483 | 0.867 | -0.678 | -0.910 | 3.075 | 3.184 | 2.005 | 1.981 | 2.403 | 1.500 | 0.906 | 0.358 |  |
| Il13ra2 | Interleukin 13 receptor, alpha 2 | 7.024 | 8.608 | 9.272 | 7.682 | 4.201 | 3.946 | 6.124 | 4.394 | 3.706 | 3.229 | 5.267 | 3.362 |  |
| Il15 | Interleukin 15 | 0.465 | 0.595 | 1.052 | 1.076 | 2.896 | 2.557 | 2.367 | 2.213 | 2.771 | 1.706 | 1.597 | 1.268 |  |
| Il15ra | Interleukin 15 receptor, alpha | 0.417 | 1.407 | 2.228 | 3.023 | 3.292 | 4.165 | 3.072 | 3.629 | 3.163 | 3.665 | 2.784 | 2.348 |  |
| Il16 | Interleukin 16 | -0.713 | -0.597 | -0.815 | -0.586 | 4.570 | 4.170 | 3.422 | 3.338 | 6.713 | 5.024 | 4.913 | 3.244 |  |
| Il17a | Interleukin 17A | 10.812 | 10.685 | 10.822 | 10.742 | 11.453 | 11.465 | 11.474 | 11.507 | 9.226 | 9.239 | 9.317 | 9.717 |  |
| Il17b | Interleukin 17B | 6.586 | 7.588 | 8.579 | 6.386 | 7.444 | 8.615 | 9.162 | 10.035 | 8.538 | 6.929 | 9.317 | 7.298 |  |
| Il17c | Interleukin 17C | 6.784 | 6.650 | 8.251 | 8.057 | 11.453 | 9.610 | 11.474 | 9.808 | 9.226 | 9.239 | 9.317 | 9.717 |  |
| Il17d | Interleukin 17D | 5.201 | 6.126 | 7.679 | 8.098 | 6.885 | 7.739 | 6.767 | 7.274 | 5.591 | 8.698 | 8.952 | 7.173 |  |
| Il17f | Interleukin 17F | 9.075 | 6.838 | 9.231 | 7.442 | 11.453 | 6.964 | 11.179 | 8.088 | 8.205 | 8.952 | 8.707 | 7.405 |  |
| Il17ra | Interleukin 17 receptor A | 0.674 | 0.801 | 0.571 | 1.091 | 4.811 | 5.074 | 4.188 | 4.045 | 4.062 | 4.134 | 3.903 | 3.621 |  |
| Il17rb | Interleukin 17 receptor B | 3.736 | 4.374 | 4.710 | 5.920 | 5.687 | 5.730 | 5.340 | 5.685 | 6.847 | 5.119 | 5.080 | 4.825 |  |
| Il18 | Interleukin 18 | -0.312 | -0.326 | -1.995 | -2.304 | 1.653 | 1.683 | 1.005 | 0.526 | 1.067 | 0.632 | -0.204 | -0.140 |  |
| Il18r1 | Interleukin 18 receptor 1 | 2.193 | 1.390 | -0.492 | -0.941 | 6.078 | 5.772 | 4.653 | 3.783 | 9.226 | 9.239 | 5.540 | 3.816 |  |
| Il18rap | Interleukin 18 receptor accessory protein | 2.901 | 2.826 | 2.124 | 1.541 | 7.374 | 6.259 | 5.288 | 3.663 | 9.226 | 7.580 | 6.546 | 4.082 |  |
| Il19 | Interleukin 19 | 7.673 | 10.685 | 8.503 | 7.390 | 11.453 | 11.465 | 10.252 | 11.507 | 9.226 | 9.239 | 9.317 | 9.717 |  |
| Il1a | Interleukin 1 alpha | 4.735 | 4.017 | 4.103 | 3.366 | 11.453 | 9.297 | 9.652 | 6.389 | 8.117 | 9.059 | 9.317 | 6.455 |  |
| Il1b | Interleukin 1 beta | 5.059 | 3.047 | 1.174 | -0.018 | 5.890 | 6.574 | 3.715 | 3.638 | 5.185 | 6.383 | 4.580 | 3.102 |  |
| Il1f10 | Interleukin 1 family, member 10 | 10.812 | 10.685 | 10.822 | 10.742 | 11.453 | 11.465 | 11.474 | 11.507 | 9.226 | 9.239 | 9.317 | 9.717 |  |
| Il1f5 | Interleukin 1 family, member 5 (delta) | 10.812 | 10.685 | 10.822 | 10.742 | 11.453 | 11.465 | 11.474 | 11.507 | 9.226 | 9.239 | 9.317 | 9.717 |  |
| Il1f6 | Interleukin 1 family, member 6 | 10.039 | 10.685 | 10.822 | 10.742 | 10.140 | 11.416 | 11.474 | 11.507 | 9.226 | 9.239 | 9.317 | 9.717 |  |
| Il1f8 | Interleukin 1 family, member 8 | 10.812 | 10.536 | 10.822 | 10.742 | 9.946 | 11.465 | 11.474 | 11.507 | 9.226 | 9.239 | 9.317 | 9.717 |  |
| Il1f9 | Interleukin 1 family, member 9 | 6.914 | 7.583 | 4.264 | 3.931 | 11.453 | 11.465 | 6.058 | 7.712 | 8.997 | 9.239 | 6.421 | 6.918 |  |
| Il1r1 | Interleukin 1 receptor, type I | -1.267 | -0.857 | -2.213 | -2.394 | 1.095 | 1.473 | 0.924 | 0.439 | 0.658 | 0.461 | -0.470 | -0.743 |  |
| Il1r2 | Interleukin 1 receptor, type II | 4.064 | 4.242 | 0.294 | 0.357 | 4.074 | 4.789 | 3.838 | 3.186 | 1.526 | 2.495 | 1.922 | 2.029 |  |
| Il1rap | Interleukin 1 receptor accessory protein | 1.970 | 2.161 | 0.798 | 1.016 | 5.414 | 4.962 | 3.778 | 3.725 | 5.769 | 3.875 | 3.973 | 3.226 |  |
| Il1rapl2 | Interleukin 1 receptor accessory protein-like 2 | 10.812 | 10.685 | 10.822 | 10.742 | 11.453 | 11.465 | 11.474 | 11.507 | 9.226 | 9.239 | 9.317 | 9.717 |  |
| Il1rl1 | Interleukin 1 receptor-like 1 | 1.612 | 1.470 | 2.485 | 3.203 | 3.833 | 4.165 | 3.459 | 3.980 | 4.382 | 4.194 | 3.632 | 3.011 |  |
| Il1rl2 | Interleukin 1 receptor-like 2 | 4.712 | 4.926 | 2.926 | 2.981 | 7.418 | 7.376 | 6.851 | 7.104 | 9.226 | 8.206 | 8.315 | 7.073 |  |
| Il1rn | Interleukin 1 receptor antagonist | 6.381 | 6.627 | 2.814 | 1.937 | 8.370 | 4.155 | 6.340 | 3.862 | 5.303 | 3.934 | 6.136 | 2.711 |  |
| Il2 | Interleukin 2 | 4.440 | 5.445 | 5.336 | 6.040 | 7.461 | 7.120 | 7.114 | 6.626 | 9.226 | 5.843 | 6.613 | 6.495 |  |
| Il20 | Interleukin 20 | 10.812 | 10.685 | 10.822 | 10.015 | 11.453 | 11.465 | 11.474 | 11.507 | 9.226 | 9.239 | 9.317 | 9.716 |  |
| Il20ra | Interleukin 20 receptor, alpha | 7.460 | 9.458 | 8.552 | 10.742 | 11.453 | 11.465 | 10.278 | 11.481 | 9.226 | 9.239 | 8.681 | 9.717 |  |
| Il21 | Interleukin 21 | 10.812 | 6.580 | 7.015 | 6.522 | 11.453 | 9.845 | 10.269 | 10.792 | 9.226 | 9.239 | 9.317 | 7.730 |  |
| Il21r | Interleukin 21 receptor | 1.988 | 1.190 | 1.425 | 1.508 | 8.921 | 8.975 | 5.074 | 5.779 | 9.226 | 9.239 | 6.252 | 5.066 |  |
| Il22 | Interleukin 22 | 9.616 | 10.685 | 9.637 | 9.427 | 11.453 | 11.465 | 11.474 | 11.507 | 9.226 | 9.239 | 9.317 | 9.717 |  |
| Il22ra1 | Interleukin 22 receptor, alpha 1 | 1.927 | 1.919 | 2.630 | 3.104 | 7.401 | 6.336 | 6.375 | 6.047 | 9.226 | 9.239 | 7.318 | 6.648 |  |
| Il22ra2 | Interleukin 22 receptor, alpha 2 | 3.284 | 5.161 | 7.169 | 8.160 | 5.177 | 5.176 | 4.758 | 6.313 | 4.994 | 5.684 | 5.242 | 4.243 |  |
| Il23a | Interleukin 23, alpha subunit p19 | 10.812 | 8.887 | 6.430 | 7.549 | 11.453 | 10.671 | 9.406 | 9.238 | 9.226 | 9.239 | 9.317 | 9.717 |  |
| Il23r | Interleukin 23 receptor | 4.321 | 3.366 | 4.911 | 5.051 | 9.176 | 8.048 | 7.990 | 8.435 | 9.226 | 8.629 | 9.317 | 8.472 |  |
| Il24 | Interleukin 24 | 4.316 | 4.357 | 3.719 | 4.314 | 11.453 | 10.679 | 10.346 | 10.820 | 9.226 | 9.239 | 9.317 | 8.940 |  |
| Il27 | Interleukin 27 | 4.798 | 4.865 | 4.3067 | 5.082 | 8.135 | 8.742 | 7.098 | 6.651 | 7.424 | 8.650 | 7.218 | 6.703 |  |
| Il28ra | Interleukin 28 receptor alpha | 3.961 | 4.170 | 2.772 | 3.276 | 8.853 | 8.596 | 6.631 | 6.727 | 9.226 | 8.424 | 8.407 | 7.620 |  |
| Il2ra | Interleukin 2 receptor, alpha chain | 4.457 | 4.444 | 5.211 | 5.177 | 6.887 | 6.938 | 6.835 | 6.205 | 6.749 | 5.415 | 5.800 | 5.300 |  |
| Il2rb | Interleukin 2 receptor, beta chain | 1.109 | 1.336 | 1.218 | 1.685 | 6.581 | 7.364 | 5.675 | 4.950 | 7.358 | 7.995 | 6.227 | 4.539 |  |
| Il2rg | Interleukin 2 receptor, gamma chain | -1.690 | -1.857 | -1.839 | -1.680 | 2.148 | 1.408 | 1.221 | 0.720 | 2.168 | 1.599 | 0.550 | -0.284 |  |
| Il3 | Interleukin 3 | 10.812 | 9.655 | 10.822 | 10.742 | 11.453 | 11.465 | 11.474 | 11.507 | 9.226 | 9.239 | 9.100 | 9.660 |  |
| Il31 | Interleukin 31 | 10.812 | 10.685 | 10.822 | 9.122 | 11.453 | 11.465 | 11.474 | 11.507 | 9.226 | 9.239 | 9.317 | 9.717 |  |
| Il31ra | Interleukin 31 receptor A | 1.636 | 2.969 | 4.330 | 5.165 | 3.256 | 3.985 | 3.238 | 4.668 | 3.774 | 5.572 | 3.728 | 4.709 |  |
| Il3ra | Interleukin 3 receptor, alpha chain | 4.251 | 4.525 | 4.943 | 6.837 | 8.582 | 8.302 | 7.301 | 7.893 | 9.226 | 9.239 | 8.307 | 6.895 |  |
| Il4 | Interleukin 4 | 7.922 | 6.619 | 8.720 | 7.476 | 9.748 | 9.249 | 11.474 | 9.254 | 9.226 | 9.239 | 8.936 | 8.395 |  |
| Il4ra | Interleukin 4 receptor, alpha | 2.052 | 2.668 | 1.665 | 1.613 | 4.820 | 5.101 | 4.560 | 4.352 | 4.420 | 4.332 | 4.001 | 2.847 |  |
| Il5 | Interleukin 5 | 5.255 | 5.086 | 6.048 | 5.844 | 7.395 | 9.560 | 8.041 | 8.107 | 8.409 | 9.239 | 9.187 | 6.399 |  |
| Il5ra | Interleukin 5 receptor, alpha | 1.313 | 1.679 | 1.975 | 2.931 | 6.081 | 6.649 | 6.277 | 7.062 | 6.370 | 7.929 | 7.312 | 6.340 |  |
| Il6 | Interleukin 6 | 5.724 | 5.079 | 4.402 | 4.288 | 7.308 | 6.583 | 6.410 | 5.116 | 9.226 | 6.660 | 7.697 | 6.014 |  |
| Il6ra | Interleukin 6 receptor, alpha | -1.246 | -0.067 | 0.015 | 0.621 | 1.678 | 2.174 | 1.392 | 2.214 | 1.644 | 1.827 | 0.903 | 0.858 |  |
| Il6st | Interleukin 6 signal transducer | -3.484 | -3.541 | -3.255 | -3.393 | -1.122 | -0.902 | -1.542 | -1.249 | -0.661 | -0.91 | -1.751 | -2.157 |  |
| Il7 | Interleukin 7 | 2.713 | 3.575 | 4.466 | 5.412 | 6.213 | 6.064 | 7.013 | 6.645 | 3.864 | 3.990 | 4.732 | 4.275 |  |
| Il7r | Interleukin 7 receptor | -1.280 | -0.213 | -0.585 | 0.168 | 3.325 | 2.328 | 3.109 | 2.462 | 2.748 | 1.670 | 2.637 | 1.408 |  |
| Cxcr1 | Chemokine (C-X-C motif) receptor 1 | 8.951 | 9.271 | 7.784 | 7.058 | 11.453 | 10.658 | 9.042 | 10.050 | 9.226 | 9.239 | 9.317 | 9.717 |  |
| Cxcr2 | Chemokine (C-X-C motif) receptor 2 | 5.014 | 4.351 | 1.827 | 1.279 | 8.867 | 8.828 | 5.444 | 4.716 | 9.226 | 9.239 | 6.180 | 5.312 |  |
| Il9 | Interleukin 9 | 10.812 | 10.685 | 10.822 | 10.742 | 11.453 | 11.465 | 11.474 | 11.507 | 9.226 | 9.239 | 9.316 | 9.717 |  |
| Il9r | Interleukin 9 receptor | 4.780 | 3.933 | 5.115 | 4.442 | 11.453 | 11.465 | 10.000 | 9.166 | 9.226 | 9.239 | 9.317 | 9.717 |  |
| Inha | Inhibin alpha | 3.651 | 4.178 | 4.577 | 4.761 | 7.552 | 7.770 | 6.678 | 8.820 | 9.226 | 9.001 | 6.406 | 8.015 |  |
| Inhba | Inhibin beta-A | 2.153 | 3.033 | 1.081 | 1.266 | 6.142 | 6.237 | 5.243 | 4.766 | 9.226 | 4.950 | 4.284 | 3.048 |  |
| Inhbb | Inhibin beta-B | 1.566 | 1.432 | 2.404 | 3.352 | 4.815 | 4.684 | 3.876 | 4.105 | 6.210 | 5.063 | 4.658 | 3.707 |  |
| Ins1 | Insulin I | 10.812 | 10.686 | 10.822 | 8.131 | 11.453 | 11.465 | 11.474 | 11.507 | 9.226 | 9.239 | 9.317 | 9.717 |  |
| Ins2 | Insulin II | 2.326 | 4.082 | 5.843 | 6.985 | 10.180 | 11.465 | 9.901 | 9.294 | 9.226 | 8.185 | 9.011 | 9.213 |  |
| Irf4 | Interferon regulatory factor 4 | 0.121 | 0.413 | 0.980 | 1.521 | 2.801 | 4.308 | 2.906 | 5.111 | 3.800 | 5.343 | 2.876 | 3.970 |  |
| Irf7 | Interferon regulatory factor 7 | -0.658 | -0.015 | -1.735 | -1.334 | 3.093 | 2.762 | 1.809 | 1.492 | 3.401 | 3.205 | 1.715 | 0.800 |  |
| Itgb2 | Integrin beta 2 | -2.726 | -2.539 | -3.022 | -2.957 | 0.354 | -0.603 | 0.373 | -0.338 | -1.483 | -2.318 | -1.932 | -2.715 |  |
| Itih4 | Inter alpha-trypsin inhibitor, 4 | 5.195 | 3.067 | 5.268 | 5.628 | 6.148 | 6.900 | 7.351 | 7.117 | 8.362 | 6.861 | 4.396 | 6.472 |  |
| Kitl | Kit ligand | -1.283 | -1.479 | -1.332 | -1.546 | 0.707 | 0.384 | 0.335 | 0.186 | 1.692 | 1.628 | 0.978 | 0.071 |  |
| Kng1 | Kininogen 1 | 10.812 | 9.508 | 6.691 | 10.742 | 9.945 | 8.480 | 8.090 | 9.623 | 9.226 | 8.938 | 5.898 | 9.717 |  |
| Lbp | Lipopolysaccharide binding protein | -0.390 | 0.213 | 1.383 | 2.042 | -0.101 | -0.162 | 0.086 | -0.182 | -0.283 | -1.398 | -0.766 | -1.683 |  |
| Lefty1 | Left right determ. factor 1 | 1.689 | 1.685 | -0.195 | -0.489 | 4.079 | 4.356 | 3.624 | 2.978 | 3.350 | 2.755 | 1.824 | 1.398 |  |
| Lefty2 | Left-right determ. factor 2 | 7.724 | 9.072 | 10.822 | 9.240 | 11.453 | 8.313 | 11.474 | 9.756 | 9.226 | 9.239 | 9.317 | 9.717 |  |
| Lepr | Leptin receptor | -0.586 | -0.047 | 1.125 | 1.977 | 3.217 | 4.395 | 2.475 | 4.874 | 4.805 | 3.915 | 3.760 | 3.916 |  |
| Lif | Leukemia inhibitory factor | 5.249 | 5.538 | 2.404 | 2.031 | 7.638 | 7.118 | 5.780 | 5.432 | 6.239 | 5.741 | 5.093 | 3.253 |  |
| Lifr | Leukemia inhibitory factor receptor | -1.878 | -1.621 | -2.410 | -2.279 | 1.443 | 1.740 | 1.036 | 0.857 | 2.421 | 2.330 | 1.329 | 0.445 |  |
| Lta | Lymphotoxin A | 3.580 | 2.896 | 3.774 | 3.621 | 11.453 | 9.324 | 8.921 | 7.721 | 9.226 | 8.748 | 8.568 | 6.030 |  |
| Ltb | Lymphotoxin B | -0.953 | -1.710 | -1.571 | -1.379 | 5.784 | 5.690 | 4.744 | 4.157 | 7.029 | 6.673 | 6.022 | 2.658 |  |
| Ltb4r1 | Leukotriene B4 receptor 1 | 6.207 | 6.470 | 7.212 | 8.397 | 9.842 | 11.355 | 10.041 | 9.914 | 9.226 | 9.239 | 9.317 | 9.717 |  |
| Ly75 | Lymphocyte antigen 75 | 2.518 | 2.206 | 1.342 | 1.460 | 7.778 | 6.818 | 5.712 | 4.889 | 7.030 | 6.661 | 5.395 | 4.204 |  |
| Ly86 | Lymphocyte antigen 86 | -1.426 | -1.954 | -2.170 | -2.749 | 1.447 | 0.166 | 0.151 | -0.646 | 0.638 | 0.318 | -0.076 | -1.646 |  |
| Ly96 | Lymphocyte antigen 96 | -0.276 | 0.057 | 0.206 | 0.364 | 1.572 | 1.666 | 1.068 | 1.144 | 0.593 | 0.417 | 0.169 | 0.028 |  |
| Mdk | Midkine | -0.695 | -0.398 | 0.707 | 0.986 | 1.103 | 0.868 | 0.855 | 1.140 | 1.749 | 0.184 | 0.484 | -0.026 |  |
| Mefv | Mediterranean fever | 4.547 | 4.237 | 3.261 | 3.755 | 10.142 | 9.296 | 6.287 | 6.754 | 9.226 | 8.833 | 6.213 | 6.515 |  |
| Mgll | Monoglyceride lipase | -2.458 | -1.791 | 0.219 | 1.045 | -1.443 | -1.450 | -1.406 | -1.480 | -1.102 | -2.216 | -2.263 | -2.791 |  |
| Mif | Macrophage migration inhibitory factor | -2.665 | -2.417 | -4.678 | -5.304 | -1.871 | -1.847 | -1.973 | -2.283 | -1.459 | -1.854 | -2.552 | -3.783 |  |
| Mmp25 | Matrix metallopeptidase 25 | 5.627 | 6.041 | 6.091 | 5.647 | 10.198 | 8.985 | 10.185 | 7.909 | 9.201 | 9.239 | 9.317 | 6.972 |  |
| Mpl | Myeloprolif. leukemia virus oncogene | 10.812 | 7.382 | 9.290 | 9.508 | 11.453 | 6.765 | 11.474 | 8.097 | 9.226 | 9.239 | 9.317 | 9.717 |  |
| Mstn | Myostatin | 9.913 | 9.415 | 10.822 | 10.742 | 7.854 | 9.207 | 8.747 | 4.298 | 7.618 | 6.790 | 6.315 | 5.209 |  |
| Muc4 | Mucin 4 | 10.812 | 10.686 | 10.274 | 10.002 | 11.453 | 11.465 | 11.474 | 11.507 | 9.226 | 9.239 | 9.317 | 9.717 |  |
| Myd88 | Myeloid differentiation primary response gene 88 | 2.365 | 2.661 | 2.057 | 2.996 | 6.108 | 5.855 | 5.014 | 5.034 | 8.700 | 8.182 | 7.788 | 5.832 |  |
| Nfam1 | Nfat activating molecule with ITAM motif 1 | 2.668 | 3.028 | 2.756 | 4.549 | 7.668 | 7.584 | 7.552 | 6.898 | 8.312 | 8.228 | 8.505 | 5.665 |  |
| Nfatc3 | Nuclear factor of activated T-cells, cytoplasmic, calcineurin-dependent 3 | -0.835 | -0.774 | -1.363 | -1.410 | 0.959 | 0.982 | 1.092 | 0.926 | 0.781 | -0.01 | -0.329 | -0.715 |  |
| Nfatc4 | Nuclear factor of activated T-cells, calcineurin-dependent 4 | 7.698 | 6.361 | 8.7209 | 8.909 | 11.453 | 9.802 | 11.115 | 10.201 | 9.226 | 9.239 | 9.317 | 7.829 |  |
| Nfe2l1 | Nuclear factor, erythroid derived 2-like 1 | -1.751 | -1.762 | -1.036 | -0.446 | 1.019 | 1.109 | 0.762 | 0.932s | 2.450 | 2.040 | 1.647 | 0.931 |  |
| Nfkb1 | Nuclear factor of kappa light polypeptide gene enhancer in B-cells 1, p105 | -1.370 | -0.985 | -1.722 | -1.345 | 1.622 | 1.446 | 1.361 | 1.323 | 1.134 | 0.675 | -0.044 | -0.604 |  |
| Nfrkb | Nuclear factor related to kappa B binding protein | 1.429 | 1.584 | 1.421 | 1.547 | 4.874 | 4.547 | 4.307 | 4.690 | 4.991 | 5.195 | 4.481 | 3.613 |  |
| Nfx1 | Nuclear transcription factor, X-box binding 1 | 1.478 | 1.437 | 1.466 | 1.203 | 3.663 | 3.065 | 3.202 | 3.759 | 3.467 | 3.656 | 2.811 | 1.872 |  |
| Nlrp12 | NLR family, pyrin domain containing 12 | 8.737 | 10.685 | 6.117 | 6.385 | 9.832 | 11.465 | 10.407 | 9.127 | 9.226 | 9.239 | 9.317 | 9.717 |  |
| Nmi | N-myc (and STAT) interactor | -1.238 | -0.686 | -1.534 | -1.326 | 0.742 | 1.033 | 0.770 | 0.501 | 0.130 | -0.328 | -0.505 | -0.899 |  |
| Nodal | Nodal | 10.812 | 7.593 | 8.214 | 10.742 | 11.453 | 11.465 | 10.028 | 11.507 | 9.226 | 9.239 | 9.317 | 9.717 |  |
| Nos2 | Nitric oxide synthase 2, inducible | 7.803 | 0.400 | 6.821 | 1.575 | 10.717 | 4.073 | 8.770 | 3.855 | 9.197 | 9.239 | 9.317 | 8.897 |  |
| Nr3c1 | Nuclear receptor subfamily 3, group C, member 1 | -1.620 | -1.853 | -1.945 | -2.111 | -0.312 | -0.448 | -0.409 | -0.368 | -0.801 | -0.988 | -1.217 | -1.747 |  |
| Nrg1 | Neuregulin 1 | 7.741 | 7.021 | 3.991 | 3.419 | 8.885 | 9.884 | 7.294 | 6.115 | 9.226 | 9.239 | 5.957 | 5.006 |  |
| Ntf3 | Neurotrophin 3 | 3.270 | 2.789 | 4.731 | 5.228 | 4.028 | 3.918 | 4.260 | 4.086 | 4.176 | 3.318 | 3.543 | 2.999 |  |
| Olr1 | Oxidized low density lipoprotein (lectin-like) receptor 1 | 4.519 | 4.889 | 0.670 | 0.964 | 4.624 | 4.353 | 4.226 | 3.250 | 5.328 | 3.634 | 3.869 | 2.595 |  |
| Osm | Oncostatin M | 3.005 | 3.386 | 2.655 | 2.306 | 6.314 | 7.342 | 6.972 | 5.676 | 8.721 | 7.594 | 6.432 | 4.489 |  |
| Osmr | Oncostatin M receptor | -1.646 | -1.092 | -1.825 | -1.698 | 0.986 | 1.022 | 0.632 | 0.409 | 1.066 | 1.081 | 0.295 | -0.478 |  |
| Parp4 | Poly (ADP-ribose) polymerase family, member 4 | -0.534 | -0.400 | -0.178 | -0.254 | 1.583 | 1.473 | 1.635 | 1.932 | 1.157 | 0.694 | 0.476 | 0.086 |  |
| Nampt | Nicotinamide phosphoribosyltransferase | -2.418 | -1.311 | -1.812 | -2.122 | -1.090 | -0.337 | -1.077 | -0.518 | -1.079 | -0.673 | -2.024 | -1.797 |  |
| Pdgfa | Platelet derived growth factor, alpha | 2.525 | 2.634 | 1.306 | 1.059 | 3.418 | 3.457 | 3.421 | 3.139 | 3.701 | 2.529 | 2.332 | 1.187 |  |
| Pdgfb | Platelet derived growth factor, B polypeptide | 0.747 | 0.297 | 0.633 | 0.275 | 3.633 | 2.831 | 3.337 | 2.691 | 4.887 | 3.782 | 3.597 | 1.903 |  |
| Pdgfc | Platelet-derived growth factor, C polypeptide | 0.108 | 0.668 | 1.171 | 1.405 | 2.028 | 1.916 | 1.529 | 2.139 | 2.451 | 1.746 | 1.572 | 1.260 |  |
| Pf4 | Platelet factor 4 | -2.956 | -2.725 | -1.472 | -1.442 | -1.311 | -1.425 | -1.088 | -1.821 | -0.893 | -1.891 | -1.813 | -2.323 |  |
| Pglyrp1 | Peptidoglycan recognition protein 1 | -0.076 | -0.118 | 0.031 | 0.126 | 2.804 | 2.873 | 2.851 | 2.349 | 3.601 | 3.657 | 2.701 | 1.318 |  |
| Pla2g2d | Phospholipase A2, group IID | -0.287 | -0.648 | 1.120 | 0.672 | 3.006 | 2.927 | 2.111 | 2.886 | 3.487 | 3.160 | 1.726 | 2.018 |  |
| Pla2g7 | Phospholipase A2, group VII (platelet-activating factor acetylhydrolase) | -2.297 | -1.539 | -2.544 | -2.330 | -0.887 | -0.579 | -1.033 | -0.713 | -1.317 | -1.51 | -2.333 | -2.321 |  |
| Ppbp | Pro-platelet basic protein | 5.026 | 5.114 | 4.281 | 4.055 | 5.260 | 5.038 | 5.194 | 3.952 | 4.181 | 3.645 | 3.930 | 3.424 |  |
| Prdx5 | Peroxiredoxin 5 | -3.633 | -2.999 | -3.817 | -3.537 | -3.368 | -3.228 | -3.093 | -2.979 | -3.101 | -3.936 | -4.111 | -4.465 |  |
| Prg2 | Proteoglycan 2 | 0.991 | 1.208 | 1.929 | 2.114 | 5.441 | 5.677 | 5.427 | 6.364 | 8.665 | 8.078 | 6.436 | 5.296 |  |
| Prg3 | Proteoglycan 3 | 5.424 | 4.538 | 4.727 | 5.388 | 11.453 | 8.971 | 11.474 | 9.327 | 9.226 | 9.239 | 9.317 | 8.832 |  |
| Prl | Prolactin | 10.812 | 10.685 | 5.982 | 5.561 | 11.453 | 11.465 | 11.474 | 8.316 | 9.226 | 9.239 | 8.721 | 9.717 |  |
| Prlr | Prolactin receptor | 1.221 | 1.640 | 3.072 | 4.223 | 2.647 | 2.395 | 2.372 | 3.156 | 2.163 | 1.855 | 1.698 | 1.595 |  |
| Procr | Protein C receptor, endothelial | 0.047 | 0.072 | -0.430 | -0.358 | 2.839 | 2.749 | 2.599 | 2.374 | 1.981 | 1.817 | 1.438 | 1.140 |  |
| Prok2 | Prokineticin 2 | 7.177 | 7.056 | 7.229 | 7.546 | 11.453 | 11.210 | 10.413 | 11.507 | 9.226 | 9.239 | 9.317 | 7.556 |  |
| Ptafr | Platelet-activating factor receptor | 0.478 | -0.078 | 1.027 | 1.347 | 2.904 | 1.932 | 2.223 | 1.976 | 3.054 | 1.848 | 2.530 | 1.439 |  |
| Ptgs2 | Prostaglandin-endoperoxide synthase 2 | 4.947 | 5.363 | 3.539 | 2.664 | 5.543 | 6.084 | 5.581 | 5.181 | 8.151 | 8.792 | 6.061 | 4.682 |  |
| Ptn | Pleiotrophin | 5.499 | 6.819 | 7.430 | 9.870 | 3.741 | 6.262 | 3.198 | 5.179 | 8.81 | 5.928 | 8.929 | 7.033 |  |
| Ptpra | Protein tyrosine phosphatase, receptor type, A | -2.618 | -1.976 | -2.324 | -2.367 | -1.062 | -0.948 | -0.913 | -0.868 | -1.494 | -2.092 | -2.038 | -2.446 |  |
| Ptx3 | Pentraxin related gene | 2.296 | 2.125 | 0.478 | -0.416 | 3.029 | 2.865 | 2.728 | 0.672 | -0.380 | 0.735 | 0.419 | 0.804 |  |
| Pxmp2 | Peroxisomal membrane protein 2 | 0.153 | 1.067 | 2.072 | 2.415 | 1.692 | 2.051 | 1.316 | 2.272 | 1.721 | 1.905 | 0.725 | 1.292 |  |
| Reg3a | Regenerating islet-derived 3 alpha | 2.185 | 0.980 | 7.119 | 5.570 | 11.453 | 11.361 | 11.474 | 10.869 | 9.226 | 9.239 | 9.317 | 9.717 |  |
| Reg3g | Regenerating islet-derived 3 gamma | 3.069 | 2.455 | 6.500 | 6.718 | 11.453 | 11.465 | 11.474 | 11.507 | 9.226 | 9.239 | 9.317 | 8.210 |  |
| Ripk2 | Receptor (TNFRSF)-interacting serine-threonine kinase 2 | 0.930 | 0.564 | 0.897 | -0.142 | 2.879 | 2.071 | 2.624016 | 2.194 | 2.580 | 2.121 | 1.432 | 0.541 |  |
| S100a11 | S100 calcium binding protein A11 (calgizzarin) | -5.310 | -5.104 | -5.406 | -5.678 | -4.96 | -5.320 | -4.930 | -5.049 | -5.341 | -5.894 | -6.030 | -6.489 |  |
| S100a8 | S100 calcium binding protein A8 (calgranulin A) | -1.576 | -1.933 | -2.991 | -3.495 | 0.674 | -0.021 | -0.034 | -0.347 | -0.769 | -1.524 | -1.057 | -2.054 |  |
| S100b | S100 protein, beta polypeptide, neural | 2.817 | 2.879 | 4.752 | 4.884 | 3.139 | 3.401 | 3.196 | 3.959 | 2.698 | 2.310 | 1.578 | 2.375 |  |
| Saa4 | Serum amyloid A 4 | 10.812 | 10.685 | 10.822 | 9.346 | 7.742 | 9.468 | 8.995 | 9.280 | 8.032 | 8.223 | 6.006 | 9.717 |  |
| Scg2 | Secretogranin II | 9.942 | 9.488 | 10.822 | 10.742 | 11.453 | 11.465 | 11.474 | 11.507 | 9.226 | 9.239 | 9.317 | 9.717 |  |
| Scube1 | Signal peptide, CUB domain, EGF-like 1 | 8.387 | 8.270 | 8.248 | 8.469 | 10.568 | 11.465 | 11.474 | 11.507 | 7.999 | 9.239 | 9.317 | 9.717 |  |
| Aimp1 | Aminoacyl tRNA synthetase complex-interacting multifunct. protein 1 | -2.494 | -1.994 | -3.365 | -3.618 | -1.131 | -0.949 | -1.370 | -1.318 | -1.228 | -1.778 | -2.237 | -2.473 |  |
| Sdcbp | Syndecan binding protein | -3.857 | -3.732 | -3.905 | -3.904 | -2.302 | -2.632 | -2.560 | -2.639 | -2.486 | -3.318 | -3.335 | -3.767 |  |
| Sectm1b | Secreted and transmembrane 1B | 10.812 | 10.685 | 9.945 | 10.473 | 11.453 | 11.465 | 11.474 | 11.507 | 9.226 | 9.239 | 9.317 | 9.717 |  |
| Sele | Selectin, endothelial cell | 5.028 | 4.911 | 6.140 | 5.557 | 9.220 | 7.006 | 7.810 | 9.931 | 9.129 | 7.456 | 6.490 | 7.029 |  |
| Serpina1a | Serine (or cysteine) peptidase inhibitor, clade A, member 1a | 2.151 | 2.207 | 2.741 | 4.408 | 3.929 | 3.991 | 3.982 | 4.002 | 2.605 | 2.44 | 0.710 | 2.555 |  |
| Serpina3n | Serine (or cysteine) peptidase inhibitor, clade A, member 3N | -2.513 | -1.790 | -1.297 | -0.001 | -3.050 | -3.060 | -2.730 | -2.870 | -2.934 | -3.597 | -3.545 | -3.666 |  |
| Serpinf2 | Serine (or cysteine) peptidase inhibitor, clade F, member 2 | 7.6191 | 7.522 | 5.643 | 6.295 | 11.453 | 9.308 | 11.474 | 8.434 | 3.897 | 4.585 | 5.234 | 3.935 |  |
| Sftpd | Surfactant associated protein D | 10.482 | 8.491 | 10.822 | 10.742 | 11.453 | 11.465 | 11.474 | 11.507 | 9.226 | 9.239 | 9.317 | 9.717 |  |
| Sigirr | Single immunoglobulin and toll-interleukin 1 receptor (TIR) domain | 3.087 | 3.039 | 2.116 | 2.635 | 7.209 | 6.609 | 7.258 | 5.500 | 6.504 | 6.229 | 5.306 | 3.455 |  |
| Siglec1 | Sialic acid binding Ig-like lectin 1, sialoadhesin | -1.429 | -0.066 | -1.711 | -1.735 | 1.797 | 1.433 | 1.421 | 0.831 | 2.476 | 1.895 | 0.862 | -0.426 |  |
| Siva1 | SIVA1, apoptosis-inducing factor | -1.222 | -0.728 | -2.545 | -2.679 | 0.191 | 0.242 | 0.022 | -0.096 | 1.036 | 0.648 | -0.370 | -1.202 |  |
| Slco1a4 | Solute carrier organic anion transporter family, member 1a4 | 5.669 | 7.024 | 8.401 | 9.957 | 9.596 | 11.465 | 10.228 | 11.507 | 9.226 | 9.239 | 9.317 | 9.717 |  |
| Slurp1 | Secreted Ly6/Plaur domain containing 1 | 7.214 | 7.887 | 6.231 | 6.994 | 11.453 | 7.693 | 10.019 | 7.344 | 8.496 | 6.629 | 6.728 | 7.211 |  |
| Socs2 | Suppressor of cytokine signaling 2 | -0.671 | 1.158 | 1.280 | 1.727 | 1.751 | 3.235 | 2.757 | 2.914 | 2.958 | 4.433 | 2.956 | 2.575 |  |
| Spaca3 | Sperm acrosome associated 3 | 10.812 | 10.685 | 10.663 | 10.742 | 11.453 | 11.465 | 11.474 | 11.507 | 9.226 | 9.239 | 9.317 | 9.717 |  |
| Spp1 | Secreted phosphoprotein 1 | 3.178 | 0.892 | -1.565 | -3.826 | 4.146 | 2.142 | 2.571 | -0.097 | 4.324 | 1.749 | 1.308 | -1.973 |  |
| Spred1 | Sprouty protein with EVH-1 domain 1, related sequence | -1.296 | -1.079 | -1.471 | -1.616 | 1.594 | 1.448 | 1.034 | 0.812 | 2.050 | 1.172 | 0.631 | 0.322 |  |
| Srgap1 | SLIT-ROBO Rho GTPase activating protein 1 | 2.424 | 2.623 | 3.737 | 4.922 | 3.733 | 4.420 | 4.309 | 4.626 | 4.739 | 3.699 | 4.034 | 2.970 |  |
| Stab1 | Stabilin 1 | -1.199 | -0.793 | -1.285 | -1.044 | 1.907 | 1.696 | 0.949 | 1.302 | 2.572 | 1.708 | 0.557 | -0.006 |  |
| Stat3 | Signal transducer and activator of transcription 3 | 0.982 | 0.645 | 0.567 | 1.225 | 4.427 | 4.709 | 3.990 | 3.808 | 5.435 | 4.823 | 3.937 | 2.404 |  |
| Sykb | Spleen tyrosine kinase | 0.976 | 1.291 | 1.337 | 2.188 | 5.780 | 5.781 | 5.046 | 5.268 | 5.889 | 6.330 | 5.326 | 4.253 |  |
| Tacr1 | Tachykinin receptor 1 | 3.052 | 4.368 | 4.039 | 5.532 | 11.453 | 9.038 | 9.695 | 9.633 | 9.226 | 9.239 | 9.229 | 8.154 |  |
| Thpo | Thrombopoietin | 7.226 | 7.598 | 9.152 | 8.131 | 11.453 | 10.309 | 10.693 | 8.808 | 9.226 | 9.239 | 9.317 | 9.717 |  |
| Tirap | Toll-interleukin 1 receptor (TIR) domain-containing adaptor protein | 0.761 | 0.646 | 0.418 | 0.151 | 2.851 | 2.621 | 2.364 | 2.455 | 3.527 | 2.749 | 2.022 | 1.477 |  |
| Tlr1 | Toll-like receptor 1 | 1.299 | -0.364 | 1.104 | -0.775 | 5.182 | 2.392 | 2.968 | 2.004 | 5.023 | 2.898 | 3.092 | 1.304 |  |
| Tlr2 | Toll-like receptor 2 | 1.733 | 1.419 | 0.694 | 0.908 | 4.946 | 4.196 | 3.640 | 3.407 | 5.078 | 4.120 | 3.430 | 2.977 |  |
| Tlr3 | Toll-like receptor 3 | 10.812 | 1.140 | 3.133 | 1.985 | 4.959 | 2.779 | 3.641 | 3.174 | 4.777 | 4.502 | 3.773 | 2.336 |  |
| Tlr4 | Toll-like receptor 4 | -1.248 | -0.663 | -0.905 | -0.691 | 0.886 | 1.056 | 0.289 | 0.834 | 5.861 | 1.001 | 0.718 | 0.220 |  |
| Tlr5 | Toll-like receptor 5 | -0.014 | 0.868 | 1.125 | 1.058 | 2.283 | 3.299 | 2.089 | 2.807 | 3.410 | 3.848 | 2.609 | 2.522 |  |
| Tlr6 | Toll-like receptor 6 | 3.484 | 2.027 | 2.762 | 2.246 | 5.724 | 4.886 | 4.807 | 4.180 | 6.328 | 6.669 | 5.718 | 4.360 |  |
| Tlr7 | Toll-like receptor 7 | 1.667 | 0.186 | 1.545 | 0.425 | 4.090 | 2.419 | 2.352 | 2.324 | 5.431 | 6.252 | 4.598 | 3.355 |  |
| Tlr8 | Toll-like receptor 8 | -0.034 | 0.097 | 0.136 | -0.021 | 2.535 | 1.701 | 1.593 | 1.501 | 2.625 | 2.623 | 2.276 | 1.027 |  |
| Tlr9 | Toll-like receptor 9 | 1.519 | 1.071 | 1.433 | 1.682 | 6.339 | 5.492 | 5.767 | 5.798 | 9.226 | 6.091 | 5.778 | 4.856 |  |
| Tnf | Tumor necrosis factor | 4.245 | 2.933 | 3.521 | 3.710 | 8.206 | 7.334 | 6.405 | 6.068 | 9.226 | 7.045 | 5.628 | 4.682 |  |
| Tnfaip6 | Tumor necrosis factor alpha induced protein 6 | 2.919 | 3.367 | 2.592 | 2.175 | 3.442 | 3.018 | 3.653 | 2.233 | 1.064 | 1.534 | 1.267 | 1.428 |  |
| Tnfrsf11b | Tumor necrosis factor receptor superfamily, member 11b (osteoprotegerin) | 3.409 | 0.703 | 3.212 | 2.259 | 2.760 | 1.588 | 2.630 | 2.204 | 4.404 | 3.125 | 3.655 | 2.120 |  |
| Tnfsf10 | Tumor necrosis factor (ligand) superfamily, member 10 | 1.925 | 0.817 | 1.904 | 1.889 | 4.986 | 4.152 | 4.228 | 3.800 | 6.072 | 5.353 | 4.704 | 3.058 |  |
| Tnfsf11 | Tumor necrosis factor (ligand) superfamily, member 11 | 4.389 | 3.032 | 4.470 | 3.166 | 8.993 | 7.814 | 6.771 | 5.624 | 8.662 | 9.096 | 9.317 | 5.630 |  |
| Tnfsf13 | Tumor necrosis factor (ligand) superfamily, member 13 | -0.511 | 0.190 | -0.769 | -0.403 | 3.964 | 3.835 | 2.820 | 3.110 | 4.214 | 3.407 | 1.919 | 2.117 |  |
| Tnfsf13b | Tumor necrosis factor (ligand) superfamily, member 13b | 1.729 | 1.523 | 2.390 | 2.568 | 4.318 | 4.320 | 4.127 | 4.294 | 6.938 | 6.951 | 5.527 | 4.084 |  |
| Tnfsf14 | Tumor necrosis factor (ligand) superfamily, member 14 | 3.177 | 2.402 | 2.294 | 2.051 | 5.338 | 4.919 | 4.491 | 4.731 | 6.349 | 4.962 | 5.004 | 3.394 |  |
| Tnfsf15 | Tumor necrosis factor (ligand) superfamily, member 15 | 7.751 | 5.051 | 5.454 | 3.590 | 11.216 | 9.104 | 8.292 | 7.336 | 9.226 | 9.239 | 9.317 | 6.507 |  |
| Tnfsf18 | Tumor necrosis factor (ligand) superfamily, member 18 | 5.413 | 6.326 | 6.488 | 7.906 | 7.561 | 8.783 | 7.189 | 9.478 | 9.187 | 9.239 | 9.317 | 8.212 |  |
| Tnfsf4 | Tumor necrosis factor (ligand) superfamily, member 4 | 5.542 | 4.886 | 5.468 | 5.040 | 7.836 | 8.162 | 6.851 | 7.366 | 9.226 | 9.239 | 8.330 | 4.332 |  |
| Tnfsf8 | Tumor necrosis factor (ligand) superfamily, member 8 | 4.775 | 3.639 | 4.094 | 3.594 | 11.453 | 9.053 | 8.230 | 7.022 | 9.226 | 9.239 | 9.317 | 7.622 |  |
| Tnfsf9 | Tumor necrosis factor (ligand) superfamily, member 9 | 6.440 | 4.271 | 3.992 | 2.117 | 8.738 | 6.234 | 6.839 | 6.036 | 9.226 | 8.573 | 7.818 | 4.901 |  |
| Tollip | Toll interacting protein | -0.614 | -0.213 | -0.780 | -0.839 | 0.935 | 1.149 | 1.048 | 1.093 | 0.642 | 0.240 | -0.352 | -0.431 |  |
| Tpst1 | Protein-tyrosine sulfotransferase 1 | -1.325 | -1.547 | -1.772 | -1.904 | 0.366 | -0.151 | 0.105 | 0.060 | 1.012 | 0.234 | -0.226 | -1.067 |  |
| Trap1 | TNF receptor-associated protein 1 | -1.650 | -1.205 | -1.558 | -1.604 | -0.950 | -0.735 | -0.756 | -0.743 | -0.452 | -1.585 | -1.887 | -1.742 |  |
| Ttn | Titin | 4.501 | 4.966 | 4.024 | 4.156 | 7.732 | 8.181 | 6.962 | 1.993 | 7.028 | 7.053 | 4.824 | 3.193 |  |
| Tymp | Thymidine phosphorylase | 9.080 | 6.943 | 7.962 | 6.398 | 11.453 | 11.465 | 9.768 | 8.446 | 9.226 | 9.239 | 8.723 | 9.717 |  |
| Vegfa | Vascular endothelial growth factor A | -1.320 | -1.540 | -1.879 | -2.481 | 0.695 | 1.072 | 0.436 | 0.919 | 1.371 | 2.152 | 0.100 | -0.860 |  |
| Vegfb | Vascular endothelial growth factor B | -1.076 | -0.343 | 1.170 | 1.630 | 1.104 | 1.363 | 0.926 | 1.824 | 2.526 | 1.871 | 1.199 | 0.895 |  |
| Vps45 | Vacuolar protein sorting 45 (yeast) | 1.135 | 1.479 | 0.976 | 1.312 | 2.898 | 3.066 | 3.055 | 3.002 | 3.096 | 2.883 | 2.304 | 1.949 |  |
| Xcl1 | Chemokine (C motif) ligand 1 | 3.384 | 2.870 | 1.655 | 1.068 | 5.577 | 5.077 | 4.900 | 2.514 | 5.419 | 3.253 | 2.221 | 1.836 |  |
| Xcr1 | Chemokine (C motif) receptor 1 | 3.311 | 1.856 | 3.319 | 2.658 | 9.272 | 5.999 | 6.236 | 4.662 | 8.207 | 6.427 | 7.096 | 3.930 |  |
| Yars | Tyrosyl-tRNA synthetase | 1.069 | 1.316 | 0.077 | 0.725 | 3.425 | 3.964 | 3.653 | 2.964 | 4.892 | 4.886 | 3.707 | 3.350 |  |
