# Supplementary material for: Obesity modulates the cellular and molecular microenvironment in the peritoneal cavity: implication for ovarian cancer risk
Source: Front Immunol. 2024 Jan 9;14:1323399. doi: 10.3389/fimmu.2023.1323399 (PMC10803595; doi:10.3389/fimmu.2023.1323399)
Supplement: Supplementary file 3 [file Table_2.docx]

**Supplemental Table 2A**

|  | | | Blood | | | | | PSF | | | | OFB | | | | |
| --- | --- | --- | --- | --- | --- | --- | --- | --- | --- | --- | --- | --- | --- | --- | --- | --- |
|  | | Markers | LFD PBS | | HFD PBS | LFD cancer | HFD cancer | LFD PBS | HFD PBS | LFD cancer | HFD cancer | LFD PBS | HFD PBS | LFD cancer | | HFD cancer |
| CD45+ | |  | 98.90  (0.13) | | 98.84 (0.26) | 99.08 (0.29) | 98.10  (0.19) | 99.76 (0.32) | 90.84 (8.16) | 99.38 (0.15) | 99.60 (0.17) | 86.58 (2.31) | 80.66  (2.67) | 94.43 (0.66) | 93.44^e^  (1.78) | |
| Percent of CD45^+^ (SEM) | | | | | | | | | | | | | | | | |
| B cells | | CD19+ | 54.50 (3.43) | 17.88^c^ (1.44) | | 43.18^a^ (1.85) | 20.21^g^ (3.05) | 56.94 (6.35) | 32.24^a^ (4.46) | 43.40 (3.06) | 26.65 (6.67) | 51.4 (3.11) | 44.26 (3.10) | 35.02^a^ (5.01) | 33.12 (4.52) | |
| Percentage of Parent | B1 | CD19+CD11b+ | 3.46 (0.92) | 1.97 (0.34) | | 0.89 (0.29) | 4.28 (1.22) | 39.30 (2.27) | 46.00 (5.18) | 40.53 (6.03) | 26.02^d^ (4.30) | 14.18 (0.53) | 18.32 (12.39) | 20.45 (3.28) | 16.53 (3.28) | |
|  | B2 | CD19+CD11b- | 95.60 (0.99) | 97.76 (0.40) | | 98.33 (0.32) | 95.26 (1.39) | 58.46 (2.32) | 51.90  (5.30) | 57.70 (6.16) | 72.68^d^ (4.34) | 84.00 (0.57) | 80.52 (12.50) | 75.93 (3.19) | 85.58 (3.46) | |
| T Cells | | CD3+ | 27.10 (3.04) | 47.28^a^ (1.73) | | 20.00 (1.37) | 30.12^d^ (6.66) | 6.98 (0.92) | 9.56 (1.04) | 4.52 (1.43) | 20.42^d,h^  (4.73) | 30.52 (2.79) | 28.76 (1.92) | 19.40 (2.95) | 17.97^e^ (3.07) | |
|  | Th | CD3+CD4+ | 40.62 (3.49) | 48.86 (2.28) | | 41.48 (2.05) | 38.80 (2.71) | 49.40 (3.79) | 53.50 (1.94) | 48.83 (1.64) | 52.26 (3.45) | 41.70 (2.08) | 39.20 (4.47) | 37.08 (2.38) | 38.22 (4.33) | |
| Percentage of parent | Tc | CD3+CD8+ | 49.64 (2.94) | 43.22 (2.19) | | 47.15 (1.56) | 50.24 (1.81) | 26.14 (3.44) | 29.76 (2.57) | 28.50 (0.83) | 35.28 (3.02) | 21.28 (2.05) | 28.02 (1.91) | 25.75 (1.09) | 24.94 (3.16) | |
|  | DN | CD4-CD8- | 9.25 (0.92) | 18.70 (1.88) | | 11.17 (1.20) | 27.54^h^ (4.10) | 23.36 (4.66) | 3.92^c^ (0.51) | 22.25 (2.51) | 1.19^i^ (0.30) | 36.22 (3.51) | 10.84^c^ (1.55) | 36.30 (1.47) | 16.70^h^ (3.10) | |
| Mono-granulocytes | | CD11b+, SSCv | 5.56 (0.87) | 5.16 (0.42) | | 21.63^a^ (2.36) | 19.08d (5.23) | 15.22 (1.63) | 32.48^a^ (4.74) | 42.10^a^ (1.46) | 37.76 (9.18) | 11.75 (2.32) | 15.40 (2.79) | 36.20 (8.25) | 35.42 (10.38) | |
| Percentage of parent | MDSC-Gran (SP1) | CD11bhi,Ly6Ghi,  Ly6C+ | 42.36  (5.21) | 54.70  (3.12) | | 72.42^b^  (4.96) | 70.68  (5.52) | 0.25  (0.13) | 0.27  (0.15) | 25.28^b^  (5.60) | 48.72^e^  (13.72) | 1.49  (0.36) | 6.53  (0.92) | 41.88^b^  (7.30) | 51.20^c^  (10.47) | |
|  | DCs (SP2) | CD11chi | 20.80  (4.49) | 12.18  (1.02) | | 6.12  (0.84) | 4.67  (0.90) | 3.58  (0.25) | 5.08  (0.85) | 4.89  (1.45) | 4.17  (0.78) | 15.60  (0.66) | 14.75  (2.67) | 12.18  (1.02) | 11.60  (3.75) | |
|  | Resident PM (SP-3) | F480hi, Ly6Clo, CD93+ | 6.29  (2.21) | 1.26  (0.59) | | 0.75^a^  (0.54) | 1.52  (0.86) | 43.26  (3.69) | 73.46^b^  (5.56) | 26.80  (2.62) | 22.66f  (7.40) | 5.75  (1.71) | 0.84^b^  (0.11) | 5.12  (0.63) | 1.41  (0.32) | |
|  | Monocytes (SP-6) | F4/80lo, Ly6Chi | 3.39  (0.39) | 3.49  (0.52) | | 1.22^b^  (0.25) | 2.01  (0.40) | 5.86  (1.35) | 6.86  (1.42) | 12.87^a^  (1.31) | 8.65  (2.39) | 24.88  (2.03) | 31.34  (1.96) | 15.78^a^  (2.01) | 13.65^c^  (2.65) | |
| NK | | NK1.1+, CD3- | 7.82 (1.41) | 8.85 (0.96) | | 6.45 (1.07) | 9.02 (0.82) | 0.72 (0.06) | 2.35^b^ (0.18) | 0.95 (0.17) | 1.41 (0.18) | 2.66 (0.30) | 2.35 (0.34) | 3.77 (0.42) | 1.85^g^ (0.39) | |
| NKT | | NK1.1+,CD3+ | 0.92 (0.03) | 1.06 (0.12) | | 0.71 (0.03) | 0.77 (0.09) | 0.55 (0.09) | 0.49 (0.06) | 0.46 (0.06 | 0.38 (0.10) | 2.59 (0.14) | 1.19^c^ (0.12) | 1.76^a^ (0.18) | 1.27 (0.22) | |

Table 2: Changes in immune cell subtypes induced by a high-fat diet (HFD) or MOSE-L_TIC_*_v_* (cancer) injection in the peritoneal serous fluid (PSGF), blood, and omental fat band (OFB).

^a^ p<0.05 vs LFD; ^b^ p<0.01 vs LFD; ^c^ p<0.001 vs LFD; ^d^ p<0.05 vs HFD; ^e^ p<0.01 vs HFD; ^f^ p<0.001 vs HFD; ^g^ p<0.05 vs LFD cancer; ^h^ p<0.01 vs LFD cancer*;* ^i^ p<0.001 vs LFD cancer

**SUPPLEMENTAL TABLE 2B**

|  | | | pmWAT | | | | rpWAT | | | |
| --- | --- | --- | --- | --- | --- | --- | --- | --- | --- | --- |
|  | | Markers | LFD PBS | HFD PBS | LFD MOSE-L_TIC_*_v_* | HFD MOSE-L_TIC_*_v_* | LFD PBS | HFD PBS | LFD  MOSE-L_TIC_*_v_* | HFD MOSE-L_TIC_*_v_* |
| CD45+ | |  | 59.26  (6.04) | 59.96  (3.68) | 72.00  (3.95) | 68.78  (3.23) | 50.46  (2.64) | 55.32  (3.89) | 52.43  (11.33) | 51.94  (5.32) |
| Percent of CD45^+^ (SEM) | | |  |  |  |  |  |  |  |  |
| B cells | | CD19+ | 5.06 (0.41) | 4.07 (0.57) | 3.06 (0.13) | 4.39 (0.81) | 10.71 (1.49) | 17.86 (4.58) | 12.24 (1.54) | 14.27 (2.27) |
| Percent of Parent | B1 | CD19+CD11b+ | 10.88 (0.35) | 18.36^b^ (1.85) | 10.86 (1.40) | 8.55^f^ (1.61) | 3.46 (0.92) | 1.97 (0.34) | 0.89 (0.29) | 4.28 (1.22) |
|  | B2 | CD19+CD11b- | 88.08  (0.60) | 80.50^a^  (2.13) | 88.42 (1.74) | 90.96^e^ (1.68) | 88.68 (1.44) | 81.10 (4.87) | 86.70 (1.88) | 85.02 (2.10) |
| T Cells | | CD3+ | 13.27  (1.03) | 10.36 (0.84) | 14.27  (1.50) | 10.84 (0.82) | 16.55 (2.59) | 17.86 (4.14) | 16.86 (1.57) | 10.29 (1.46) |
| Percent of Parent | Th | CD3+CD4+ | 28.56  (2.42) | 30.98  (4.24) | 32.18  (2.93) | 29.20  (2.96) | 31.04 (2.98) | 35.80 (3.29) | 27.56 (2.90) | 25.38 (4.73) |
|  | Tc | CD3+CD8+ | 17.04  (1.91) | 15.02  (1.67) | 21.12 (1.58) | 21.84 (2.42) | 22.78 (3.41) | 26.46 (4.29) | 26.72 (3.57) | 27.00 (3.04) |
|  | DN | CD4-CD8- | 52.50  (3.44) | 53.44  (3.69) | 45.98 (2.66) | 48.46 (3.56) | 43.70 (3.41) | 36.72 (5.66) | 44.52 (5.41) | 46.20 (4.70) |
| Mono-granulocytes | | CD11b+, SSCv | 41.94  (2.18) | 50.28^a^  (2.52) | 46.82 (2.93) | 54.52 (3.81) | 48.92 (8.10) | 44.86 (5.22) | 53.84 (6.52) | 53.84 (6.52) |
| Percent of Parent | MDSC-Gran (SP1) | CD11bhi,Ly6Ghi,  Ly6C+ | 0.59  (0.13) | 1.80  (0.27) | 1.40  (0.29) | 8.74  (4.25) | 2.23  (1.31) | 2.27  (0.35) | 2.31  (1.22) | 3.33  (0.37) |
|  | DCs (SP2) | CD11chi | 8.79  (1.74) | 12.01  (3.15) | 6.86  (0.58) | 9.00  (0.56) | 10.06  (1.18) | 7.12  (0.76) | 13.89  (2.64) | 7.55  (1.71) |
|  | Resident PM (SP-3) | F480hi,Ly6Clo, CD93+ | 1.23  (0.16) | 2.20  (0.91) | 1.13  (0.18) | 3.34  (0.75) | 1.47  (0.37) | 3.99^a^  (0.77) | 1.63  (0.68) | 4.32^g^  (0.55) |
|  | Monocytes (SP-6) | F480lo, Ly6Chi | 53.68  (1.29) | 62.56  (4.79) | 55.48  (0.54) | 59.90  (4.40) | 50.00  (1.97) | 59.86  (2.70) | 48.62  (4.35) | 63.60  (2.81) |
| NK | | NK1.1+, CD3- | 9.82  (0.83) | 10.01  (1.42) | 10.11  (0.80) | 9.53  (0.69) | 9.47  (1.51) | 9.74  (2.40) | 12.97  (2.08) | 9.69  (0.54) |
| NKT | | NK1.1+,CD3+ | 1.89  (0.23) | 1.76  (0.22) | 2.32  (0.46) | 2.08  (0.10) | 2.71  (0.52) | 2.42  (0.82) | 2.83  (0.49) | 2.04  (0.37) |

Table S2B: Changes in immune cell subtypes induced by a high-fat diet (HFD) or MOSE-L_TIC_*_v_* (TIC*v*) injection in the parametrial white adipose tissue (pmWAT) and retroperitoneal white adipose tissue (rpWAT) (OFB).

^a^ p<0.05 vs LFD; ^b^ p<0.01 vs LFD; ^c^ p<0.001 vs LFD; ^d^ p<0.05 vs HFD; ^e^ p<0.01 vs HFD; ^f^ p<0.001 vs HFD; ^g^ p<0.05 vs LFD TIC*v*; ^h^ p<0.01 vs LFD TIC*v;* ^i^ p<0.001 vs LFD TIC*v*
