# Supplementary material for: Obesity modulates the cellular and molecular microenvironment in the peritoneal cavity: implication for ovarian cancer risk
Source: Front Immunol. 2024 Jan 9;14:1323399. doi: 10.3389/fimmu.2023.1323399 (PMC10803595; doi:10.3389/fimmu.2023.1323399)
Supplement: Supplementary file 4 [file Table_3.docx]

**SUPPLEMENTAL TABLE 3**

| Table S3: Gene Changes induced by the HFD and MOSE-L_TIC_*_v_* injections | |
| --- | --- |
| **OFB** | |
| Genes increased with a HFD | Apcs, Bmp7, Ccl11, Cd180, Csf2, Cyp26b1, Fgf5, Fgf7, Fgf12, Hrh1, Il3, Il4, Il17f, Itih4, Nfatc4, Reg3a, Sftpd, Tlr1, Tlr6, Tlr7, Tnf, Tnfsf8, Tnfsf10, Tnfsf11, Xcr1 |
| Genes increased with MOSE-L_TIC_*_v_* | Ahsg, Aif1, Aimp1, Apoa2, Ccl1, Ccl3, Ccl4, Ccl17, Ccl20, Ccl25, Cd70, Csf1, Csf3,Csf3r, Ctf1, Ctf2, Cxcl9, Cxcl10, Cxcl14, Cxcl16, Cxcr1, Cxcr2, F11r, F2 Fn1, Gdf3, Gdf6, Gpi1, Hdac7, Ifne, Il1f9, Il1r1, Il1r2, Il1rap, Il1rl2, Il1rn, Il6, Il10, Il12a, Il13ra1, Il18, Il18r1, Il28ra, Inhba, Irf7, Lefty1, Lif, Ly75, Mefv, Mif, Nlrp12 Nrg1, Olr1, Pdgfa, Prl, Ptgs2, Ptx3, S100a8, Serpinf2, Siva1, Slurp1, Tlr2, Xcl1 |
| Genes increased with both HFD and MOSE-L_TIC_*_v_* | Apol7a, Areg, Blnk, Ccl2, Ccl7, Ccl12, Ccr2, Ccr3, Ccr5, Ccr6, Cx3cr1, Cxcl1, Cxcl2, Cxcl5, Gdf5, Ifna2, Ifna4, Ifng, Ifngr1, Il1b, Il21, Il23a, Kng1, Mpl, Nodal, Nos2, Spp1, Tlr3, Tnfsf9, Tnfsf15, Tymp |
| Genes decreased with a HFD | Ccl27a, Ccr7, Ccr8, Cd70, Cebpb, Csf3 Ctf2, Cxcr4, Gpr68, Hdac4, Ik, Il7r, Il11, Il12b, Il12rb2, Il22, Nlrp12, Nampt, Siglec1 |
| Genes decreased with MOSE-L_TIC_*_v_* | Adora1, Bmp2, Bmp3, C3, Ccl11, Ccl24, Ccl28, Ccrl1, Cxcl11, Cxcl12, Eda, Ephx2, F3, Fgf1, Figf, Flt3l, Gdf9, Gfra2, Ghr, Grem2, Ifngr1, Il13, Il17c, Il7, Lbp, Lepr, Ltb4r1, Mdk, Nfatc4, Ntf3, Pdgfc, Pf4, Pla2g2d, Prg2, Prlr, Reg3a, Reg3g, S100b, Sele, Srgap1, Thpo, Tlr5, Vegfb |
| Genes decreased with both HFD + MOSE-L_TIC_*_v_* | Ccl6, Cntfr, Fgf2, Fgf6, Fgf9, Gfra1, Il2, Il6ra, Il13ra2, Il15ra, Il17b, Il17d, Il19, Il20ra, Il22ra2, Il31ra, Ins2, Mgll, Lefty2, Ptn, Pxmp2, Serpina3n, Slco1a4, Socs2, Tacr1 Tnfsf18, |
| Increased by HDF over LFD + MOSE-L_TIC_*_v_* | Ccl12, Ccl17, Ccr2, Ccr3, Cer1, Cxcl1, Cxcl2, Cxcl5, Cxcl15, Fgf12, Fos, Gdf2, Ifna2, Ifna14, Ifna9, Ifnab, Ifnb1, Ifne, Il1b, Il13, Il17b, Il17f, Il19, Il31, Il4, Ins1, Lefty2, Nos2, Reg3a, Saa4, Spp1, Thpo, Tlr1, Tlr3, Tlr7, Tnfsf9, Tnfsf11, Tnfsf15, Tymp |
| Decreased by HDF over LFD + MOSE-L_TIC_*_v_* | Adora1, Ahsg, Apoa2, Ccl6, Ccl24, Cd70, Cntfr, Crp, Csf2ra, Ctf1, Cxcl13, Eda, F2, Fpr1, Gdf1, Gdf5, Gdf7, Gfra1, Ghr, Grem2, Ik, Il3ra, Il12rb2, Il17rb, Il20ra, Il22ra2, Il23a, Il15ra, Il7, Inhbb, Ins2, Kng1, Ltb4r1, Myd88, Nfam, Nodal, Prlr, Ptn, Serpina1a, Serpina3n, Slco1a4, Srgap1, Tacr1, Tnfsf18 |
| **pmWAT** | |
| Genes increased with a HFD | Ccl22, Ccl7, Ccr2, Ccr3, Ccr4, Ccrl2, Cd28, Fgf3, Gdf2, Hdac9, Ifna2, Il7r, Il10, Il17c, f, Intb2, Lefty2, Mmp25, Mpl, Nfat4c, Prg3, Serpinf2, Thpo, Tnfsf11b |
| Genes increased with MOSE-L_TIC_*_v_* | Apol8, Ccr10, Ccr7, Ccr9, Cd40lg, Cmtm2a, Cxcl1, C3cr1, Cxcl11, Dock2, Fn1, Fpr1, Gdf1, Il13ra1, Il16, Il18r1, Il19, Il1b, Il1f9, Il1rap, Il20ra, Il21r, Il23a, Il24, Il27, Il28ra, Il3ra, Cxcr1, 2, Il9r, Inhbb, Irf7, Lif, Ltb, Ly75, Mefv, Myd88, Nodal, Nrg1, Prok2, Tlr2, Tnf, Tnfsf4, Tnfsf13, Tymp |
| Genes increased with both HFD and MOSE-L_TIC_*_v_* | Aif1, Areg, Blnk, C3ar1, Ccl1, Ccl17, Ccl2, Ccl4, Ccr5, Ccr6, Cd180, Cd4, Clcf1, Csf2, Csf3, Csf3r, Cxcl10, Cxcl5, Cxcr5, Cybb, Fasl, Gdf3, Grem1, Ifng, Il12a, Il13, Il18r1, Il18r2, Il18rap, Il1a, Il1rn, Il21, Il22ra1, Il23r, Kng1, Lta, Ly86, Nos2, Sele, Slurp1, Spp1, Tacr1, Tlr1, Tlr3, Tlr7, Tlr8, Tnf, Tnfsf11, Tnfsf15, Tnfsf8, Tnfsf9, Xcr1 |
| Genes decreased with a HFD | Ccr10, Cd70, Cebpb, Cntfr, Ctfr, Ctf1, Ctf2, Cxcl13, Cxcr4, Erbb2, Fgf5, Fos, Hdac4, Ifne, Il12b, Il5, Ins2, Irf4, Lepr, Ltb4r1, Mstn, Nlpr12, Osm, Ptn, Slco1a4, Tlr5, Tnfsf18, |
| Genes decreased with MOSE-L_TIC_*_v_* | Bmp7, Ccrl1, Fgf12, Il13ra2, Il4, Ithih4 |
| Genes decreased with both HFD + MOSE-L_TIC_*_v_* | Ik, Il5, Il17b, Il1f6, Il1f8, Saa4, Socs2 |
| Increased by HDF over LFD + MOSE-L_TIC_*_v_* | Apol7a, Areg, Bmp7, Ccl1, 12, 17, 2,20,22,4,5,7,8, Ccr1,2,3,4,5,8, Csf2, Cxcl10,15,9, Cyp26b1, F2, Fasl, Fgf12, 3, Fos, Gdf2,6, Hrh1, Ifna2, Il12b, Il13ra2, Il17c,f, Il18rap, Il1a, Il1rn, Il4, Il6, Intb2, Lefty2, Lta, Mmp25, Mpl, Mstn, Nlrp12, Nos2, Nrg1, Olr1, Osm, Ppbp, Prg3, Prl, Ptx3, SerpinF2, Sigirr, Slurp1, Spp1, Thpo, Tnfaip6, Tnfsf11, Tnfsf8, Ttn, Tymp, Xcr1 |
| Decreased by HDF over LFD + MOSE-L_TIC_*_v_* | Apol8, Ccl24, Ccrl1, Cfs3, Cxcl13, Cxcr1, Cxcr5, Fgf5, Fpr1, Gdf5, Grem2, Ifne, Il19, Il1f9, Il20ra, Il22ra2, Il31ra, Inha, Irf4, Kng1, Lepr, Nodal, Prok3, Ptn, Sele, Slco1a4, Tnfsf18 |
| **rpWAT** | |
| Genes increased with HFD | Bmp7, Ccl24, Ccl3, Ccr2, Ccrl1, Ccrl2, Cd180, Cd27, Cxcl14, Fgf7, Fgf10, Fgf12, Gdf3, Il17b, Il1rl2, Il1rn, Il17r, Ins2, Lbp, Nampt, Ptafr, Ptn, Srgap1, Tnfrsf11b |
| Genes increased with MOSE-L_TIC_*_v_* | Apoa2, Apol7a, Bmp3, Ccl1, Ccl12, Ccl8, Ccr1, Ccr9, Cd27, Cd28, Cd4, Crp, Csf1, Ctf1, Cx3cr1, Cxcl11, Cxcl5, Cxcr5, F11r, F2, Fgf2, Flt3l, Fn1, Fpr1, Gdf6, Gdf7, Gpi1, Hdac7, Hrh1, Ifnar1, Ifng, Ifngr1, Il10ra, Il11ra, Il12a, Il12b, Il13ra1, Il18, Il18r1, Il1f9, Il1r1, Il21r, Il22ra1, Il2rb, Il2rg, Il6st, Cxcr2, Inha, Irf4, Irf7, Kng1, Lefty1, Lepr, Lif, Lifr, Ltb, Ly75, Mefv, Mif, Mstn, Nfatc3, Nfkb1, Nrg1, Pla2g2d, Pla2g7, Prdx5, Prg2, Ptgs2, Pxmp2, Ripk2, S100b, Saa4, Aimp1, Serpina1a, Sigirr, Siglec1, Siva1, Spred1, Stab1, Stat3, Tirap, Tlr2, Tlr3, Tnfsf10, 13, 13b, 9, Tpst1, Ttn, Vegfa,b, Yars |
| Genes increased with both HFD and MOSE-L_TIC_*_v_* | Adora1, Ahsg, Aif1, Areg, C3ar1, Ccl2, Ccl22, Ccl4, Ccl5, Ccl7, Ccr4, Ccr5, Ccr7, Clcf1, Csf2, Csf2ra, Csf3r, Cx3cl1, Cxcl1, Cxcl10, Cxcl12, Cxcl16, Cxcl9, Cxcr3, Cybb, Ebi3, F3, Gdf5, Ghr, Gpr68, Hdac9, Ifngr2, Il2ra, Il10, Il15, Il16, Il17rb, Il18rap, Il1rap, Il2, Il6, Inhba, Inhbb, Itih4, Mdk, MgII, Olr1, Osm, Pdgfa, Pdgfb, Pf4, Sele, Slurp1, Spp1, Tlr1, Tlr4, Tlr9, Tnfrsf14, Trap1, Xcl1, Xcr1 |
| Genes decreased with HFD | Ccl25, Ccr8, Crp, Cxcl13, Fos, Gfra1, Grem2, Il12b,  Il1b, Il27, Il31ra, Irf4, Ptx3, Soc2, |
| Genes decreased with MOSE-L_TIC_*_v_* | Il13ra2, Il1a, Serpinf2 |
| Genes decreased with both HFD and MOSE-L_TIC_*_v_* | Apcs, Ccl11, Cmt2a, Il11, Il17d, Il1a, Il5ra, Scube |
| Increased by HDF over LFD + MOSE-L_TIC_*_v_* | Apol7a, Areg, Blnk, Bmp1, Bmp2, C3ar1, Ccl11,12,2,24,25, 28, 3, 4, 5,7, Ccr1, 10, 2, 3, 5, 6, 8, 9, Ccrl1, Ccrl2, Cd180, Cd27, Cd28, Cd40, Cd40lg, Cd70, Cd74, Cd97, Clcf1, Cmtm2a, Csf1, Csf2, Cxcl1, 10, 11, 16, 2, 5, 9, Cxcr3, Cxcr6, Ephx2, Epor, Erbb2, Fasl, Fgf10, Fgf12, Fgf7, Fgf9, Fos, Gdf3, gdf5, Hdac9, Ifnar1,Ifng, Ifngr2, Ifnk, Il10, Il10ra, Il12b, Il13ra2, Il16, 17b, 17d, 17f, Il18r1, Il18rap, Il1a, 1b, 1rn, 21, Il21r, Il22ra2, Il2rb, Il3ra, Il14ra, Il5, Il5ra, Il6, Il7r, Inhba, Inhbb, Lbp, Lif, Lta, Ltb, Ly75, Ly86, Mif, Mmp25, Mstn, Myd88, Nfam1, Nfatc4, Nrg1, Olr1, Osm, Pdgfa,b, Pglyrp1, Prg2, Prok2, Ptafr, Ptgs2, Ptn, Reg3, S100a8, Serpinf2, Sigirr, Siglec1, Spp1, Srgap1, Stat3, Sykb, Tacr1, Tlr1,3,6,8, Tnf, Tnfsf11b, 10, 11, 13b, 14,15, 18,4,8,9, Tnt, Vegfa, Xcr1 |
| Decreased by HDF over LFD + MOSE-L_TIC_*_v_* | Ahsg, Apcs, Apoa2, Crp, Cxcl13, F2, Fpr1, Il13, Inha, Irf4, Kng1, Itih4, Saa4, Serpina1a |
