# Supplementary material for: Obesity modulates the cellular and molecular microenvironment in the peritoneal cavity: implication for ovarian cancer risk
Source: Front Immunol. 2024 Jan 9;14:1323399. doi: 10.3389/fimmu.2023.1323399 (PMC10803595; doi:10.3389/fimmu.2023.1323399)
Supplement: Supplementary file 5 [file Table_4.docx]

**SUPPLEMENTAL TABLE 4**

Table S4: qPCR analyses of the pmWAT and rpWAT (ΔCT±SEM)

| 1. **pmWAT** | | | | | |
| --- | --- | --- | --- | --- | --- |
|  | Gene | LFD | HFD | LFD MOSE-L_TIC_*_v_* | HFD MOSE-L_TIC_*_v_* |
| Adipokines | Adiponectin | -5.05±0.28 | -5.33±0.20 | -5.04±0.47 | -4.97±0.47 |
|  | Adipsin | 16.18±1.03 | 15.16±1.00 | 15.26±1.01 | 14.89±1.13 |
|  | Leptin | 1.33±1.08 | -0.43±0.34 | 0.66±0.76 | -0.45±0.74 |
|  | Resistin | -4.19±0.28 | -3.83±0.22 | -3.86±0.46 | -3.34±0.40 |
|  | Visfatin | 1.93±0.31 | 1.92±0.31 | 1.50±0.22 | 1.99±0.25 |
| Cyto- and chemokines | Ccl1 | 16.47±0.74 | 15.55±0.34 | 15.93±0.91 | 13.68±1.08 |
|  | Ccl2 | 4.93±0.38 | 2.80±0.29^a^ | 4.22±0.59 | 1.94±0.36^d^ |
|  | Ccl4 | 9.34±0.39 | 7.54±0.39 | 9.14±0.61 | 6.88±0.45^d^ |
|  | Ccl5 | 3.67±0.29 | 2.75±0.38 | 3.32±0.45 | 2.19±0.29 |
|  | Ccr5 | 6.80±0.44 | 4.81±0.49 | 5.88±0.58 | 4.56±0.60 |
|  | Cx3cl1 | 8.69±0.48 | 8.19±0.59 | 7.71±0.81 | 7.09±0.35 |
|  | Cxcl13 | 7.23±0.97 | 7.54±0.87 | 7.80±0.98 | 8.32±0.41 |
|  | Cxcr2 | 14.72±1.08 | 13.48±1.02 | 11.46±1.19 | 10.19±1.02 |
|  | IFNγ | 12.74±0.97 | 10.54±0.19 | 11.18±0.66 | 10.01±0.41 |
|  | IL-1b | 13.65±0.56 | 12.25±0.22 | 12.06±0.99 | 10.42±0.72 |
|  | IL-6 | 15.91±0.34 | 14.11±0.43 | 15.51±0.61 | 12.71±0.98^d^ |
|  | IL-10 | 15.27±0.52 | 14.34±0.54 | 16.32±0.59 | 14.75±0.75 |
|  | eNOS | 10.54±0.44 | 10.67±0.19 | 10.29±0.57 | 10.90±0.49 |
|  | TNFα | 10.54±0.75 | 7.94±0.52 | 8.70±0.73 | 7.60±0.69 |
| Adhesion, growth, and angiogenesis factors | Agt | 5.59±0.37 | 5.87±0.34 | 5.57±0.37 | 6.24±0.31 |
|  | CD31 | 5.24±0.51 | 5.22±0.28 | 5.29±0.59 | 5.36±0.33 |
|  | CD34 | 5.34±0.72 | 5.05±0.34 | 5.04±0.73 | 5.17±0.27 |
|  | Col1 | 12.49±1.30 | 11.92±0.54 | 10.90±0.96 | 10.92±0.61 |
|  | FN1 | 10.61±0.91 | 10.33±0.13 | 8.23±0.86 | 8.08±0.93 |
|  | HIF1α | 5.85±0.48 | 5.64±0.22 | 5.59±0.34 | 4.98±0.63 |
|  | IDO | 14.43±0.79 | 14.70±0.29 | 14.26±0.26 | 15.23±0.31 |
|  | IGF1 | 3.63±0.23 | 3.52±0.20 | 4.06±0.31 | 3.43±0.15 |
|  | MMP11 | 8.96±0.66 | 7.70±0.61 | 7.79±0.78 | 8.24±0.36 |
|  | Pai-1 | 4.51±0.83 | 4.99±0.55 | 4.74±0.50 | 4.26±0.54 |
|  | aSMA | 1.41±0.78 | 1.34±0.58 | 1.32±0.82 | 1.04±0.27 |
|  | TGFβ | 7.66±0.51 | 6.63±0.33 | 7.07±0.49 | 6.49±0.56 |
|  | TnC | 14.76±0.59 | 14.78±0.30 | 13.15±1.27 | 14.00±0.64 |
|  | UCP1 | 10.58±0.76 | 10.88±1.27 | 7.60±1.93 | 8.92±0.37 |
|  | VCAM | 5.95±0.42 | 6.49±0.13 | 5.73±0.36 | 5.90±0.44 |
|  | VE-cadh | 5.78±0.47 | 5.33±0.20 | 5.69±0.48 | 5.24±0.57 |
|  | VEGFa | 6.02±0.28 | 6.42±0.26 | 5.75±0.41 | 5.98±0.44 |
|  | VEGFR2 | 7.77±0.45 | 7.17±0.32 | 7.48±0.46 | 6.92±0.38 |
|  | VLDLR | 3.80±0.30 | 3.36±0.13 | 3.93±0.45 | 4.02±0.53 |

| 1. **rpWAT** | | | | | |
| --- | --- | --- | --- | --- | --- |
|  | Gene | LFD | HFD | LFD MOSE-L_TIC_*_v_* | HFD MOSE-L_TIC_*_v_* |
| Adipokines | Adipsin | 8.59±0.42 | 6.89±0.37^a^ | 6.91±0.22^a^ | 5.71±0.34 |
|  | Adiponectin | -4.80±0.42 | -5.19±0.23 | -5.53±0.53 | -5.35±0.81 |
|  | Leptin | 2.70±1.11 | 1.00±0.40 | 1.81±0.88 | 0.61±0.76 |
|  | Resistin | -3.67±0.29 | -3.54±0.32 | -4.12±0.55 | -3.59±0.82 |
|  | Visfatin | 2.16±0.19 | 2.79±0.16 | 1.35±0.21^a^ | 1.63±0.09^g^ |
| Cyto- and chemokines | Ccl1 | 17.18±0.51 | 15.37±0.36 | 15.57±1.06 | 14.58±0.66 |
|  | Ccl2 | 4.29±0.29 | 2.02±0.43^b^ | 3.22±0.48 | 1.76±0.36 |
|  | Ccl4 | 10.27±0.29 | 7.96±0.48 | 8.74±0.66 | 6.78±0.75 |
|  | Ccl5 | 3.89±0.36 | 2.87±0.46 | 2.80±0.44 | 1.66±0.34 |
|  | Ccr5 | 7.88±0.35 | 5.74±0.25^b^ | 6.59±0.52 | 5.20±0.44 |
|  | Cx3cl1 | 9.91±0.16 | 8.96±0.39 | 8.69±0.61 | 8.22±0.60 |
|  | Cxcl13 | 7.16±0.85 | 7.45±0.64 | 7.68±1.08 | 7.83±0.53 |
|  | Cxcr2 | 14.01±0.69 | 13.73±0.67 | 11.50±1.52 | 9.93±1.04 |
|  | IFNγ | 12.82±0.59 | 11.55±0.65 | 11.29±0.54 | 10.51±0.70 |
|  | IL-1β | 13.95±0.62 | 12.89±0.45 | 12.56±0.68 | 11.27±0.84 |
|  | IL-6 | 15.45±0.32 | 14.69±0.72 | 15.36±0.22 | 13.74±0.58^d^ |
|  | IL-10 | 15.75±0.30 | 16.34±0.33 | 16.17±0.39 | 15.66±0.67 |
|  | eNOS | 11.03±0.68 | 11.71±0.38 | 11.13±0.42 | 11.13±0.74 |
|  | TNFα | 9.86±0.46 | 7.81±0.34^a^ | 9.17±0.49 | 8.06±0.44 |
| Adhesion, growth, and angiogenesis factors | Agt | 5.42±0.63 | 7.18±0.43 | 6.47±0.26 | 7.38±0.69 |
|  | CD31 | 6.83±0.27 | 6.64±0.29 | 6.36±0.29 | 5.63±0.56 |
|  | CD34 | 6.95±0.54 | 2.51±0.68 | 2.55±0.57 | 1.54±0.58 |
|  | Col1 | 14.48±0.67 | 13.70±1.49 | 13.52±0.67 | 12.24±0.59 |
|  | FN1 | 11.08±0.40 | 11.14±0.40 | 9.23±0.95 | 9.33±1.08 |
|  | HIF1α | 6.44±0.75 | 6.73±0.33 | 6.68±0.19 | 6.01±0.28 |
|  | IDO | 15.31±0.66 | 15.66±0.40 | 15.75±0.60 | 14.97±0.48 |
|  | IGF1 | 3.01±0.63 | 3.52±0.28 | 3.92±0.44 | 3.97±0.60 |
|  | MMP11 | 11.23±0.43 | 9.93±0.42 | 10.32±0.26 | 9.18±0.38 |
|  | Pai-1 | 5.34±0.71 | 6.10±0.61 | 5.24±0.65 | 4.72±0.76 |
|  | αSMA | 3.74±0.70 | 2.78±0.44 | 5.09±0.17 | 5.11±0.23 |
|  | TGFβ | 8.32±0.83 | 7.98±0.43 | 8.16±0.35 | 7.51±0.57 |
|  | TnC | 17.79±0.76 | 15.55±0.45 | 15.31±0.81 | 13.32±0.60 |
|  | UCP1 | 11.37±1.08 | 9.29±1.44 | 6.61±1.47 | 10.08±1.16 |
|  | VE-cadh | 6.86±0.20 | 6.68±0.34 | 6.43±0.28 | 5.70±0.62 |
|  | VCAM | 7.32±0.24 | 7.83±0.13 | 6.42±0.39 | 6.40±0.29^e^ |
|  | VEGFa | 6.39±0.37 | 7.82±0.37 | 6.77±0.32 | 6.82±0.26 |
|  | VEGFR2 | 8.15±0.66 | 8.54±0.32 | 8.37±0.29 | 8.14±0.53 |
|  | VLDLR | 4.46±0.41 | 3.93±0.31 | 6.71±0.39 | 7.59±0.54 |

^a^ vs LFD p<0.05

^b^ vs LFD p<0.01

^c^ vs LFD p<0.001

^d^ vs LF FFL p<0.05

^e^ vs HFD p<0.05

^f^ vs HFD p<0.01

^g^ vs HFD p<0.001
