# Supplementary material for: Obesity modulates the cellular and molecular microenvironment in the peritoneal cavity: implication for ovarian cancer risk
Source: Front Immunol. 2024 Jan 9;14:1323399. doi: 10.3389/fimmu.2023.1323399 (PMC10803595; doi:10.3389/fimmu.2023.1323399)
Supplement: Supplementary file 6 [file Table_5.docx]

**Supplemental Table S5**

**Supplemental Table 5. MOSE-L FFL CT values (determined by RT-PCR Array)**

| Adora1 | 31.145 | Cd27 | 31.304 | Erbb2 | 24.706 | | Ifnar1 | 22.249 |
| --- | --- | --- | --- | --- | --- | --- | --- | --- |
| Ahsg | 31.870 | Cd28 | 30.137 | Erbb2ip | 22.507 | | Ifnar2 | 22.727 |
| Aif1 | 31.560 | Cd4 | 31.653 | F11r | 19.646 | | Ifnb1 | 31.537 |
| Apcs | 32.756 | Cd40 | 33.952 | F2 | 31.677 | | Ifne | 29.873 |
| Apoa2 | 26.907 | Cd40lg | 29.992 | F3 | 25.398 | | Ifng | 32.162 |
| Apol7a | 23.630 | Cd70 | 31.298 | F8 | 27.561 | | Ifngr1 | 23.481 |
| Apol8 | 28.601 | Cd74 | 27.165 | Fasl | 31.585 | | Ifngr2 | 21.603 |
| Areg | 25.969 | Cd86 | 30.400 | Fgf1 | 27.876 | | Ifnk | 31.776 |
| Bcl6 | 24.629 | Cd97 | 23.440 | Fgf10 | 21.992 | | Ik | 25.640 |
| Blnk | 29.328 | Cebpb | 19.445 | Fgf12 | 31.620 | | Il10 | 29.179 |
| Bmp1 | 22.343 | Cer1 | 31.594 | Fgf2 | 23.329 | | Il10ra | 32.233 |
| Bmp2 | 30.705 | Cklf | 24.348 | Fgf3 | 30.765 | | Il10rb | 23.142 |
| Bmp3 | 32.455 | Clcf1 | 22.678 | Fgf4 | 32.625 | | Il11 | 28.532 |
| Bmp7 | 33.833 | Cmtm1 | 32.732 | Fgf5 | 31.638 | | Il11ra1 | 22.753 |
| C3 | 21.282 | Cmtm2a | 31.149 | Fgf6 | 30.615 | | Il12a | 29.255 |
| C3ar1 | 26.907 | Cntfr | 32.299 | Fgf7 | 30.969 | | Il12b | 30.826 |
| Cast | 21.217 | Crp | 33.373 | Fgf8 | 32.265 | | Il12rb1 | 28.654 |
| Ccl1 | 31.340 | Csf1 | 19.785 | Fgf9 | 33.548 | | Il12rb2 | 35.324 |
| Ccl11 | 30.897 | Csf2 | 31.095 | Figf | 25.129 | | Il13 | 30.818 |
| Ccl12 | 30.912 | Csf2ra | 27.723 | Flt3l | 26.133 | | Il13ra1 | 23.731 |
| Ccl17 | 31.510 | Csf3 | 28.831 | Fn1 | 16.819 | | Il13ra2 | 32.293 |
| Ccl19 | 28.232 | Csf3r | 31.685 | Fos | 24.885 | | Il15 | 30.317 |
| Ccl2 | 21.222 | Ctf1 | 24.797 | Fpr1 | 29.599 | | Il15ra | 31.782 |
| Ccl20 | 26.409 | Ctf2 | 29.187 | Gdf1 | 29.531 | | Il16 | 23.241 |
| Ccl22 | 30.649 | Cx3cl1 | 24.459 | Gdf2 | 29.425 | | Il17a | 33.180 |
| Ccl24 | 30.636 | Cx3cr1 | 30.013 | Gdf3 | 30.886 | | Il17b | 29.726 |
| Ccl25 | 25.417 | Cxcl1 | 24.440 | Gdf5 | 30.943 | | Il17c | 29.511 |
| Ccl27a | 25.219 | Cxcl10 | 23.436 | Gdf6 | 24.402 | | Il17d | 33.349 |
| Ccl28 | 31.512 | Cxcl11 | 31.764 | Gdf7 | 29.614 | | Il17f | 28.636 |
| Ccl3 | 29.750 | Cxcl12 | 22.541 | Gdf9 | 29.926 | | Il17ra | 24.514 |
| Ccl4 | 29.794 | Cxcl13 | 30.538 | Gfra1 | 29.587 | | Il17rb | 29.319 |
| Ccl5 | 23.966 | Cxcl14 | 31.505 | Gfra2 | 29.692 | | Il18 | 22.925 |
| Ccl6 | 30.501 | Cxcl15 | 31.904 | Ghr | 22.305 | | Il18r1 | 26.237 |
| Ccl7 | 22.165 | Cxcl16 | 22.511 | Glmn | 24.460 | | Il18rap | 25.250 |
| Ccl8 | 28.269 | Cxcl2 | 31.425 | Gpi1 | 18.309 | | Il19 | 31.599 |
| Ccl9 | 25.824 | Cxcl5 | 20.406 | Gpr68 | 28.913 | | Il1a | 27.600 |
| Ccr1 | 29.997 | Cxcl9 | 29.504 | Grem1 | 26.478 | | Il1b | 33.339 |
| Ccr10 | 27.491 | Cxcr3 | 31.716 | Grem2 | 31.672 | | Il1f10 | 32.808 |
| Ccr2 | 30.465 | Cxcr4 | 34.179 | Grn | 20.138 | | Il1f5 | 30.156 |
| Ccr3 | 30.274 | Cxcr5 | 29.554 | Hdac4 | 23.897 | | Il1f6 | 32.831 |
| Ccr4 | 29.932 | Cxcr6 | 28.382 | Hdac5 | 24.406 | | Il1f8 | 32.529 |
| Ccr5 | 32.763 | Cybb | 32.764 | Hdac7 | 20.136 | | Il1f9 | 32.479 |
| Ccr6 | 30.642 | Cyp26b1 | 30.883 | Hdac9 | 31.275 | | Il1r1 | 23.695 |
| Ccr7 | 30.258 | D17Wsu104  IL-25 | 20.651 | Hrh1 | 28.280 | | Il1r2 | 29.464 |
| Ccr8 | 33.496 | Dock2 | 31.249 | Ifna11 | 30.763 | | Il1rap | 23.709 |
| Ccr9 | 29.969 | Ebi3 | 29.710 | Ifna14 | 29.562 | | Il1rapl2 | 31.389 |
| Ccrl1 | 30.716 | Eda | 32.014 | Ifna2 | 29.405 | | Il1rl1 | 27.373 |
| Ccrl2 | 28.150 | Ephx2 | 32.698 | Ifna4 | 30.267 | | Il1rl2 | 26.580 |
| Cd14 | 25.135 | Epo | 30.384 | Ifna9 | 30.969 | | Il1rn | 27.789 |
| Cd180 | 32.401 | Epor | 27.234 | Ifnab | 29.684 | | Il2 | 31.489 |
| Il20 | 30.876 | Ltb4r1 | 32.742 | Ptx3 | | 21.584 | Tnfsf14 | 30.478 |
| Il20ra | 31.975 | Ly75 | 25.471 | Pxmp2 | | 24.776 | Tnfsf15 | 28.943 |
| Il21 | 32.879 | Ly86 | 30.887 | Reg3a | | 30.303 | Tnfsf18 | 34.412 |
| Il21r | 31.461 | Ly96 | 23.941 | Reg3g | | 32.139 | Tnfsf4 | 32.310 |
| Il22 | 30.829 | Mdk | 28.114 | Ripk2 | | 23.744 | Tnfsf8 | 31.206 |
| Il22ra1 | 24.786 | Mefv | 30.561 | S100a11 | | 16.677 | Tnfsf9 | 24.991 |
| Il22ra2 | 30.945 | Mgll | 27.683 | S100a8 | | 27.635 | Tollip | 22.355 |
| Il23a | 29.567 | Mif | 17.444 | S100b | | 31.243 | Tpst1 | 22.144 |
| Il23r | 31.777 | Mmp25 | 30.303 | Saa4 | | 31.871 | Trap1 | 21.559 |
| Il24 | 30.738 | Mpl | 30.684 | Scg2 | | 30.845 | Ttn | 29.520 |
| Il27 | 29.869 | Mstn | 31.957 | Scube1 | | 30.969 | Tymp | 28.471 |
| Il28ra | 27.013 | Muc4 | 30.616 | Aimp1 | | 20.163 | Vegfa | 22.300 |
| Il2ra | 32.760 | Myd88 | 25.524 | Sdcbp | | 20.656 | Vegfb | 23.354 |
| Il2rb | 30.152 | Nfam1 | 35.264 | Sectm1b | | 30.727 | Vps45 | 24.469 |
| Il2rg | 23.692 | Nfatc3 | 22.579 | Sele | | 32.469 | Xcl1 | 30.748 |
| Il3 | 31.481 | Nfatc4 | 32.773 | Serpina1a | | 29.558 | Xcr1 | 31.688 |
| Il31 | 31.407 | Nfe2l1 | 21.353 | Serpina3n | | 27.895 | Yars | 21.656 |
| Il31ra | 31.848 | Nfkb1 | 23.001 | Serpinf2 | | 28.511 | Gusb | 23.377 |
| Il3ra | Undt | Nfrkb | 24.609 | Sftpd | | 33.016 | Hprt | 20.851 |
| Il4 | 31.590 | Nfx1 | 24.830 | Sigirr | | 26.797 | Hsp90ab1 | 16.632 |
| Il4ra | 27.944 | Nlrp12 | 31.910 | Siglec1 | | 31.505 | Gapdh | 15.758 |
| Il5 | 30.990 | Nmi | 26.723 | Siva1 | | 20.515 | Actb | 15.125 |
| Il5ra | 25.148 | Nodal | 34.290 | Slco1a4 | | 34.212 | MGDC | 34.164 |
| Il6 | 28.962 | Nos2 | 23.570 | Slurp1 | | 28.146 | MGDC | 33.605 |
| Il6ra | 26.607 | Nr3c1 | 22.324 | Socs2 | | 26.202 | MGDC | 33.207 |
| Il6st | 20.499 | Nrg1 | 24.940 | Spaca3 | | 31.766 | RTC | 33.135 |
| Il7 | 30.814 | Ntf3 | 30.537 | Spp1 | | 19.227 | RTC | 34.570 |
| Il7r | 29.351 | Olr1 | 21.671 | Spred1 | | 21.769 | RTC | 38.103 |
| Cxcr1 | 32.591 | Osm | 31.392 | Srgap1 | | 29.574 | PPC | 18.199 |
| Cxcr2 | 27.784 | Osmr | 22.106 | Stab1 | | 26.445 | PPC | 18.170 |
| Il9 | 37.750 | Parp4 | 24.564 | Stat3 | | 23.443 | PPC | 17.955 |
| Il9r | 31.346 | Nampt | 21.221 | Sykb | | 33.296 |  |  |
| Inha | 28.690 | Pdgfa | 24.441 | Tacr1 | | 30.134 |  |  |
| Inhba | 21.870 | Pdgfb | 22.121 | Thpo | | 29.718 |  |  |
| Inhbb | 30.943 | Pdgfc | 23.753 | Tirap | | 23.770 |  |  |
| Ins1 | 31.518 | Pf4 | 30.696 | Tlr1 | | 26.817 |  |  |
| Ins2 | 30.623 | Pglyrp1 | 29.166 | Tlr2 | | 25.308 |  |  |
| Irf4 | 30.753 | Pla2g2d | 31.981 | Tlr3 | | 30.967 |  |  |
| Irf7 | 24.976 | Pla2g7 | 23.775 | Tlr2 | | 23.426 |  |  |
| Itgb2 | 24.895 | Ppbp | 25.182 | Tlr5 | | 24.673 |  |  |
| Itih4 | 29.571 | Prdx5 | 20.507 | Tlr6 | | 26.546 |  |  |
| Kitl | 22.306 | Prg2 | 31.012 | Tlr7 | | 27.618 |  |  |
| Kng1 | 32.499 | Prg3 | 31.596 | Tlr8 | | 32.304 |  |  |
| Lbp | 30.557 | Prl | 29.910 | Tlr9 | | 31.192 |  |  |
| Lefty1 | 22.493 | Prlr | 30.812 | Tnf | | 31.728 |  |  |
| Lefty2 | 29.143 | Procr | 22.863 | Tnfaip6 | | 26.410 |  |  |
| Lepr | 30.497 | Prok2 | 31.882 | Tnfrsf11b | | 30.526 |  |  |
| Lif | 23.949 | Ptafr | 30.980 | Tnfsf10 | | 33.182 |  |  |
| Lifr | 22.425 | Ptgs2 | 23.781 | Tnfsf11 | | 31.394 |  |  |
| Lta | 29.826 | Ptn | 30.849 | Tnfsf13 | | 23.583 |  |  |
| Ltb | 30.754 | Ptpra | 21.528 | Tnfsf13b | | 28.850 |  |  |

**dCT**

| Adora1 | 8.333386 |  |
| --- | --- | --- |
| Ahsg | 9.058386 |  |
| Aif1 | 8.748385 |  |
| Apcs | 9.944386 |  |
| Apoa2 | 4.095385 |  |
| Apol7a | 0.818384 |  |
| Apol8 | 5.789385 |  |
| Areg | 3.157385 |  |
| Bcl6 | 1.817385 |  |
| Blnk | 6.516384 |  |
| Bmp1 | -0.468615 |  |
| Bmp2 | 7.893385 |  |
| Bmp3 | 9.643387 |  |
| Bmp7 | 11.021385 |  |
| C3 | -1.529615 |  |
| C3ar1 | 4.095385 |  |
| Cast | -1.594616 |  |
| Ccl1 | 8.528385 |  |
| Ccl11 | 8.085384 |  |
| Ccl12 | 8.100386 |  |
| Ccl17 | 8.698385 |  |
| Ccl19 | 5.420385 |  |
| Ccl2 | -1.589615 |  |
| Ccl20 | 3.597385 |  |
| Ccl22 | 7.837385 |  |
| Ccl24 | 7.824385 |  |
| Ccl25 | 2.605385 |  |
| Ccl27a | 2.407385 |  |
| Ccl28 | 8.700384 |  |
| Ccl3 | 6.938385 |  |
| Ccl4 | 6.982386 |  |
| Ccl5 | 1.154385 |  |
| Ccl6 | 7.689385 |  |
| Ccl7 | -0.646614 |  |
| Ccl8 | 5.457384 |  |
| Ccl9 | 3.012384 |  |
| Ccr1 | 7.185385 |  |
| Ccr10 | 4.679384 |  |
| Ccr2 | 7.653385 |  |
| Ccr3 | 7.462385 |  |
| Ccr4 | 7.120384 |  |
| Ccr5 | 9.951386 |  |
| Ccr6 | 7.830385 |  |
| Ccr7 | 7.446384 |  |
| Ccr8 | 10.684383 |  |
| Ccr9 | 7.157385 |  |
| Ccrl1 | 7.904385 |  |
| Ccrl2 | 5.338385 |  |
| Cd14 | 2.323385 |  |
| Cd180 | 9.589386 |  |
| Cd27 | 8.492386 |  |
| Cd28 | 7.325384 |  |
| Cd4 | 8.841385 |  |
| Cd40 | 11.140385 |  |
| Cd40lg | 7.180386 |  |
| Cd70 | 8.486385 |  |
| Cd74 | 4.353386 |  |
| Cd86 | 7.588385 |  |
| Cd97 | 0.628386 |  |
| Cebpb | -3.366615 |  |
| Cer1 | 8.782385 |  |
| Cklf | 1.536385 |  |
| Clcf1 | -0.133615 |  |
| Cmtm1 | 9.920383 |  |
| Cmtm2a | 8.337385 |  |
| Cntfr | 9.487385 |  |
| Crp | 10.561386 |  |
| Csf1 | -3.026615 |  |
| Csf2 | 8.283384 |  |
| Csf2ra | 4.911385 |  |
| Csf3 | 6.019384 |  |
| Csf3r | 8.873385 |  |
| Ctf1 | 1.985386 |  |
| Ctf2 | 6.375385 |  |
| Cx3cl1 | 1.647385 |  |
| Cx3cr1 | 7.201386 |  |
| Cxcl1 | 1.628386 |  |
| Cxcl10 | 0.624386 |  |
| Cxcl11 | 8.952385 |  |
| Cxcl12 | -0.270615 |  |
| Cxcl13 | 7.726385 |  |
| Cxcl14 | 8.693384 |  |
| Cxcl15 | 9.092384 |  |
| Cxcl16 | -0.300615 |  |
| Cxcl2 | 8.613384 |  |
| Cxcl5 | -2.405615 |  |
| Cxcl9 | 6.692385 |  |
| Cxcr3 | 8.904385 |  |
| Cxcr4 | 11.367386 |  |
| Cxcr5 | 6.742386 |  |
| Cxcr6 | 5.570385 |  |
| Cybb | 9.952385 |  |
| Cyp26b1 | 8.071384 |  |
| D17Wsu104e | -2.160616 |  |
| Dock2 | 8.437386 |  |
| Ebi3 | 6.898384 |  |
| Eda | 9.202385 |  |
| Ephx2 | 9.886387 |  |
| Epo | 7.572386 |  |
| Epor | 4.422384 |  |
| Erbb2 | 1.894384 |  |
| Erbb2ip | -0.304615 |  |
| F11r | -3.165615 |  |
| F2 | 8.865385 |  |
| F3 | 2.586386 |  |
| F8 | 4.749386 |  |
| Fasl | 8.773384 |  |
| Fgf1 | 5.064385 |  |
| Fgf10 | -0.819614 |  |
| Fgf12 | 8.808386 |  |
| Fgf2 | 0.517386 |  |
| Fgf3 | 7.953384 |  |
| Fgf4 | 9.813385 |  |
| Fgf5 | 8.826386 |  |
| Fgf6 | 7.803385 |  |
| Fgf7 | 8.157385 |  |
| Fgf8 | 9.453384 |  |
| Fgf9 | 10.736385 |  |
| Figf | 2.317385 |  |
| Flt3l | 3.321384 |  |
| Fn1 | -5.992615 |  |
| Fos | 2.073385 |  |
| Fpr1 | 6.787386 |  |
| Gdf1 | 6.719385 |  |
| Gdf2 | 6.613384 |  |
| Gdf3 | 8.074385 |  |
| Gdf5 | 8.131386 |  |
| Gdf6 | 1.590385 |  |
| Gdf7 | 6.802385 |  |
| Gdf9 | 7.114386 |  |
| Gfra1 | 6.775385 |  |
| Gfra2 | 6.880384 |  |
| Ghr | -0.506615 |  |
| Glmn | 1.648384 |  |
| Gpi1 | -4.502615 |  |
| Gpr68 | 6.101385 |  |
| Grem1 | 3.666386 |  |
| Grem2 | 8.860386 |  |
| Grn | -2.673614 |  |
| Hdac4 | 1.085384 |  |
| Hdac5 | 1.594385 |  |
| Hdac7 | -2.675615 |  |
| Hdac9 | 8.463385 |  |
| Hrh1 | 5.468386 |  |
| Ifna11 | 7.951386 |  |
| Ifna14 | 6.750385 |  |
| Ifna2 | 6.593386 |  |
| Ifna4 | 7.455385 |  |
| Ifna9 | 8.157385 |  |
| Ifnab | 6.872385 |  |
| Ifnar1 | -0.562614 |  |
| Ifnar2 | -0.084616 |  |
| Ifnb1 | 8.725386 |  |
| Ifne | 7.061384 |  |
| Ifng | 9.350384 |  |
| Ifngr1 | 0.669386 |  |
| Ifngr2 | -1.208614 |  |
| Ifnk | 8.964384 |  |
| Ik | 2.828384 |  |
| Il10 | 6.367386 |  |
| Il10ra | 9.421387 |  |
| Il10rb | 0.330385 |  |
| Il11 | 5.720385 |  |
| Il11ra1 | -0.058615 |  |
| Il12a | 6.443384 |  |
| Il12b | 8.014385 |  |
| Il12rb1 | 5.842384 |  |
| Il12rb2 | 12.188385 |  |
| Il13 | 8.006386 |  |
| Il13ra1 | 0.919386 |  |
| Il13ra2 | 9.481384 |  |
| Il15 | 7.505384 |  |
| Il15ra | 8.970385 |  |
| Il16 | 0.429384 |  |
| Il17a | 10.368385 |  |
| Il17b | 6.914385 |  |
| Il17c | 6.699385 |  |
| Il17d | 10.537384 |  |
| Il17f | 5.824385 |  |
| Il17ra | 1.702385 |  |
| Il17rb | 6.507385 |  |
| Il18 | 0.113384 |  |
| Il18r1 | 3.425385 |  |
| Il18rap | 2.438385 |  |
| Il19 | 8.787386 |  |
| Il1a | 4.788385 |  |
| Il1b | 10.527386 |  |
| Il1f10 | 9.996384 |  |
| Il1f5 | 7.344385 |  |
| Il1f6 | 10.019386 |  |
| Il1f8 | 9.717384 |  |
| Il1f9 | 9.667385 |  |
| Il1r1 | 0.883385 |  |
| Il1r2 | 6.652386 |  |
| Il1rap | 0.897385 |  |
| Il1rapl2 | 8.577385 |  |
| Il1rl1 | 4.561384 |  |
| Il1rl2 | 3.768385 |  |
| Il1rn | 4.977385 |  |
| Il2 | 8.677385 |  |
| Il20 | 8.064385 |  |
| Il20ra | 9.163385 |  |
| Il21 | 10.067387 |  |
| Il21r | 8.649385 |  |
| Il22 | 8.017386 |  |
| Il22ra1 | 1.974384 |  |
| Il22ra2 | 8.133385 |  |
| Il23a | 6.755384 |  |
| Il23r | 8.965385 |  |
| Il24 | 7.926386 |  |
| Il27 | 7.057385 |  |
| Il28ra | 4.201386 |  |
| Il2ra | 9.948383 |  |
| Il2rb | 7.340385 |  |
| Il2rg | 0.880384 |  |
| Il3 | 8.669386 |  |
| Il31 | 8.595385 |  |
| Il31ra | 9.036385 |  |
| Il3ra | 12.188385 |  |
| Il4 | 8.778385 |  |
| Il4ra | 5.132385 |  |
| Il5 | 8.178385 |  |
| Il5ra | 2.336386 |  |
| Il6 | 6.150385 |  |
| Il6ra | 3.795385 |  |
| Il6st | -2.312614 |  |
| Il7 | 8.002384 |  |
| Il7r | 6.539385 |  |
| Cxcr1 | 9.779385 |  |
| Cxcr2 | 4.972385 |  |
| Il9 | 12.188385 |  |
| Il9r | 8.534386 |  |
| Inha | 5.878386 |  |
| Inhba | -0.941614 |  |
| Inhbb | 8.131386 |  |
| Ins1 | 8.706385 |  |
| Ins2 | 7.811384 |  |
| Irf4 | 7.941385 |  |
| Irf7 | 2.164385 |  |
| Itgb2 | 2.083386 |  |
| Itih4 | 6.759384 |  |
| Kitl | -0.505615 |  |
| Kng1 | 9.687386 |  |
| Lbp | 7.745384 |  |
| Lefty1 | -0.318615 |  |
| Lefty2 | 6.331385 |  |
| Lepr | 7.685385 |  |
| Lif | 1.137384 |  |
| Lifr | -0.386616 |  |
| Lta | 7.014385 |  |
| Ltb | 7.942385 |  |
| Ltb4r1 | 9.930386 |  |
| Ly75 | 2.659386 |  |
| Ly86 | 8.075384 |  |
| Ly96 | 1.129385 |  |
| Mdk | 5.302385 |  |
| Mefv | 7.749386 |  |
| Mgll | 4.871386 |  |
| Mif | -5.367615 |  |
| Mmp25 | 7.491385 |  |
| Mpl | 7.872385 |  |
| Mstn | 9.145386 |  |
| Muc4 | 7.804384 |  |
| Myd88 | 2.712385 |  |
| Nfam1 | 12.188385 |  |
| Nfatc3 | -0.232614 |  |
| Nfatc4 | 9.961384 |  |
| Nfe2l1 | -1.458614 |  |
| Nfkb1 | 0.189385 |  |
| Nfrkb | 1.797384 |  |
| Nfx1 | 2.018385 |  |
| Nlrp12 | 9.098385 |  |
| Nmi | 3.911385 |  |
| Nodal | 11.478386 |  |
| Nos2 | 0.758385 |  |
| Nr3c1 | -0.487616 |  |
| Nrg1 | 2.128386 |  |
| Ntf3 | 7.725386 |  |
| Olr1 | -1.140615 |  |
| Osm | 8.580385 |  |
| Osmr | -0.705614 |  |
| Parp4 | 1.752384 |  |
| Nampt | -1.590614 |  |
| Pdgfa | 1.629385 |  |
| Pdgfb | -0.690615 |  |
| Pdgfc | 0.941385 |  |
| Pf4 | 7.884384 |  |
| Pglyrp1 | 6.354385 |  |
| Pla2g2d | 9.169386 |  |
| Pla2g7 | 0.963385 |  |
| Ppbp | 2.370384 |  |
| Prdx5 | -2.304615 |  |
| Prg2 | 8.200384 |  |
| Prg3 | 8.784386 |  |
| Prl | 7.098385 |  |
| Prlr | 8.000385 |  |
| Procr | 0.051386 |  |
| Prok2 | 9.070385 |  |
| Ptafr | 8.168385 |  |
| Ptgs2 | 0.969385 |  |
| Ptn | 8.037386 |  |
| Ptpra | -1.283615 |  |
| Ptx3 | -1.227615 |  |
| Pxmp2 | 1.964384 |  |
| Reg3a | 7.491385 |  |
| Reg3g | 9.327385 |  |
| Ripk2 | 0.932385 |  |
| S100a11 | -6.134615 |  |
| S100a8 | 4.823385 |  |
| S100b | 8.431385 |  |
| Saa4 | 9.059385 |  |
| Scg2 | 8.033384 |  |
| Scube1 | 8.157385 |  |
| Aimp1 | -2.648615 |  |
| Sdcbp | -2.155615 |  |
| Sectm1b | 7.915384 |  |
| Sele | 9.657387 |  |
| Serpina1a | 6.746386 |  |
| Serpina3n | 5.083386 |  |
| Serpinf2 | 5.699385 |  |
| Sftpd | 10.204384 |  |
| Sigirr | 3.985386 |  |
| Siglec1 | 8.693384 |  |
| Siva1 | -2.296616 |  |
| Slco1a4 | 11.400387 |  |
| Slurp1 | 5.334385 |  |
| Socs2 | 3.390385 |  |
| Spaca3 | 8.954386 |  |
| Spp1 | -3.584616 |  |
| Spred1 | -1.042616 |  |
| Srgap1 | 6.762384 |  |
| Stab1 | 3.633385 |  |
| Stat3 | 0.631386 |  |
| Sykb | 10.484386 |  |
| Tacr1 | 7.322386 |  |
| Thpo | 6.906385 |  |
| Tirap | 0.958386 |  |
| Tlr1 | 4.005384 |  |
| Tlr2 | 2.496386 |  |
| Tlr3 | 8.155384 |  |
| Tlr4 | 0.614386 |  |
| Tlr5 | 1.861385 |  |
| Tlr6 | 3.734385 |  |
| Tlr7 | 4.806385 |  |
| Tlr8 | 9.492386 |  |
| Tlr9 | 8.380384 |  |
| Tnf | 8.916386 |  |
| Tnfaip6 | 3.598385 |  |
| Tnfrsf11b | 7.714384 |  |
| Tnfsf10 | 10.370384 |  |
| Tnfsf11 | 8.582384 |  |
| Tnfsf13 | 0.771385 |  |
| Tnfsf13b | 6.038385 |  |
| Tnfsf14 | 7.666386 |  |
| Tnfsf15 | 6.131386 |  |
| Tnfsf18 | 11.600384 |  |
| Tnfsf4 | 9.498386 |  |
| Tnfsf8 | 8.394384 |  |
| Tnfsf9 | 2.179384 |  |
| Tollip | -0.456615 |  |
| Tpst1 | -0.667616 |  |
| Trap1 | -1.252615 |  |
| Ttn | 6.708386 |  |
| Tymp | 5.659386 |  |
| Vegfa | -0.511616 |  |
| Vegfb | 0.542385 |  |
| Vps45 | 1.657385 |  |
| Xcl1 | 7.936384 |  |
| Xcr1 | 8.876385 |  |
| Yars | -1.155615 |  |

**High:**

**Cebpb** transcription factor, Activity of this protein is important in the regulation of genes involved in immune and inflammatory responses,

**CSF1** secreted cytokine, hematopoietic differentiation into macrophage or others, survival of macrophages; in cancer cells ERK activation and proliferation

**Cxcl5** cytokine, epithelial, neutrophil chemotaxis

**D17Wsu104—IL25** cytokine, pro-inflammatory, Th2 type, IL8 stimulation, NfkB activation

**F11r** tight junctions in epithelium, cell-cell adhesion

**Fn1** fibronectin, extracellular matrix, integrin binding, induction of mesenchymal phenotype (LENGYEL)

**Gpi1** glucose phosphate isomerase 1; can function in plasma as glycolytic enzyme (glucose-6-phosphate isomerase) that interconverts glucose-6-phophsate and fructose-6-phosphate; involved in pentose phosphate pathway. Extracellularly, the encoded protein (also referred to as neuroleukin) functions as a neurotrophic factor that promotes survival of skeletal motor neurons and sensory neurons, and as a lymphokine that induces immunoglobulin secretion. The encoded protein is also referred to as autocrine motility factor based on an additional function as a tumor-secreted cytokine and angiogenic factor.

Secretory in cancer, called autoctrine motility factor, stimulates growth and motility and metastasis. Activation of MAPK and PI3K

also participates in a [positive feedback](https://en.wikipedia.org/wiki/Positive_feedback) loop with [HER2](https://en.wikipedia.org/wiki/HER2), a major breast cancer therapeutic target, as GPI enhances HER2 expression and HER2 overexpression enhances GPI expression, and so on. As a result, GPI activity likely confers resistance in breast cancer cells against HER2-based therapies using [Herceptin](https://en.wikipedia.org/wiki/Herceptin)/Trastuzumab, and should be considered as an additional target when treating patients

**Mif** inflammatory cytokine; binds to CD74 on immune cells and trigger acute immune response

**S100a11** calgizzarin: motility, invasion tubulin polymerization

Upregulated in ovcar: KD reduced growth and invasion (Liu Y and Gao, Exp Ther Med 2015)

**Spp1** osteopontin: proliferation, resistance to cell death, replicative immortality, angiogenesis, deregulation of cellular energetics, promotion of inflammation, metastasis

**Moderate:<25**

Apol7a

Bcl6

Bmp1

C3

Cast

Ccl2

Ccl5

Ccl7

Cd97

Cklf

Clcf1

Ctf1

Cx3cl1

Cxcl1

Cxcl10

Cxcl10

Cxcl12

Cxcl16

Erbb2

Erbb2ip

Fgf10

Fgf2

Fos

Gdf6

Ghr

Glmn

Grn

Hdac4

Hdac5

Hdac7

Ifnar1

Ifnar2

Ifngr1

Ifngr2

Il10rb

Il11ra1

Il16

Il18

Il1r1

Il1rap

Il22ra1

Il2rg

Il6st

Inhba

Irf7

Itgb2

Irf7

Itgb2

Kitl

Lefty1

Lif

Lifr

Ly96

Nfatc3

Nfe2l1

Nfrkb

Nfrkb

Nfx1

Nos2

Nr3c1

Nrg1

Olr1

Osmr

Osmr

Parp4

Nampt

Pdgfa

Pdgfb

Pdgfc

Pla2g7

Procr

Ptgs2

Ptpra

Ptx3

Pxmp2

Ripk2

Aimp1

Sdcbp

Siva1

Spred1

Stat3

Tirap

Tlr2

Tlr5

Tnfsf13

Tnfsf9

Tollip

Tpst1

Trap1

Vegfa

Vegfb

Vps45

Moderate>25

Apoa2

Areg

C3ar1

Ccl20

Ccl25

Ccl27a

Ccl8

Ccl9

Ccr10

Ccrl2

Cd14

Cd74

Csf2ra

Csf3

Cxcl9

Cxcr5

Cxcr6

Ebi3

Epor

F3

F8

Fgf1

Figf

Flt3l

Fpr1

Gdf1

Gdf2

Gpr68

Grem1

Hrh1

Ifna14

Ifna2

Ifnab

Ifne

Ik

Il10

Il11

Il17b

Il17c

Il18r1

Il18rap

Il1a

Il1rl1

Il1rl2

Il1rn

Il28ra

Il4ra

Il5ra

Il6

Il6ra

Il7r

Cxcr2

Inha

Ly75

Mdk

Mgll

Myd88

Nmi

Ppbp

S100a8

Serpina1a

Serpina3n

Serpinf2

Sigirr

Slurp1

Socs2

Srgap1

Stab1

Thpo

Tlr1

Tlr2

Tlr6

Tlr7

Tnfaip6

Tnfsf13b

Tnfsf15

Tymp
