# Supplementary material for: Obesity modulates the cellular and molecular microenvironment in the peritoneal cavity: implication for ovarian cancer risk
Source: Front Immunol. 2024 Jan 9;14:1323399. doi: 10.3389/fimmu.2023.1323399 (PMC10803595; doi:10.3389/fimmu.2023.1323399)
Supplement: Supplementary file 7 [file Table_6.docx]

**SUPPLEMENTAL TABLE S6**

Table S6: Gene expression levels in the PSF in mice fed a low-fat (LFD) or high-fat diet (HFD) and injected with the ovarian cancer cells (MOSE-L_TIC_*_v_*) (mean ΔCT ±SEM)

|  | LFD | HDF | LFD MOSE-L_TIC_*_v_* | HFD MOSE-L_TIC_*_v_* |
| --- | --- | --- | --- | --- |
| Ccl2 | 10.37±0.19 | 7.48±0.39^c^ | 7.99±0.11^c^ | 5.47±0.46^c,f,h,^ |
| Ccl3 | 14.93±0.10 | 11.78±0.70^c^ | 12.36±0.34^c^ | 9.02±0.36^c,f,h^ |
| Ccl4 | 13.52±0.28 | 11.57±0.33^c^ | 9.71±0.26^c^ | 7.40±0.62^c,e,i^ |
| Cxcl12 | 8.61±0.18 | 8.43±0.20^a^ | 11.33±0.79 | 10.50±0.84 |
| Cxcl13 | 1.16±0.27 | 2.29±0.16 | 4.23±0.26 ^c^ | 4.31±0.94 ^c,g^ |
| Cx3cl1 | 15.52±0.35 | 16.26±0.18 | 14.04±0.57 | 13.24±0.55^a,h^ |
| Cxcr2 | 17.28±1.51 | 17.00±1.51 | 9.78±0.82^c^ | 9.11±0.95^c,i^ |
| Ccr5 | 8.98±0.18 | 7.88±0.23 | 6.83±0.16^c^ | 6.75±0.54^c^ |
| Cd19 | 3.24±0.11 | 3.41±0.18 | 4.51±0.20^b^ | 4.54±0.38^b,g^ |
| Cd68 | 4.26±0.22 | 4.42±0.12 | 3.65±0.33 | 3.15±0.29^a,g^ |
| Fizz1 | 12.14±0.52 | 12.69±0.25 | 15.18±1.56 | 15.85±1.05 |
| GM-CSF | 20.60±0.07 | 17.71±0.82^a^ | 18.56±0.52 | 16.27±0.34^c^ |
| Ifng | 16.01±0.26 | 13.56±0.20^c^ | 13.71±0.43^c^ | 12.11±0.42^c,d,g^ |
| Igf1 | 13.82±0.43 | 13.17±0.29 | 13.60±0.33 | 12.95±0.40 |
| ILb | 13.25±0.45 | 11.11±0.23 | 7.34±0.52 | 4.76±0.71 |
| IL10 | 21.19±0.35 | 19.79±0.46 | 21.29±0.71 | 18.32±0.43 |
| IL21 | 17.01±0.26 | 17.48±0.60 | 17.69±0.71 | 17.40±0.44 |
| IL6 | 16.41±0.39 | 15.62±0.52 | 14.71±0.35 | 13.48±0.71 |
| Ly6a | 3.34±0.09 | 3.18±0.22 | 4.17±0.28 | 3.96±0.30 |
| Mcsf | 13.57±0.18 | 13.83±0.13 | 12.24±0.74 | 10.95±0.66^a,h^ |
| Ido | 14.41±0.17 | 14.42±0.12 | 14.76±0.45 | 13.55±0.34 |
| Rory | 22.84±0.43 | 22.39±0.43 | 22.96±0.48 | 23.10±0.43 |
| Tgfb | 4.72±0.16 | 4.31±0.11 | 4.65±0.26 | 4.08±0.09 |
| Tnfa | 9.47±0.47 | 7.88±0.23^a^ | 6.83±0.16^c^ | 6.76±0.54^c^ |
| Tlr1 | 8.36±0.11 | 8.25±0.25 | 8.72±0.07 | 8.48±0.10 |
| Tlr2 | 8.13±0.11 | 8.02±0.18 | 7.67±0.22 | 6.82±0.33^c^ |
| Tlr3 | 11.13±0.15 | 11.28±0.12 | 10.88±0.34 | 10.08±0.21^b,h^ |
| Tlr4 | 8.96±0.12 | 9.28±0.10 | 9.02±0.23 | 8.41±0.37 |
| Tnc | 18.30±0.53 | 18.64±0.86 | 17.12±0.92 | 17.37±0.54 |
| Ido | 14.41±0.17 | 14.42±0.12 | 14.76±0.45 | 13.55±0.34 |
| Tbet | 12.25±0.28 | 11.18±0.37 | 11.30±0.26 | 10.93±0.021 |
| Ym1 | 10.42±0.64 | 11.52±0.77 | 7.19±0.80 | 7.57±1.24^g^ |
| Cd36 | 15.91±0.26 | 15.45±0.41 | 16.92±0.77 | 16.52±0.07 |
| Cd4 | 9.97±0.14 | 9.69±0.33 | 8.76±0.12 | 8.83±0.21^b,e^ |
| Cd8 | 10.91±0.27 | 10.30±0.29 | 8.95±0.23^c^ | 8.58±0.15^c,i^ |

^a^p<0.05 vs LFD; ^b^p<0.01 vs LFD; ^c^p<0.001 vs LFD; ^d^p<0.05 vs LFD-MOSE-L_TIC_*_v_*; ^e^p<0.01 vs LFD-MOSE-L_TIC_*_v_*; ^f^p<0.001 vs LFD-MOSE-L_TIC_*_v_*; ^g^p<0.05 vs HFD; ^h^p<0.01 vs HFD; ^i^p<0.001 vs HFD
